# Molecular Networking-Based Metabolome and Bioactivity Analyses of Marine-Adapted Fungi Co-cultivated With Phytopathogens

**DOI:** 10.3389/fmicb.2018.02072

**Published:** 2018-09-06

**Authors:** Ernest Oppong-Danquah, Delphine Parrot, Martina Blümel, Antje Labes, Deniz Tasdemir

**Affiliations:** ^1^GEOMAR Centre for Marine Biotechnology (GEOMAR-Biotech), Research Unit Marine Natural Products Chemistry, GEOMAR Helmholtz Centre for Ocean Research Kiel, Kiel, Germany; ^2^Department of Energy and Biotechnology, Flensburg University of Applied Sciences, Flensburg, Germany; ^3^Kiel University, Kiel, Germany

**Keywords:** marine-adapted fungi, co-culture, biocontrol, phytopathogen, molecular networking, metabolomics

## Abstract

Fungi represent a rich source of bioactive metabolites and some are marketed as alternatives to synthetic agrochemicals against plant pathogens. However, the culturability of fungal strains in artificial laboratory conditions is still limited and the standard mono-cultures do not reflect their full spectrum chemical diversity. Phytopathogenic fungi and bacteria have successfully been used in the activation of cryptic biosynthetic pathways to promote the production of new secondary metabolites in co-culture experiments. The aim of this study was to map the fungal diversity of Windebyer Noor, a brackish lake connected to Baltic Sea (Germany), to induce the chemical space of the isolated marine-adapted fungi by co-culturing with phytopathogens, and to assess their inhibitory potential against six commercially important phytopathogens. Out of 123 marine-adapted fungal isolates obtained, 21 were selected based on their phylogenetic and metabolite diversity. They were challenged with two phytopathogenic bacteria (*Pseudomonas syringae* and *Ralstonia solanacearum*) and two phytopathogenic fungi (*Magnaporthe oryzae* and *Botrytis cinerea*) on solid agar. An in-depth untargeted metabolomics approach incorporating UPLC–QToF–HRMS/MS-based molecular networking (MN), *in silico* MS/MS databases, and manual dereplication was employed for comparative analysis of the extracts belonging to nine most bioactive co-cultures and their respective mono-cultures. The phytopathogens triggered interspecies chemical communications with marine-adapted fungi, leading to the production of new compounds and enhanced expression of known metabolites in co-cultures. MN successfully generated a detailed map of the chemical inventory of both mono- and co-cultures. We annotated overall 18 molecular clusters (belonging to terpenes, alkaloids, peptides, and polyketides), 9 of which were exclusively produced in co-cultures. Several clusters contained compounds, which could not be annotated to any known compounds, suggesting that they are putatively new metabolites. Direct antagonistic effects of the marine-adapted fungi on the phytopathogens were observed and anti-phytopathogenic activity was demonstrated.The untargeted metabolomics approach combined with bioactivity testing allowed prioritization of two co-cultures for purification and characterization of marine fungal metabolites with crop-protective activity. To our knowledge, this is the first study employing plant pathogens to challenge marine-adapted fungi.

## Introduction

Phytopathogens that infect crops and vegetables cause a significant loss of field crops, threatening global food security and the economic strength of the countries concerned (Knight et al., 1997; Darji et al., 2012). The estimated loss of major crop species such as rice, wheat, maize, potato, and soybean to phytopathogenic fungi in the years 2009 and 2010 would have sufficed to supply nutrition of 600 million people (Fisher et al., 2012). The famous Irish potato famine that caused starvation, death, and migration of millions of Irish people to overseas countries in the 19th century, was caused by the oomycete *Phytophthora infestans* (Zadoks, 2017). Microbial phytopathogens are usually controlled through the application of aggressive agrochemicals or synthetic pesticides. However, extensive use of these pesticides has caused the development of resistance toward common agrochemicals by phytopathogens (Sparks and Lorsbach, 2017). Synthetic pesticides are associated with toxicity in humans and the environment (Aktar et al., 2009; Singh et al., 2016; Valcke et al., 2017), highlighting the increasing demand for alternative, environmentally friendly biocontrol agents. Microorganisms are a historical source of bioactive compounds (Demain, 2014). Fungi are regarded as highly prolific producers of secondary metabolites (SMs), some producing over 100 compounds in a single culture medium (Nielsen and Larsen, 2015). Concerning agricultural applications, strobilurin-type polyketides that originate from terrestrial fungus *Strobilurus tenacellus* (Kim and Hwang, 2007) are widely applied as pesticides. Although a few SMs obtained from marine-derived fungi (*Chaetomium* sp., *Microsphaeropsis* sp., *Penicillium* sp., and *Coniothyrium* sp.) with anti-phytopathogenic activities (Höller et al., 1999; Lösgen et al., 2007; Wen et al., 2014), the real potential of marine-derived fungi as source of natural biocontrol agents has largely remained untapped.

In natural environments, like the marine realm, fungi exist in complex multispecies communities. This requires adaptation to changing environmental conditions, competition, and communication with other co-occurring microorganisms (Fischer et al., 2016), mediated by chemical signaling often involving SMs. Fungal genomics revealed that the majority of biosynthetic gene clusters (BGCs) remain silent or under-expressed under conventional single culture conditions (Valayil, 2016). Co-cultivation of two or more different microorganisms from the same habitat has emerged as a valuable approach for generation of a competitive environment, thus activating otherwise silent biosynthetic pathways to increase chemical diversity (Bertrand et al., 2013; Yu et al., 2016). Co-cultivation of multiple microorganisms is well known to trigger the production of new antibiotics (Netzker et al., 2018).

During infection, phytopathogens interact with both the plant and its associated microbiota, eliciting the production of SMs for defense and signaling, e.g., by pathogen-/microbe-associated molecular patterns (PAMP/MAMP) (Hardoim et al., 2015; Luti et al., 2016). Phytopathogens are also in constant competition with other (phyto)pathogens co-habiting their environment (Müller et al., 2012). This competitive nature renders phytopathogens promising model organisms and challengers in co-cultivation studies. A recent co-cultivation study between the phytopathogenic fungus *B. cinerea* and a wide variety of fungi from different environments led to the discovery of novel antifungal compounds (Serrano et al., 2017). In another example, the frosty pod rot cocoa disease-causing fungus, *Moniliophthora roreri*, elicited the production of the antifungal agents T39 butenolide, harzianolide, and sorbicillinol in *Trichoderma harzianum* (Tata et al., 2015). Hence, co-cultivation of fungi obtained from environmental samples with phytopathogens in a single confined environment is a unique and promising technique for induction and enhancement of chemically diverse, bioactive metabolites, including anti-phytopathogenic agents.

Microbial natural product discovery is limited by rediscovery of the known chemical structures (Bertrand et al., 2013). This problem can be addressed by (i) an efficient dereplication strategy prior to purification work, (ii) exploration of so far untapped or understudied environments, and (iii) application of strategies to awaken silent BGCs to yield structurally diverse SMs (Lavoie et al., 2017). Marine and brackish environments often represent hostile habitats characterized by rapid changes in abiotic conditions. Such stresses trigger the production of structurally and functionally versatile and unique SMs by both marine macro- and microorganisms (Reen et al., 2015; Romano et al., 2017). The Windebyer Noor, classified as an electrolyte-rich “beach lake” (marine type 88.1)^1^, is located at the northwest German Baltic Sea coast. This brackish water body has an underground connection to the Baltic Sea leading to salinity fluctuations. The presence of high amounts of degrading plant materials makes such environments a good source for searching potentially new and uncommon fungi.

In this study, we pursued a co-cultivation approach with economically relevant phytopathogens as a strategy to expand the chemical space of cultivable fungi. Marine-adapted fungi were co-cultivated with two bacterial (*P. syringae* and *R. solanacearum*) and two fungal phytopathogens (*M. oryzae* and *B. cinerea*) in order to increase the chemical diversity and to stimulate the production of anti-phytopathogenic compounds. The crude organic extracts of single and co-cultures were tested for growth inhibitory activity against six economically relevant phytopathogens. An extensive comparative untargeted metabolomics approach using the HRMS/MS-based molecular networking (MN) (Wang et al., 2016) combined with use of an *in silico* MS/MS database–Universal Natural Product Database (ISDB–UNPD) (Allard et al., 2016) and manual dereplication (using commercial and open access databases) was performed to analyze the most promising co-cultures and their single cultures. This allowed comparative annotation of chemical inventories of both mono- and co-cultures and prioritization of co-culture pairs for in-depth studies.

## Materials and Methods

### Sampling

Samples were obtained from the mouth of the Windebyer Noor (pH 6–6.5, temperature 11.7°C). The Windebyer Noor, which used to be part of the Eckernförder Bight (10°37.330′E, 54°19.330′N) at the northwest German Baltic coast, has underground connections to the open sea through culverts (Steinhardt and Selig, 2011; **Supplementary Figure 1**). It represents a brackish water environment with gradual changes of salinity and temperature. Agricultural practices within catchment areas of the Windebyer Noor have resulted in increased nutrient influx and high amounts of degrading plant materials. Higher wind velocities often generate seafoam which is a good inoculum source for ascomycetes (Overy et al., 2014). Sampling was performed in November 2015. Seawater, seafoam, sediment, as well as the surface of freely swimming decaying plant materials (seeds, twigs, leaves, and driftwood) with a microbiota adapted to brackish environment, were sampled. Water and foam samples were fetched with sterile glass bottles. For sediment sampling, 50 mL sterile Falcon tubes were used for coring the uppermost 10 cm of sediment. Plant materials were collected with sterile bags and sealed immediately to exclude contamination. Driftwood pieces were scraped off by a sterile scalpel into sterile 50 mL Falcon tubes. Samples were placed into a cool box, transported to the laboratory, and immediately processed.

### Inoculation and Isolation of Fungal Strains

Four different solid culture media were used for isolation of fungal strains. All media were enriched with 100 μg/mL streptomycin and 60 μg/mL of penicillin to suppress bacterial growth. Selected media for isolation included GPY medium [glucose monohydrate 1 g, peptone 0.5 g, yeast extract 0.1 g, sodium chloride 15 g, agar 13 g, and deionized water (dH_2_O) *ad* 1 L: pH = 7.2], modified Wickerham medium (WSP: Tropic Marine salt 30 g, glucose 10 g, peptone from soymeal 5 g, yeast extract 3 g, malt extract 3 g, agar 15 g, and dH_2_O *ad* 1 L), PCA medium (carrot 20 g, potato 20 g, NaCl 15 g, agar 15 g, dH_2_O *ad* 1 L), and Hastings medium (HS: Na_2_HPO_4_ × 12H_2_O 9.35 g, K_2_HPO_4_ 1 g, (NH_4_)_2_SO_4_ 0.5 g, MgSO_4_ × 7H_2_O 0.21 g, NaCl 30 g, tryptone 5 g, yeast extract 3 g, glycerol 2 mL, agar 18 g, and dH_2_O *ad* 1 L) (Atlas, 2010).

Samples from plant materials such as leaves, twigs, and wood scrapings were placed in a sterile petri dish and cut into small pieces (about 1 cm in length) by sterilized forceps and scalpel. A piece of each sample was directly inoculated on four different agar media by gentle streaking and finally leaving the piece on the agar plate. A second piece of each sample was transferred to a 2 mL innuSPEED lysis tube type S containing 0.4–0.6 mm ceramic beads (Analytik Jena, Jena, Germany). 600 μL of sterile 0.9% saline was added to the sample and the lysis tube was gently agitated for about 2 min at 15 Hertz (15/s) with a Retsch mill model MM200 (Retsch GmbH, Haan, Germany). High-speed mixing was avoided in order to prevent the destruction of fungal cells. Seafoam, sediment, and water samples were homogenized in the same way (600 μL used and agitation with mixer not necessary). Subsequently, two dilutions (10^-1^ and 10^-2^) were prepared in sterile saline for inoculation. Inoculation was performed by plating 100 μL of the dilutions as well as from the undiluted sample on the above-mentioned four agar media. Plates were incubated at 22°C in the dark. After 7 days of incubation, strain purification started. Single, morphologically different colonies were picked from the original media and propagated on the same media until pure cultures were obtained. For cryopreservation, each pure culture was stored in liquid nitrogen using Microbank^TM^ (PRO-LAB Diagnostics, Richmond Hill, Canada). Phytopathogenic strains used in this work are: *Erwinia amylovora* DSM 50901, *Pseudomonas syringae* pv. *aptata* DSM 50252, *R. solanacearum* DSM 9544, *Xanthomonas campestris* DSM 2405, *B. cinerea* DSM 5145, and *M. oryzae* DSM 62938 purchased from Deutsche Sammlung für Mikroorganismen und Zellkulturen (DSMZ, Braunschweig, Germany), whereas *P. infestans* CBS 120920 and *Septoria tritici* CBS 115941 were obtained from Westerdijk Fungal Biodiversity Institute/Centraal bureau voor Schimmelcultures (Utrecht, Netherlands).

### Identification of Fungal Strains

Fungal strain identification was based on fungal morphology and molecular analysis (PCR amplification and Sanger sequencing) of the ITS1-2 region, which is the generally accepted fungal phylogenetic marker (Schoch et al., 2012; Raja et al., 2017). Amplification and sequencing of the 18S rRNA gene were performed, when amplification of the ITS region failed. Genomic DNA was obtained by simple cell lysis in nuclease-free water with a Retsch mill model MM200 (Retsch GmbH, Haan, Germany). One microliter of this DNA extract was used as template for PCR amplifying the ITS1-5.8S-ITS2 region or the 18S rRNA gene with the respective primers (White et al., 1990) in a total reaction volume of 25 μL [12.5 μL Dream Taq Master Mix (Thermo Fisher Scientific, Schwerte, Germany), 1 μL of each primer (concentration 10 μM, NS1 and NS2 for the 18S rRNA gene; ITS1 and ITS4 for the ITS1-5.8S rRNA gene-ITS2), and 9.5 μL of DNA-free water (Thermo Fisher Scientific, Schwerte, Germany)]. PCR was performed in a T-1 thermocycler (Biometra, Göttingen, Germany) using the following amplification conditions for the ITS region: denaturation 94°C for 8 min, 35 cycles of denaturation 94°C for 30 s annealing 48°C for 45 s, elongation 72°C for 3 min, final elongation 72°C for 10 min, cooling 4°C. For amplification of the 18S rRNA gene, the following conditions were applied: initial denaturation 94°C for 85 s, 30 cycles of denaturation 94°C for 35, annealing 55°C for 55 s, elongation 72°C for 3 min, final elongation 72°C for 10 min (White et al., 1990). PCR products were checked for correct length by gel electrophoresis (applying 120 V for 20 min) using SYBR Safe (Thermo Fisher Scientific, Eugene, OR, United States) pre-stained 1% TBE agarose gels, and GeneRuler 1 kb DNA Ladder (Thermo Scientific GeneRuler^TM^, Schwerte, Germany) as fragment length control. Sanger sequencing of PCR products was performed at the Institute of Clinical Molecular Biology (IKMB) of Kiel University. Sequences were analyzed and trimmed with Chromas Pro version 1.33 (Technelysium Pty Ltd., South Brisbane, Australia) and compared to the NCBI nucleotide database^2^ using the BLASTn algorithm (Altschul et al., 1990). Nucleotide sequences obtained in this study were submitted to GenBank. Assigned accession numbers are displayed in **Supplementary Table S1**.

### Cultivation

#### Selection of Media

Pre-selection of media for the co-cultivation experiment was done by investigating the growth of phytopathogenic bacteria and fungi (*M. oryzae*, *B. cinerea*, *S. tritici*, *R. solanacearum*, *P. syringae*, and *E. amylovora*) on 11 agar media; GPY medium, WSP medium, PCA medium, HS medium, SA medium (peptone 10 g, glucose 20 g, agar 15 g, and dH_2_O *ad* 1 L: pH = 5.6), MA medium (malt extract 10 g, agar 15 g, and dH_2_O *ad* 1 L), MYA medium (malt extract 10 g, yeast extract 4 g, glucose 4 g, agar 15 g, and dH_2_O *ad* 1 L), PDA medium (potato starch 4 g, glucose monohydrate 4 g, agar 15 g, and dH_2_O *ad* 1 L), M1 agar medium (peptone 5 g, peptone from meat 3 g, agar 15 g, and dH_2_O *ad* 1 L), and Tryptic Soy Broth (TSB) agar medium (TSB 12 g, NaCl 5 g, agar 15 g, and dH_2_O *ad* 1 L) and WSP^∗^ (WSP augmented with streptomycin and tetracycline) (**Table 1**). No growth or poor growth was thus taken as an exclusion criterion to minimize the number of media. SA, PDA, and TSB media supported the growth of all phytopathogens (**Table 1**). We exclusively used solid media with agar as a gelling agent to enable direct visualization of the interaction between marine-adapted isolates and phytopathogens during co-cultivation.

**Table 1:** Initial growth assessment studies with the phytopathogens (fungi and bacteria) on 11 media for optimal media selection.

Phytopathogen	PCA	M1	WSP^∗^	GPY	SA	MYA	PDA	MA	TSB	HS	WSP
*S. tritici*	+	+	+	Poor	+	+	+	+	+	+	+
*B. cinerea*	+	Poor	+	Poor	+	Poor	+	+	+	+	+
*M. oryzae*	+	+	+	+	+	+	+	+	+	+	+
*E. amylovora*	–	+	–	–	+	Poor	+	–	+	–	+
*P. syringae*	–	+	+	–	+	+	+	+	+	–	+
*R. solanacearum*	–	+	–	–	+	+	+	Poor	+	–	–


*+ represents growth; – represents no growth; poor represents poor growth of one to three colonies. “No growth” and poor growth were used as exclusion criteria. SA, PDA, and TSB were the media that supported the growth of all phytopathogens. ^∗^ Antibiotics added.*

#### Selection of Phytopathogenic Challengers for Co-cultivation

To determine which phytopathogenic fungi and bacteria serve as co-cultivation partners, a pilot experiment was performed using three selected fungal isolates from the Windebyer Noor, namely two representatives of the class Sordariomycetes (*Acremonium* sp. and *Microdochium* sp.) and one representative of the class Dothideomycetes (*Cladosporium* sp.). These fungi were challenged by phytopathogenic fungi and bacteria (*M. oryzae*, *B. cinerea*, *S. tritici*, *R. solanacearum*, *P. syringae*, and *E. amylovora*). Single cultures of the three fungal isolates, the phytopathogens as well as their respective co-cultures were prepared in SA, PDA, and TSB media for extraction and chemical profiling studies.

#### Co-cultivation

Pre-cultures of sampled fungi were inoculated on PDA medium and grown for 14 days at 22°C in the dark. Pre-cultures of the plant pathogens were also prepared in their respective optimal media. The pre-cultures of the pathogenic fungi *M. oryzae*, *B. cinerea*, and *S. tritici* were grown on PDA medium, while the pre-cultures of the phytopathogenic bacteria were prepared in broth media. Thus, *R. solanacearum* was inoculated in M1 broth (peptone 5 g, peptone from meat 3 g, and dH_2_O *ad* 1 L); while *P. syringae* and *E. amylovora* in TSB broth (TSB 12 g, NaCl 5 g, and dH_2_O *ad* 1 L) followed by incubation for 24 h at 28°C in a shaking incubator (160 rpm).

For co-cultivation with fungal phytopathogens, a 1 cm^2^ piece from each co-cultivation partner was streaked and placed in ca. 5 cm distance from each other on a new agar plate (9 cm in diameter). For co-cultivation with bacterial phytopathogens, 50 μL of pre-cultures were pipetted on one side of the respective agar plates and spread using a sterile inoculation loop (at a distance of 5 cm from the fungal isolate). Co-cultures were incubated at 22°C for 21 days in the dark. In parallel, mono-cultures of each strain used for co-cultivation were prepared and cultivated at the same conditions for comparison. Microscopic images of selected co-cultures were acquired using a Zeiss Discovery V8 stereomicroscope equipped with a Zeiss Axiocam MRm (Rev 2, Carl Zeiss AG, Oberkochen, Germany).

### Extraction

The entire solid cultures were sliced into small pieces with a flat spatula and transferred into 250 mL glass flasks. 100 mL of ethyl acetate (EtOAc, PESTINORM, VWR Chemicals, Leuven, Belgium) was added and the mixture was homogenized with an Ultra-Turrax at 19,000 rpm before filtering through a folded cellulose membrane filter (type 113P, Rotilabo^®^ROTH, Karlsruhe). The filtrate was collected into a glass separating funnel and washed with 100 mL of Milli-Q^®^water (Arium^®^Lab water systems, Sartorius) to remove salts and water-soluble compounds. A total of 100 mL of crude EtOAc extract was collected and evaporated to dryness by a rotary evaporator (150 rpm at 40°C). Dried extracts were re-dissolved in 3 mL of UPLC/MS grade methanol (MeOH) and pipetted into a pre-weighed 4 mL-amber glass vial through a 13 mm syringe filter w/0.2 μm PTFE membrane (VWR International, Darmstadt, Germany). After drying the extracts under a nitrogen atmosphere, vials were weighed to determine the extract amount. The extracts were stored at -20°C.

### UPLC–QToF–MS/MS analysis

For LC–MS analysis, the extracts were dissolved in UPLC/MS grade MeOH at a concentration of 0.1 mg/mL and 180 μL was transferred into a 1.5 mL HPLC vial equipped with a 200 μL inlet vial. Analyses were performed on an Acquity UPLC I-Class System coupled to a Xevo G2-XS QToF Mass Spectrometer (Waters, Milford, MA, United States).

Samples were injected and chromatographically separated on an Acquity UPLC HSS T3 column (High Strength Silica C_18_, 1.8 μm, 100 × 2.1 mm, Waters^®^, Milford, MA, United States) at a temperature of 40°C with an injection volume of 2 μL. Separation was achieved with a binary LC solvent system controlled by MassLynx^®^version 4.1. Mobile phase A consisted of 99.9% MilliQ^®^-water/0.1% formic acid (UPLC/MS grade) and mobile phase B contained 99.9% acetonitrile (ACN, UPLC/MS grade, Biosolve BV, Dieuze, France)/0.1% formic acid, which were pumped at a rate of 0.6 mL/min with the linear gradient starting with 99% A from minute 0–11.5 followed by 0% A for 1 min (11.5–12.5), and a column reconditioning phase until minute 15.

Mass spectrometry (MS) data were acquired simultaneously with an electrospray ionization source in the positive mode at a mass range of *m/z* 50–1600 Da. The following parameters were used for data acquisition: capillary voltage of 0.8 kV, cone and desolvation gas flow of 50 and 1200 L/h respectively, source temperature of 150°C and desolvation temperature of 550°C with sampling cone and source offset at 40 and 80, respectively. The MS/MS fragmentation was achieved with ramp collision energy (CE): Low CE from 6 to 60 eV and a high CE of 9 to 80 eV. As controls, solvent (MeOH) and non-inoculated medium were injected under the same conditions.

### Molecular Networking and Metabolomics Analysis

Raw data obtained from the LC–MS/MS system were converted to mzXML format using the ProteoWizard tool msconvert (version 3.0.10051, Vanderbilt University, United States) (Chambers et al., 2012). All mzXML data were uploaded to the Global Natural Products Social (GNPS) MN webserver^3^ and analyzed using the MN workflow published by Wang et al. (2016). The data were filtered by removing all MS/MS peaks within ±17 Da of the precursor *m/z*. The MS/MS spectra were window filtered by choosing only the top six peaks in the ±50 Da window throughout the spectrum. To create consensus spectra, data were clustered by applying a parent mass tolerance of 0.02 Da and an MS/MS fragment ion tolerance of 0.02 Da. Further, consensus spectra containing less than 2 spectra were discarded. A network was then created where edges were filtered to have a cosine score above 0.7 and more than six matched peaks. Further edges between two nodes were kept in the network only if each of the nodes appeared in each other’s respective top 10 most similar nodes. The spectra in the network were then searched against GNPS spectral libraries. The library spectra were filtered in the same manner as the input data. All matches kept between network spectra and library spectra were required to have a score above 0.7 and at least six matched peaks (Wang et al., 2016). The MS/MS spectra were then searched against the ISDB (Allard et al., 2016) which uses the fragmentation tool CFM-ID (Allen et al., 2014) and a spectral library from the UNPD (ISDB–UNPD). Parameters used for the fragmentation and spectral searches included a tolerance of 0.005, score threshold 0.2, and top k results 5.

The output of the molecular networks and Euler diagrams were visualized using Cytoscape version 3.6.1^4^ (Shannon et al., 2003) and displayed using the settings “preferred layout” with “directed” style. The nodes (compounds) originating from media and solvent control (MeOH) were excluded from the original network to enable visualization of metabolites deriving from mono- and co-cultures. Principal component analysis and S-plots were generated using Progenesis QI (Version 3.0.1, Waters^®^, Milford, MA, United States).

Manual annotation of peak ions was performed by searching their predicted molecular formulae against databases such as Dictionary of Natural Product (DNP), MarinLit, Reaxys, and SciFinder. Peak ion intensities were also checked for quantitative comparison between mono- and co-cultures. Compound hits were validated based on their biological source and fragmentation patterns using the CFM-ID web server (Allen et al., 2014).

### Bioactivity

Bioactivity screening of mono- and co-culture extracts, using the broth dilution technique in 96-well microplates, was performed against a panel of six phytopathogens, i.e., four bacteria (*X. campestris*—causative agent of black rot in crucifers, *R. solanacearum—*causative agent of potato brown rot, *P. syringae*—causative agent of cankers and blights in a variety of crop species, and *E. amylovora*—causative agent of fire blight disease in pome fruits), a fungus (*M. oryzae*—causative agent of rice blast disease), and an oomycete (*P. infestans—*causative agent of potato blight disease). Extracts were dissolved in MeOH and diluted in broth (depending on test strain) to a final assay concentration of 200 μg/mL. The final concentration of MeOH in the microplate wells was 0.5%. Phytopathogenic fungi were prepared for bioactivity assays in the following ways: 5 mL of broth (depending on test strain) was poured on 2 weeks old solid cultures and the surface was gently agitated to suspend the spores in the broth. The broth was then transferred to a Falcon^TM^ 50 mL conical centrifuge tube through a sterile gauze to eliminate agar pieces while the spores passed through the gauze. Spores were counted using a Neubauer counting chamber (0.100 mm depth BLAUBRAND, Germany) and a light microscope (Zeiss Axioskop 40, Carl Zeiss AG, Oberkochen, Germany). To achieve a fungal inoculum concentration of 10^4^ spores/mL, the suspension was diluted with media broth accordingly. Bacterial phytopathogens were prepared for the bioactivity assay by incubating the pre-cultures under shaking conditions (160 rpm) at 28°C for 24 h, followed by determination of the optical density (OD) at 600 nm. Inocula diluted to an OD of 0.03 were added to the extracts in the microplate in final volumes of 200 μL. Negative controls included inoculum without extract and inoculum with 0.5% MeOH. The following controls (sterile media and bioactive positive controls) are dependent on test strains and were included on each test plate: for *X. campestris*, *P. syringae*, and *E. amylovora*, TSB and chloramphenicol; for *R. solanacearum*, M1 broth and tetracycline; and for *M. oryzae*, SA broth and nystatin; for *P. infestans*, PB (Pea Broth: pea 150 g, glucose 5 g, thiamine HCl 0.1 mg, and dH_2_O *ad* 1 L) and cycloheximide. Absorbance at 600 nm before and after incubation was recorded using an Infinite M200 reader (TECAN Deutschland GmbH, Crailsheim, Germany). Assay incubation time for bacterial phytopathogens was 9 h (shaking at 200 rpm) at 28°C, whereas the oomycete and the fungal phytopathogen were incubated for 96 h (shaking at 200 rpm) at 22°C. The IC_50_ values were determined by GraphPad Prism 7 software using concentrations ranging from 0.29 to 200 μg/mL. All cultures were grown in triplicates.

## Results

### Characterization and Identification of Fungal Isolates

**FIGURE 1:**
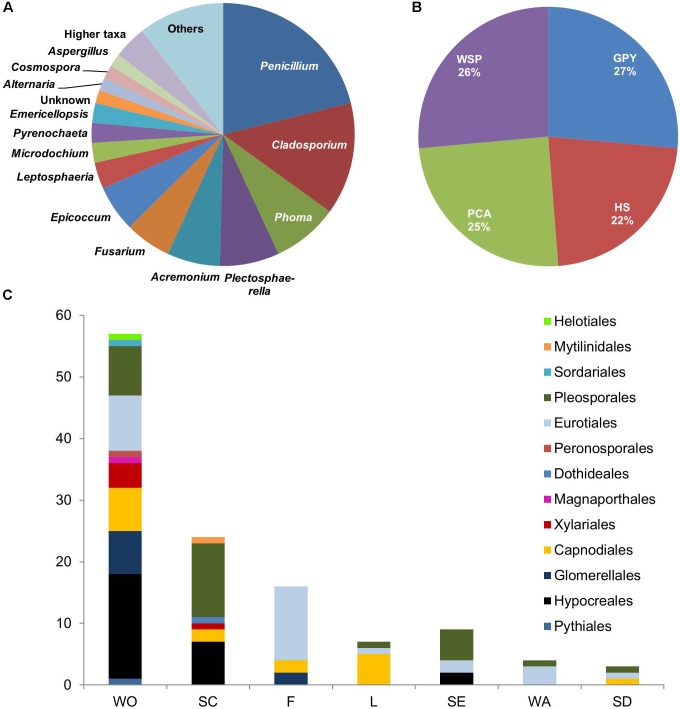
Diversity and distribution of 123 isolates obtained from sampling in Windebyer Noor by **(A)** Fungal genera. **(B)** Isolation media: Isolation was performed using four different solid media, i.e., PCA medium, HS medium, GPY medium, and WSP medium. **(C)** Isolation source (fungal orders). WO, twigs; SC, wood scrapings of a driftwood; SD, seeds; WA, water; F, foam; L, leaves; SE, sediment.

Our isolation efforts using samples of water, sediment, seafoam, and plant materials (seeds, twigs, leaves, and wood scrapings) yielded 123 fungal strains isolated on four different media. Most fungal isolates showed a high similarity (>97%, BLASTn) to GenBank database entries, but 10 isolates (affiliated to the genera *Acremonium*, *Cladosporium*, *Phoma*, *Emericellopsis*, *Pseudohalonectria*, and *Phaeosphaeria*; **Supplementary Table 1**) showed considerably lower similarities (92–96%) to the closest relative GenBank sequence. Apart from two isolates (**Supplementary Table 1**), which were identified as representatives of the Oomycetes, namely the genera *Pythium* and *Phytophthora*, all isolates were affiliated to the division Ascomycota within the fungal subkingdom Dikarya. Most isolates were assigned to the genera *Penicillium* (26 isolates) and *Cladosporium* (17 isolates) (**Figure 1A**). Multiple isolates were also affiliated to the genera *Phoma* (10 isolates), *Plectosphaerella* (9 isolates), *Acremonium* (10 isolates), *Epicoccum* (7 isolates), and *Fusarium* (7 isolates). One isolate each was obtained from the following genera (listed as “others” in **Figure 1A**): *Aureobasidium*, *Sarocladium*, *Cadophora*, *Chordomyces*, *Gibellulopsis*, *Phialemonium*, *Plenodomus*, *Pseudohalonectria*, *Phaeosphaeria*, and *Hypoxylon.* Five isolates could not be identified to the genus level but only to a higher taxonomic level (orders Helotiales, Hypocreales, Pleosporales, and family Lindgomycetaceae) and are represented as “higher taxa” in **Figure 1A**.

In total, 30 different fungal genera were obtained by the isolation approach. All media proved to be equally suitable for isolation of fungal strains since each medium contributed about 25% to the total number of isolates (**Figure 1B**). At the genus level, *Penicillium* was dominant in three media (PCA, Hastings, and GPY media) whereas *Acremonium*, *Epicoccum*, and *Cladosporium* showed relatively high abundance in the WSP medium (**Supplementary Figures 2A–D**). As shown in **Figure 1C**, more than 70% of isolates were derived from plant materials, such as twigs (WO: 55 isolates), wood scrapings (SC: 25 isolates), seeds (SD: 3 isolates), or leaves (L: 7 isolates). The sediment sample contributed to about 7% of the total isolates. Interestingly, 15% of isolates were derived from the seafoam and 5% were obtained from the water sample (**Supplementary Figures 2E–G**).

### Fungal Strain Selection and Establishment of Optimal Co-cultivation Conditions

#### Selection of Media and Phytopathogenic Challengers for Co-cultivation

The initial media selection experiments showed the suitability of three media (SA, PDA, and TSB) for the growth of all the phytopathogens (**Table 1**). However, during the screening for the phytopathogenic challengers, two media, namely PDA and SA, were chosen for all subsequent cultivation experiments, because they exhibited (i) decent growth of phytopathogens and co-cultures, (ii) increased chemical diversity (as measured by the number of new peaks detected in HPLC chromatograms) in co-culture, and (iii) competitive or antagonistic interactions visualized directly on agar plates for all co-culture pairs. *M. oryzae*, *B. cinerea*, *R. solanacearum*, and *P. syringae* as phytopathogenic challengers showed most distinct changes in points (ii)–(iii) and thus led to the selection of these four phytopathogens for further co-cultivation experiments.

#### Selection of Marine-Adapted Fungi for Co-cultivation Experiments

In total, 21 genetically diverse strains out of 123 marine-adapted isolates were selected for co-cultivation (**Table 2**) with the four pre-selected phytopathogens in both pre-selected media (SA and PDA) yielding in total 218 extracts. 168 extracts derived from co-cultures and 50 from mono-cultures of marine-adapted isolates and phytopathogens, which were further subjected to UPLC–MS/MS-based metabolomics, dereplication, and bioactivity assays against a panel of six plant pathogens. The experimental workflow is shown in **Supplementary Figure 3**.

**Table 2:** List of 21 genetically diverse strains selected out of 123 fungal isolates, co-cultured with four phytopathogens in two solid media (SA and PDA), tested for anti-phytopathogenic activity at 200 μg/mL concentration and chemically profiled.

No.	Identity	Source	Macroscopic change in co-culture	Increased chemo-diversity	Bioactivity (>70%) against ≥3 phytopathogens in co-cultures	Selected co-culture partner with activity (>70%) against ≥3 phytopathogens
1	*Aspergillus* sp.	w	z	*^∗^*		
2	*Cadophora* sp.	w	c	^∗^		
3	***Emericellopsis* sp.**	w	d	^∗^	*^∗^*^∗^	M.o., P.s. (SA)
4	***Hypoxylon* sp.**	w	z	^∗^	*^∗∗^*	M.o. (PDA)
5	*Fusarium* sp.	w	c	^∗^		
6	***Alternaria* sp.**	w	o	^∗^	*^∗∗^*	P.s. (PDA)
7	*Leptosphaeria* sp.	w	c	^∗^		
8	Hypocreales sp.	w	d	^∗^		
9	*Epicoccum* sp.	w	c	^∗^		
10	***Acremonium* sp.**	w	c	^∗^	*^∗∗^*	M.o., P.s., B.c. (PDA)
11	***Cosmospora* sp.**	w	z	^∗^	*^∗∗^*	M.o., P.s. (PDA)
12	*Aureobasidium* sp.	s	c	^∗^		
13	*Pyrenochaeta* sp.	w	c	^∗^		
14	*Cladosporium* sp.	w	c	^∗^		
15	Pleosporales sp.	w	d	^∗^		
16	*Pseudohalonectria* sp.	w	c	^∗^		
17	Dothideomycetes sp.	w	c	^∗^		
18	Pleosporales sp.	w	z	^∗^		
19	*Acremonium* sp.	w	c	^∗^		
20	*Phialemonium* sp.	w	c	^∗^		
21	*Plenodomus* sp.	s	d	^∗^		


*Sources: w, wood; s, sediment. Interaction type: z, zone inhibition; d, distance inhibition; o, overgrowth; c, contact inhibition. **^∗^**Presence of increased chemical diversity. ^∗∗^Bioactivity (>70%) against ≥3 phytopathogens in co-cultures. Selected strains used for the nine co-cultures are marked in bold. Co-culture partners: M.o., M. oryzae; P.s., P. syringae; B.c., B. cinerea*.

The selected marine-adapted fungal isolates (21) and their co-cultures exhibited different macroscopic interactions, and furthermore, they often showed increased chemical diversity (assessed by peak numbers in UPLC–QToF–MS chromatograms) and anti-phytopathogenic bioactivities. As shown in **Table 2**, extracts of nine co-cultures showed bioactivities ≥70% at a concentration of 200 μg/mL against at least three phytopathogenic test strains. The nine co-cultures and their corresponding mono-cultures were submitted for an in-depth comparative UPLC–MS/MS-based untargeted metabolomics study. Their *in vitro* activity (IC_50_ values) against the panel of six phytopathogens was also determined. Based on bioactivity data (**Table 2**), the remaining marine-adapted fungi and the phytopathogen *R. solanacearum* as a co-culturing partner were excluded from further studies.

**FIGURE 2:**
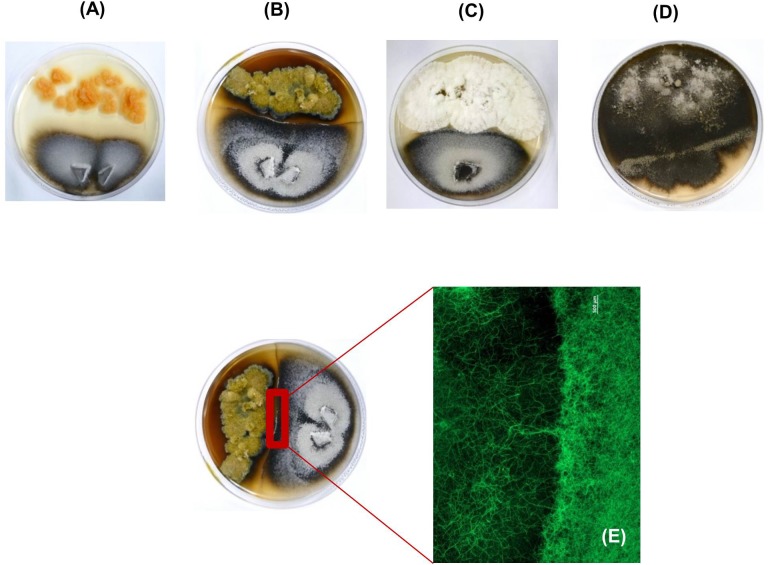
Macroscopic images of the co-cultures of **(A)** marine-adapted *Emericellopsis* sp. (top of Petri dish) with the phytopathogenic fungus *M. oryzae* (bottom of the Petri dish) showing a distance type of interaction. **(B)** *Cosmospora* sp. (top of the Petri dish) with *M. oryzae* (bottom of the Petri dish) showing a darkened confrontation zone interaction. **(C)** *Acremonium* sp. (top of the Petri dish) with *M. oryzae* (bottom of the Petri dish) displaying a contact type of inhibition. **(D)** *Alternaria* sp. (top of the Petri dish) with *P. syringae* (bottom of the Petri dish) showing an overgrowth type interaction. **(E)** Stereomicroscopic picture of the confrontation zone between *Cosmospora* sp. (left) and *M. oryzae* (right) with a scale bar representing 500 μm. It is observed that the hyphae of the phytopathogen *M. oryzae* do not grow further into the confrontation zone. The zone is however dominated by the hyphae of *Cosmospora* sp.

Different interaction types (as previously described by Bertrand et al., 2014) were observed on the agar plates of the nine selected co-cultures after 21 days of incubation: two co-cultures were characterized by distance inhibition (gap between strains, **Figure 2A**), three co-cultures exhibited a zone of confrontation (distinct line between strains, **Figure 2B**), whereas three others showed contact inhibition (strains seem to exist mutually, touching each other, depicted in **Figure 2C**) and one co-culture showed overgrowth (a strain overgrows the other, **Figure 2D**). Pictures of all cultures are shown in **Supplementary Figure 4**. UPLC–MS/MS-based MN (**Supplementary Figures 5–13**, discussed in the section “dereplication and annotation of molecular clusters”) showed induction of new compounds, which were only produced in co-cultures but not in mono-cultures irrespective of the macroscopically observed interaction type.

Especially the phytopathogenic fungus *M. oryzae* was characterized by high growth rates, resulting in the formation of a lawn covering the entire agar plate within 7 days (**Supplementary Figure 4B**). However, the presence of the marine-adapted fungus *Cosmospora* sp. isolated from driftwood scrapings prevented the spread of *M. oryzae* over the entire agar plate. Instead, a macroscopically visible, dark-brown boundary was formed at the confrontation zone between the two partners. Microscopic analysis of the confrontation zone revealed the exclusive presence of *Cosmospora* sp. mycelia and no phytopathogen-derived hyphae were observed in this zone (**Figure 2E**). Hence, *Cosmospora* sp. obviously inhibits the growth of one of the most devastating fungal phytopathogens. This finding made the co-culture of *Cosmospora* sp. with *M*. *oryzae* an ideal candidate for further analysis. For this culture, extraction and chemical profiling of the confrontation zone were performed in order to evaluate if macroscopic observations translate into enhanced chemical diversity.

### Bioactivity Assays

**Table 3** displays the IC_50_ values determined for the extracts of all five marine-adapted single fungal cultures as well as nine co-cultures against a panel comprised of six phytopathogens. Overall the mono-culture of the marine fungus *Cosmospora* sp. exhibited the highest activity against all phytopathogens, except for *E. amylovora*. *X. campestris*, *M. oryzae*, and *P. infestans* were the most susceptible pathogens, while *E. amylovora* was insensitive to any extract even at the highest test concentrations (200 μg/mL).

**Table 3:** Anti-phytopathogenic activity of the nine co-culture extracts and their corresponding mono-cultures.

Strain name	Co-culture partner	Medium	IC_50_ values against economically relevant phytopathogens (μg/mL)					
			X.c.	E.a.	P.s.	R.s.	M.o.	P.i.
*Emericellopsis* sp.	None	SA	56	>200	>200	>200	76	67
	*M. oryzae*	SA	54	>200	>200	>200	73	57
	*P. syringae*	SA	152	>200	>200	>200	150	150
*Hypoxylon* sp.	None	PDA	>200	>200	>200	150	179	153
	*M. oryzae*	PDA	>200	>200	>200	180	128	157
*Alternaria* sp.	None	PDA	32	>200	>200	>200	127	117
	*P. syringae*	PDA	28	>200	>200	>200	119	115
*Acremonium* sp.	None	PDA	44	>200	>200	>200	23	52
	*M. oryzae*	PDA	52	>200	>200	>200	33	51
	*B. cinerea*	PDA	56	>200	>200	>200	112	150
	*P. syringae*	PDA	69	>200	>200	>200	110	115
*Cosmospora* sp.	None	PDA	6	>200	78	57	8	5
	*M. oryzae*	PDA	15	>200	150	131	29	6
	*P. syringae*	PDA	6	>200	150	89	12	63
PDA (MC)			>200	>200	>200	>200	>200	>200
SA (MC)			>200	>200	>200	>200	>200	>200
K+			2.46	2.32	2.32	0.74	0.44	0.24
Solvent control			>200	>200	>200	>200	>200	>200


*Mean values are based on triplicate measurements. Phytopathogens: X.c., X. campestris; E.a., E. amylovora; P.s., P. syringae; R.s., R. solanacearum; M.o., M. oryzae; P.i., P. infestans. Methanol was used as a solvent control. Positive control (K+) for X.c., E.a., and P.s. was 10 μM chloramphenicol; for R.s. 10 μM tetracycline, for M.o. 10 μM nystatin, for P.i. 10 μM cycloheximide; MC, represents agar media controls.*

The single culture *Cosmospora* sp. extract showed remarkable activity against *X. campestris*, *M. oryzae*, and *P. infestans* (IC_50_ values 6, 8, and 5 μg/mL, respectively) and lower inhibitory potential against *P. syringae* and *R. solanacearum* (IC_50_ values 78 and 57 μg/mL). The co-culture extract of *Cosmospora* sp. with *M. oryzae* retained the identical activity against *P. infestans* (IC_50_ 6 μg/mL) but displayed significantly diminished potency against other phytopathogens (**Table 3**). In turn, the co-culture extract of *Cosmospora* sp. with *P. syringae* showed comparable activities to that of *Cosmospora* mono-culture against *X. campestris* and *M. oryzae* (IC_50_ values 6 and 12 μg/mL, respectively), while the remaining pathogens were significantly less susceptible. A 10-fold reduction in bioactivity observed against *P. infestans* (IC_50_ 63 μg/mL) was notable (**Table 3**).

The *Acremonium* sp. mono-culture extract was moderately active against *M. oryzae*, *X. campestris*, and *P. infestans* (IC_50_ values from 23 to 52 μg/mL, respectively). The potency of the *Acremonium* sp. and *M. oryzae* co-culture extract was indifferent to that of the mono-culture. The co-culture extract of *Acremonium* sp. with *B. cinerea* or with *P. syringae* exhibited identical activities against the same three pathogens. Their inhibitory activity was moderate against *X. campestris* but much weaker against *M. oryzae* and *P. syringae* in comparison to the *Acremonium* mono-culture (**Table 3**).

The single, as well as the co-culture extract of *Emericellopsis* sp. with *M. oryzae* moderately inhibited the growth of *X. campestris*, *M. oryzae*, and *P. infestans* (IC_50_ values 54–76 μg/mL). The co-culture of *Emericellopsis* sp. with *P. syringae*, however, showed poor activity (IC_50_ values 150 μg/mL) toward the same pathogen panel (**Table 3**).

*Alternaria* sp. single culture extract exerted moderate inhibition against *X. campestris* (IC_50_ 32 μg/mL) and little activity against *M. oryzae* and *P. infestans* (IC_50_ values 127 and 117 μg/mL, respectively) (**Table 3**). The confrontation of this fungus with the pathogenic bacterium *P. syringae* did not lead to any change in the bioactivity profile of the mono-culture (**Table 3**).

Finally, the EtOAc extract of the *Hypoxylon* sp. mono-culture demonstrated slight activity against *R. solanacearum*, *M. oryzae*, and *P. infestans* (IC_50_ values 150–179 μg/mL). The co-cultivation of *Hypoxylon* sp. with *M. oryzae* failed to result in any significant change in bioactivity against the same pathogens (IC_50_ values 128–180 μg/mL; **Table 3**).

### Molecular Network-Based Dereplication of Nine Co-cultures and Mono-cultures

**FIGURE 3:**
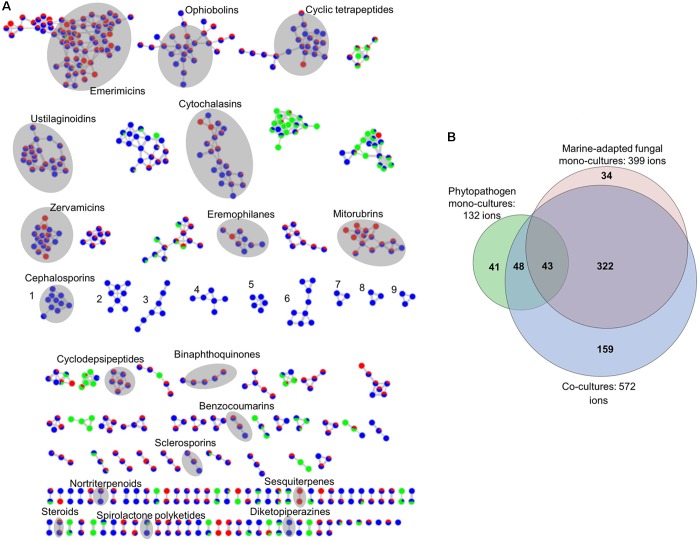
**(A)** Global molecular network of the nine co-cultures of marine-adapted fungi and phytopathogens and their respective mono-cultures. Red nodes, mono-cultures from marine fungal isolates; green nodes, mono-cultures from phytopathogens; blue nodes, co-cultures. The global molecular network highlights that co-cultivation increased the chemical space in comparison to mono-cultures and therefore contains data derived from five different marine-adapted fungal isolates cultivated in two different media with three different phytopathogens. Nine clusters induced only in co-cultures (clusters numbered 1–9 in blue only nodes). Separate molecular networks for all nine co-cultures and the respective mono-cultures are given in **Supplementary Figures 5–13**. **(B)** Global Euler diagram based on the global molecular network (MN) in **(A)**. Red, ions from mono-cultures of marine-adapted fungal isolates obtained during this study; green, ions obtained from mono-cultures of phytopathogens; blue, ions obtained from co-cultures. It shows unique ions for mono-cultures of phytopathogens (41), marine-derived fungi (34), and co-cultures (159). Ions observed in all culture types were 43 while 48 ions were common to the phytopathogens and the co-cultures. Co-cultures also revealed 322 ions common to the marine-adapted fungi. Ions originating from the blank media and solvents were removed prior to MN and Euler diagram analysis.

For analyzing the chemical inventory of mono- and co-cultures comparatively, a global molecular network based on UPLC–HRMS/MS data was generated using the GNPS platform and complemented with ISDB–UNPD (Allard et al., 2016) and manual dereplication. This approach captured diverse chemical structural classes and analogs into different clusters based on MS^2^ spectral alignment algorithms independent of retention times (*t*_R_), extract type, or cultivation media, thereby rapidly identifying putatively new molecules and new derivatives of known molecules (**Supplementary Table 2** and **Supplementary Figures 5–22**). **Figure 3A** displays the global/composite MN generated from all co-cultures and the corresponding marine-adapted fungal and phytopathogen mono-cultures.

The composite MN of all the nine co-culture extracts consisted of 1370 nodes, which were grouped into 66 clusters (more than 2 nodes per cluster). Some nodes represented adducts, thus, not all network nodes correspond to a single molecule. As shown in **Figure 3A**, some chemical families annotated include, e.g., emerimicins (linear peptides), ophiobolins (sesterterpene phytotoxins), ustilaginoidins (naphtho-γ-pyrone type polyketides), cytochalasins (indole alkaloids), mitorubrins (azaphilone type polyketides), eremophilanes (sesquiterpenes), and sclerosporins (cadinane-type sesquiterpenes). In total, 18 molecular families were annotated. The global MN showed that nine molecular clusters were exclusively induced in the co-cultures (numbered 1–9 in **Figure 3A**). Clusters 1–5 were only expressed in the co-culture of *Emericellopsis* sp. and *P. syringae*, while clusters 6–9 were exclusive to the co-cultures of *Acremonium* sp. Cluster 1 was putatively annotated as cephalosporins (non-ribosomal peptide derivatives). Clusters 2–9 comprised ions ranging from *m/z* 300 to 800 Da and could not be annotated, suggesting their potential novelty. Although our integrated dereplication effort improved annotation of compounds, several ions originating from different culture types (mono- and co-culture) belonging to known and unknown clusters remain unannotated; hence, they could represent putatively new compounds. In addition to the induction of new chemical clusters, we observed altered production levels of some compounds in the co-cultures. For example, the concentration of mitorubrinic acid showed a twofold increase while cytochalasin J was exclusively expressed in the co-culture of *Hypoxylon* sp. with *M*. *oryzae*. This co-culture lacked 3,4-dihydromitorubrinol acetate that was detected in the single culture of *Hypoxylon* sp. The concentration of tenuazonic acid, a tetramic acid type component of the *Alternaria* sp. mono-culture, was considerably increased in its co-culture with *P. syringae*. Nortenuazonic acid was only expressed in the mono-culture of *M.* *oryzae*. A tentatively identified compound, ergone, which was detected in the mono-culture of *B. cinerea* had a considerably low concentration in its co-culture. The single culture extract of *Acremonium* sp. contained a putative hit cyclocitrinol, whereas all *Acremonium* co-cultures were deficient in this compound. A list of all annotated compounds can be found in **Supplementary Table 2**. The global MN-derived Euler diagram (**Figure 3B**) displays the node (ion) distribution in cultures. Single phytopathogenic cultures contained 132 ions while the mono-cultures of 5 marine fungi collectively displayed 399 ions. The nine co-cultures showed in total 572 ions, 43 of which were produced by all mono-cultures while 48 were common to the phytopathogen mono-cultures. The majority of ions (322) in co-cultures were produced only by the mono-cultures of the marine-adapted fungi. Most importantly, 159 ions were exclusive to the co-cultures. This indicates that BGCs were activated as a reaction to the biotic challenge triggered by the phytopathogenic partner, thus confirming our hypothesis. Conversely, marine-adapted fungal isolates lacked 34 ions and the phytopathogens lacked 41 ions in co-cultures. Although this result indicates the presence of negative interactions, i.e., suppressive effects, this is lesser in comparison to 159 nodes specifically induced by co-cultivation.

**FIGURE 4:**
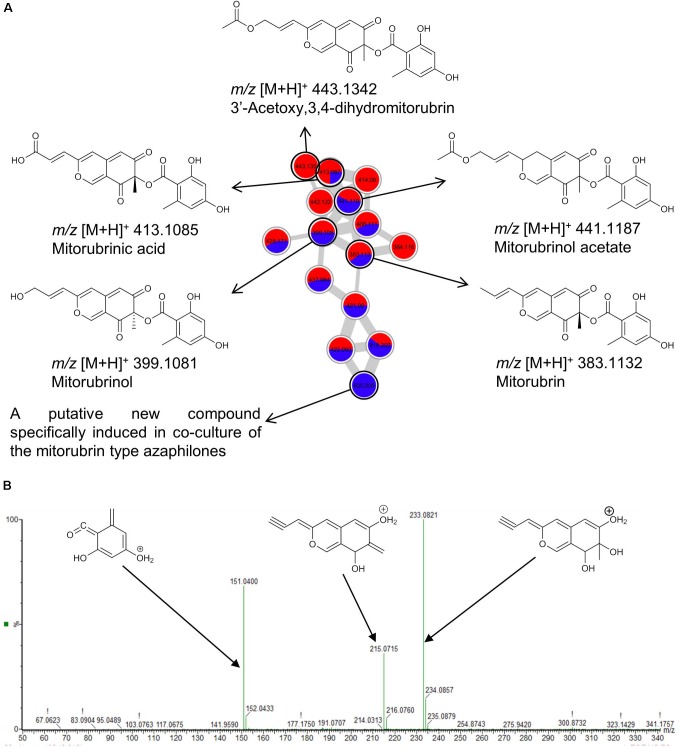
**(A)** Molecular network of the molecular family of mitorubrins extracted from the MN of the *Hypoxylon* sp. and *M. oryzae* co-culture. Blue, ions found only in co-culture extracts; Red, ions detected in mono-cultures of marine-adapted fungal isolates. **(B)** Annotated MS/MS spectrum of mitorubrin (*m/z* [M+H]^+^ 383.1132) acquired by UPLC–QToF–MS/MS in positive mode.

Co-cultivation also increased the size of several molecular families, i.e., the biosynthesis of new derivatives of known clusters by one or more nodes. This is exemplified in **Figure 4A** by the mitorubrin (azaphilone class of polyketides) cluster annotated in the MN of *Hypoxylon* sp. and *M. oryzae* cultures. Mitorubrin was annotated to one ion in this cluster (*m/z* [M+H]^+^ 383.1132, C_21_H_19_O_7_) and its identity was confirmed by fragmentation pattern analysis (**Figure 4B**). The smallest fragment ion *m/z* [M+H]^+^ 151.0400 (C_8_H_7_O_3_) corresponds to dehydrated orsellinic acid. The remaining fragment ions at *m/z* [M+H]^+^ 233.0821 (C_13_H_13_O_4_) and *m/z* [M+H]^+^ 215.0715 (C_13_H_11_O_3_) represent the bicyclic portion of the molecule and its dehydroxylated derivative, respectively (**Figure 4B**). The mitorubrin cluster (**Figure 4A**) showed nodes with a high spectral similarity score (>0.7) corresponding to highly similar MS/MS fragmentation patterns. Immediately annotated nodes corresponded to the hydroxyl- (*m/z* [M+H]^+^ 399.1081, C_21_H_19_O_8_), acetoxy- (*m/z* [M+H]^+^ 441.1187, C_23_H_21_O_9_), and dihydro- (*m/z* [M+H]^+^ 443.1342, C_23_H_23_O_9_) derivatives of mitorubrin, as well as its acid form (mitorubrinic acid, *m/z* [M+H]^+^ 413.1085, C_21_H_17_O_9_) (**Figure 4A**). The fully blue node at the bottom of the MN with *m/z* [M+H]^+^ 820.2050 (C_51_H_32_O_11_, -2.1 ppm error) represents an ion that was detected only in the co-culture of *Hypoxylon* sp. with *M. oryzae*. This node could not be annotated, neither by database search nor by fragmentation pattern, hence may be a new compound.

**FIGURE 5:**
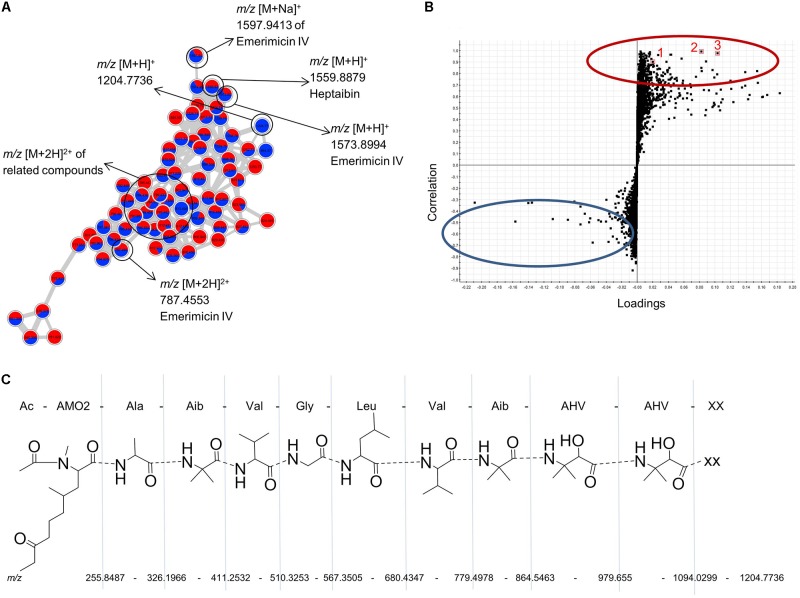
**(A)** Annotated molecular network of the emerimicin cluster based on the UPLC–MS/MS analysis of the mono-cultured *Emericellopsis* sp. and its co-culture with *M. oryzae* (MN shown in **Supplementary Figure 6**). Data were acquired in the positive mode at a mass range of *m/z* 50-1600. **(B)** S-plot from PLS model of extracts of *Emericellopsis* sp., *M. oryzae*, and the respective co-culture. The upper half of the diagram reflects the ions with the highest correlation to unique co-culture ions while the lower half correlates best with ions unique to the mono-cultures. Three ions corresponding to *m/z* [M+H]^+^ 1204.7736 (1), *m/z* [M+H]^+^ 478.2933 (2), and *m/z* [M+H]^+^ 520.3373 (3) are highlighted as examples of co-culture-induced ions which are putatively unknown. **(C)** Annotated fragmentation pattern of a putatively identified unknown peptide of the emerimicin family (*m/z* [M+H]^+^ 1204.7736 based on MS/MS spectrum acquired in positive mode from 50 to 1600 Da). XX, unknown C-terminal amino acid with *m/z* [M+H]^+^ 110.0354 corresponding to a molecular formula of C_4_H_4_N_3_O. AHV, 3-amino-2-hydroxyvaline; Aib, alpha-aminoisobutyric acid; Val, valine; Leu, leucine; Gly, glycine; Ala, alanine; AMO2, 2-amino-*N*,4-dimethyl-8-oxodecanoic acid.

Co-cultivation of the marine-adapted fungus *Emericellopsis* sp. and the phytopathogen *M. oryzae* also enhanced chemical diversity, as exemplified by the cluster analysis of emerimicins. It was the largest cluster in the global MN (**Figure 3A**), comprising approximately 74 nodes (shown in detail in **Figure 5A**). Linear peptides such as emerimicin IV and heptaibin comprising 10–16 amino acids were observed in the mass range of *m/z* 1200–1600 Da as protonated and sodium adducts (**Figure 5A**). As visualized in the network (**Figure 5A**), these peptides are often characterized by double charged ions around *m/z* 700–900 Da. A full MS/MS spectral analysis of emerimicin IV confirmed its annotation with each fragment corresponding to a single amino acid (**Supplementary Figure 23**). An S plot (**Figure 5B**) combining covariance and correlation was generated to visualize induced peaks responsible for the variation between co-culture of *Emericellopsis* sp. and *M. oryzae* and their mono-cultures. Data points (representing marker ions) in the upper right quarter of the S-plot (encircled in red in **Figure 5B**) represent ions that are induced in the co-culture, while marker ions in the lower left quarter (encircled in blue in **Figure 5B**) represent ions exclusive to the mono-cultures. The highlighted ions 1, 2, and 3 in **Figure 5B** represent examples of ions, which were only identified in the co-culture. The marker ion 1 with *m/z* [M+H]^+^ 1204.7736 (C_57_H_98_N_13_O_15_ with 2.2 ppm error) was detected in the emerimicin cluster. Based on its fragmentation pattern, a partial putative structure is proposed for a peptide with a sequence of 11 amino acids. However, the C-terminal amino acid remains unidentified (*m/z* [M+H]^+^ 110.0354, corresponding to C_4_H_4_N_3_O) (shown as XX in **Figure 5C** and **Supplementary Figure 24**). The other two co-culture-specific ions marked in the S-Plot (**Figure 5B**) characterized by *m/z* [M+H]^+^ 478.2933 (2) and *m/z* [M+H]^+^ 520.3373 (3) were located in unannotated clusters of the MN, thus representing potentially novel compounds or new derivatives.

**FIGURE 6:**
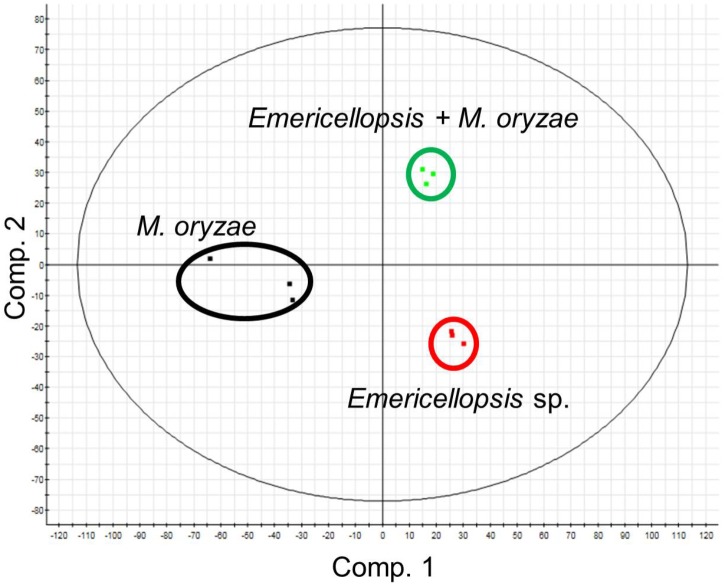
Principal component analysis scores plot of a co-culture of the marine fungal isolate *Emericellopsis* sp. and the plant pathogen *M. oryzae* and their respective mono-culture extracts using PLS-DA model with unit variance scaling. Cultures were grown in triplicates.

Reproducibility of the experiments was assured by measuring three biological replicates per culture. As exemplified by *Emericellopsis* sp., and its co-culture with *M. oryzae* (**Figure 6**), the biological replicates of each culture type (mono-culture of the marine-adapted fungal isolate, phytopathogen mono-culture, co-culture) cluster closely together on the Principal Component Analysis scores plot. Reasonable separation is also observed between the three culture types, i.e., *Emericellopsis* sp. clusters separately from *M. oryzae* and the co-culture, indicating chemical profile differences among the three culture types.

**FIGURE 7:**
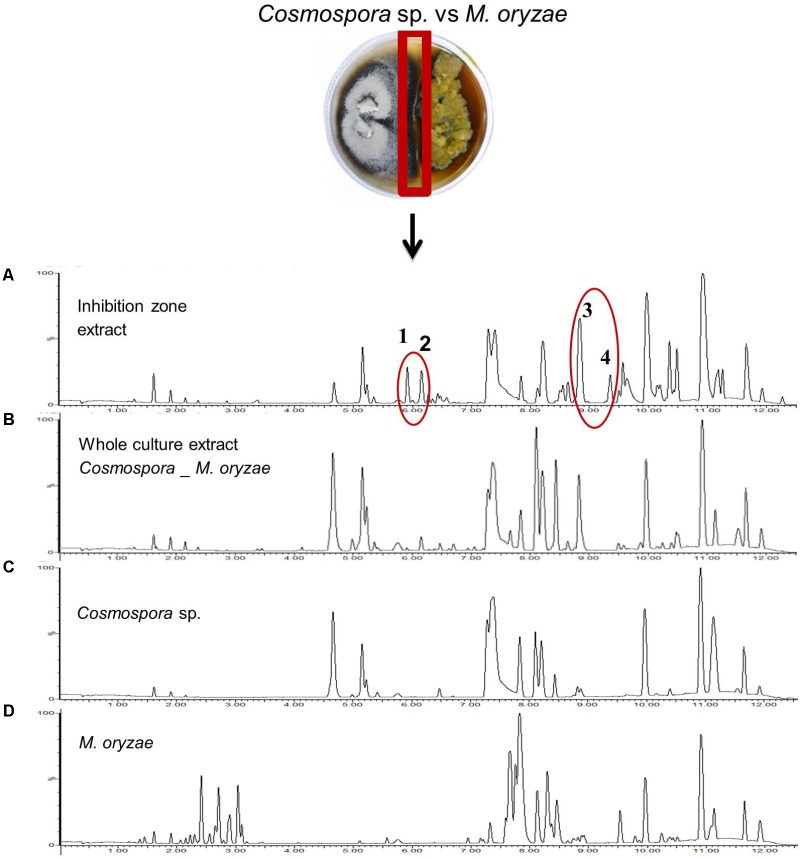
Base peak chromatograms of the extracts obtained from **(A)** the confrontation zone of the co-culture of *Cosmospora* sp. and *M. oryzae*, **(B)** the whole co-culture, and mono-cultures of **(C)** *Cosmospora* sp., and **(D)** *M. oryzae*. Four distinct unannotated peak ions are induced in the co-culture: *m/z* [M+H]^+^ 309.0810, C_14_H_29_O_7_ (1), *m/z* [M+H]^+^ 307.1708, C_14_H_27_O_7_ (2), *m/z* [M+H]^+^ 293.1942, C_14_H_29_O_6_ (3), and *m/z* [M+H]^+^ 329.2318, C_18_H_33_O_5_ (4).

In order to specifically examine the chemistry of the confrontation zone of a co-culture, we selected the co-culture of the marine-adapted fungus *Cosmospora* sp. and the phytopathogenic fungus *M. oryzae*, where we had previously macroscopically observed a color change and microscopic analysis had revealed an inhibition of hyphae spreading of the phytopathogen (**Figure 2B**). The confrontation zone was carefully excised and chemically profiled by UPLC–QTOF–MS/MS. **Figure 7** shows the detailed base peak chromatograms of the confrontation zone extracts aligned with those of the whole co-culture and the mono-cultures of *Cosmospora* sp. and *M. oryzae*. Notably, four significant peak ions, i.e., *m/z* [M+H]^+^ 309.0810 (1), *m/z* [M+H]^+^ 307.1708 (2), *m/z* [M+H]^+^ 293.1942 (3), and *m/z* [M+H]^+^ 329.2318 (4), were detected in the confrontation zone. Compounds 1–4 with the predicted molecular formulae of C_14_H_29_O_7_, C_14_H_27_O_7_, C_14_H_29_O_6_, and C_18_H_33_O_5_, respectively, may represent putatively new fungal microbial natural compounds as all dereplication efforts failed to find reasonable matches. Compounds 1–3 were produced in the co-culture but were substantially overexpressed in the confrontation area. We generated a MN (not shown) to investigate the relation between the four induced peaks in the confrontation zone; only compounds 1–3 clustered closely together. Compound 4, however, was only present in the confrontation area.

## Discussion

### Fungal Isolation

The Windebyer Noor proved to have a considerable culturable fungal diversity yielding a total of 123 isolates, which produced putatively novel bioactive metabolites when cultured alone or together with phytopathogens. In accordance with the fungal diversity observed in brackish water and other marine environments, our study showed a predominance (98%) of Ascomycota (Mohamed and Martiny, 2011; Andreakis et al., 2015) with representatives of the fungal order Pleosporales accounting for the highest number of isolates (**Supplementary Figure 2E**). Pleosporales are commonly found saprophytic fungi in marine habitats (Suetrong et al., 2009) and were accordingly isolated mainly from decaying driftwood scrapings (12 isolates) and twigs (8 isolates). Eurotiales (28 isolates), Hypocreales (26 isolates), and Capnodiales (17 isolates) also showed significant number of representatives (**Supplementary Figure 2E**). These fungal orders are commonly detected in marine habitats, e.g., in the deep Mediterranean Sea sediment and in marine sponges (Imhoff, 2016). We observed major variations in the species composition obtained from different substrates sampled. The surface of decaying plant materials and driftwood freely swimming in the brackish water (WO and SC) accounted for the highest number of isolates contributing >65% of isolates and also comprises the highest fungal culturable diversity (**Figure 1C** and **Supplementary Figure 2**). This was expected since woody substrates represent an important source of carbon and energy for heterotrophic organisms in aquatic ecosystems (Nambiar et al., 2009). Interestingly, our isolation effort also yielded two oomycetes *Pythium* sp. and *Phytophthora* sp., which are known for their notorious plant pathogenicity (Fuechtbauer et al., 2018). Isolates belonging to *Cadophora*, *Gibellulopsis*, *Hypoxylon*, and other common genera such as *Aspergillus* and *Alternaria* were obtained in a rather low abundance. This could be attributed to sampling size and location since sampling was centralized at a specific point on the shore of the Windebyer Noor. The isolates, which showed sequence similarities less than 97% to available GenBank entries (**Supplementary Table 1**), will be further investigated to ascertain their taxonomic novelty. In addition, some uncommon and understudied fungal groups were isolated, such as the representatives of the family Lindgomycetaceae and the genera *Plenodomus* and *Cosmospora*. Only a minute proportion of the microbiota of any given environment will grow under laboratory conditions (Sansonetti, 2018). Furthermore, even on the agar plates, faster-growing fungi may out-compete the slower growing fungi, which could escape detection. However, the present study reveals for the first time an insight into the cultivable fungal community of the Windebyer Noor, their chemical inventory, and potential to produce bioactive SMs when challenged with economically relevant plant pathogens.

The marine-adapted fungal isolates for co-cultivation experiments were selected based on the genetic difference since SM profiles are known to be species-specific and useful for fungal species recognition (Larsen et al., 2005; Frisvad et al., 2007, 2008). Some strains, however, could not be re-cultured or gave extremely low amounts of organic extracts. Hence, in total 21 out of the 30 selected genera were used for further research (**Table 2**). The genus *Penicillium* was excluded from this study as it is already a well-studied genus with many metabolites reported with anti-phytopathogenic activity (Li et al., 2014; Tang et al., 2015).

### Bioactivity of Nine Co-cultures

The bioactivity of the selected nine co-cultures and their respective mono-culture extracts was assessed against six phytopathogens. All phytopathogens, except for *E. amylovora*, were sensitive to at least one extract. The control and treatment of *E. amylovora* outbreaks remain difficult and no synthetic compound with systemic properties against this pathogen is currently available (Aćimović et al., 2015). Most of the co-culture extracts involving *M. oryzae* as the phytopathogenic challenger showed comparable IC_50_ values to the corresponding mono-cultures of the marine-adapted fungi (**Table 3**). Although co-cultivation did not increase the anti-phytopathogenic activity of the marine fungi, the overall chemical diversity was enhanced in all co-cultures as shown in their respective MNs (**Supplementary Figures 5–13**). Similar results were obtained in a previous co-cultivation study between *T. harzianum* and the phytopathogenic fungus *Pythium ultimum*, where the induction of anthraquinones in the co-culture did not translate into significant antifungal activity (Vinale et al., 2009). It is important to mention that we extracted and tested the whole co-cultures, and not the excised confrontation zones, to ensure enough extract for chemical and bioactivity profiling. The whole co-culture extracts contain hundreds of compounds, hence the concentrations of the specifically induced SMs may remain minor (Eyre et al., 2010) and their activity may escape detection.

Despite the lack of enhanced anti-phytopathogenic activity, we were able to detect several induced clusters with well-known antibiotic, anti-phytopathogenic, as well as cytotoxic activities, all relevant for growth inhibition of competing strains in a confined co-culture experiment. We also observed the overexpression of the compounds with relevant bioactivities or production of new members of clusters. The most striking example is represented by the exclusive production of the cephalosporins through challenging the marine fungus *Emericellopsis* sp. with the phytopathogenic bacterium *P. syringae*, further corroborating the successful stimulation of antibiotic biosynthesis by co-cultivation (Netzker et al., 2018). Antibiosis is an established interaction mechanism between microbes, including phytopathogens. Another example is azaphilone polyketides (mitorubrins), where a specific, putatively novel analog was detected only in the co-culture of *Hypoxylon* sp. and *M. oryzae* (**Figure 4A** and **Supplementary Figure 7**). Azaphilones exhibit antifungal activity against devastating phytopathogens, e.g., *B. cinerea*, *Rhizopus oryzae*, and *Phytophthora capsici* (Osmanova et al., 2010; Cao et al., 2015). Mitorubrin was a major compound in the mono-culture of *Hypoxylon* sp. whereas the co-cultivation led to the enhanced biosynthesis of its derivatives. This is especially true for mitorubrinic acid, for which we recorded a twofold increase in the co-culture. Mitorubrinol and mitorubrinic acid were suggested as virulence factors of *Penicillium marneffei* (Woo et al., 2012). Our data suggest that co-cultivation may have triggered the expression of genes in *Hypoxylon* sp. to enhance the production of virulence factors for competitive advantage.

Alteration of cultivation parameters such as media composition was employed to assess microbial chemical diversity. Two media (PDA and SA), particularly the PDA appeared to be the best medium for co-cultivation of both fungus/fungus and fungus/bacterium pairs. It was the medium selected for the growth of seven out of the nine most bioactive co-cultures (**Table 2**). PDA medium, rich in both polysaccharides (potato starch) and the monosaccharide (dextrose), was apparently favored by both bacteria and fungi. The next section “dereplication and annotation of molecular clusters,” includes more discussions on induced metabolites in co-cultures, including mycotoxins and their relevant bioactivities.

### Dereplication and Annotation of Molecular Clusters

Dereplication of complex natural extracts is a challenging task. Molecular weight and formula prediction with HRMS are often inconclusive for dereplication. The fragmentation pattern, however, represents a specific feature for a certain compound class. Structure and chemical properties of molecules dictate the observable fragmentation patterns in the gas phase during an MS/MS experiment. The fragmentation patterns, which are similar for related compounds, are therefore used as proxies for chemical relatedness. Molecular networks organize the MS/MS spectra of metabolites present in an extract according to their fragment similarities, which can be visualized by a suitable software, e.g., Cytoscape. MN uses the GNPS platform (Wang et al., 2016), which integrates a publicly available spectral library against which the experimental MS/MS spectra are compared. Herein we combined GNPS with an ISDB–UNPD (Allard et al., 2016) into the dereplication process, allowing annotation of the unknown derivatives of known metabolites and revealing putatively new molecular clusters. Manual dereplication considering peak intensities between culture types (mono- and co-cultures) from the raw spectral data were also made for quantitative comparisons.

More than 20% of the peak ions analyzed in this study were produced only in co-cultures (**Figure 3**). MN-aided dereplication (coupled to *in silico* MS/MS and manual dereplication) efforts allowed putative identification of 10% of all peak ions and 5% of the induced peak ions in co-cultures in contrast to the average annotation rate of about 1.8% in classical untargeted metabolomics approaches (Quinn et al., 2017). The remaining induced peak ions did not match to any known compound in multiple databases. The novelty of these yet unidentified peak ions needs to be proven after their purification and characterization. The global MN analysis has revealed that 41 compounds produced by the plant pathogens were absent in the co-culture extracts. The phytotoxins nortenuazonic acid and ergone produced by *M. oryzae* and *B. cinerea* respectively, represent such examples. *Vice versa*, 34 compounds produced solely by the marine-adapted fungi were absent in the co-culture extracts. The antifungal cyclocitrinol (Kozlovsky et al., 2000) and the polyketide 3,4-dihydro-mitorubrinol acetate were putatively annotated in the single culture extracts of *Acremonium* sp. and *Hypoxylon* sp., respectively, but not in their respective co-culture extracts. These examples indicate the presence of also negative effects between the phytopathogens and the target marine-adapted fungi, causing a silencing of biosynthetic pathways. Our results hence underline the complexity and dynamic nature of fungal metabolism and microbial interactions. The functional meaning and the grade of targeted silencing by specific co-cultivation partners still remains to be studied but may represent an important application angle for development of biocontrol agents.

The metabolites dereplicated from the mono- and co-cultures have various bioactivities reported in the literature. For example, the MN of *Hypoxylon* sp. and *M. oryzae* cultures revealed the presence of cytochalasins (**Supplementary Figure 7**). Cytochalasins are macrocyclic indole alkaloids mostly reported from ascomycetes and basidiomycetes with antimicrobial, cytotoxic, and phytotoxic activities (Buchanan et al., 1996; Chen et al., 2014; Yu et al., 2016). Cytochalasin J and seven other ions were exclusively induced in the co-culture. Cytochalasins have been suggested to provide a competitive advantage for the producing strain over other microorganisms with similar trophic preferences (Scherlach et al., 2010). A recent study showed that co-cultivation of *Aspergillus flavipes* with *Streptomyces* sp. from the same marine environment activated the production of six cytochalasin-type alkaloids by *A. flavipes* (Yu et al., 2016). Our results suggest that cytochalasins play similar roles in fungal crosstalk.

Further examples are the peptaibol antibiotic clusters (emerimicins and zervamicin) annotated in the MNs of *Emericellopsis* sp. and all its co-cultures (**Supplementary Figures 5**, **6**). Peptaibols belong to a class of α-aminoisobutyric acid containing peptides. In the MN, emerimicins and zervamicins formed two different clusters because of differences in their fragmentation patterns. Peptaibols are known for antibacterial and antifungal activities against phytopathogens (Ren et al., 2009). Argoudelis and Johnson (1974) reported the isolation and antimicrobial activity of emerimicins II, III, and IV from *Emericellopsis microspora*. These may be responsible for the distance inhibition against the phytopathogenic challengers observed on the agar plates (**Supplementary Figure 4**). The lack of an increase in bioactivity in this study may be related to their low concentration in the whole co-culture extract. Statistical analysis (**Figure 5B**) and the MN of *Emericellopsis* co-cultures revealed the induction of many unidentified ions, e.g., *m/z* [M+H]^+^ 1204.7736. The putative structure (peptide) proposed for this metabolite lacked a correctly identified C-terminal amino acid (**Figure 5C**). This could be worth pursuing since there is no record of the proposed amino acid sequence in literature, hence suggesting a new compound. The co-culture of *Emericellopsis* sp. with *P. syringae* induced five new clusters (>2 nodes), one of which was annotated as cephalosporins (**Supplementary Figure 5**). The other new clusters remain unknown.

Analysis of the interaction zone observed in the co-culture of *Cosmospora* sp. and *M. oryzae* revealed four significant peaks (numbered 1–4, **Figure 7**) highly induced in this area. The *t*_R_ of compounds 1 and 2 (approx. 6 min, total elution time is 12.5 min) indicate their medium polarity, whereas compounds 3 and 4 (*t*_R_ approx. 9 min) are more lipophilic. Dereplication efforts resulted in only a few compounds of plant origin, which do not fit to the spectral fragments obtained. These four compounds, three (1–3) of which are closely related, may represent new metabolites and our future efforts will include their isolation, characterization, and activity assessments. The MN of *Cosmospora* sp. co-cultured with *M. oryzae* and *P. syringae* identified ustilaginoidin type polyketides as the largest cluster (**Supplementary Figures 12**, **13**). Some putatively annotated members of this cluster in mono- and co-culture extracts include ustilaginoidins A, E, D, and V. Cephalochromin, a putatively annotated member of this cluster has previously been shown to exhibit strong activity against oomycetes and fungal phytopathogens (Kock et al., 2009).

*Alternaria* mycotoxins are categorized into three groups based on their structural classes (Amatulli et al., 2013), two of which, namely dibenzopyrone derivatives and tetramic acid derivatives, were annotated in the MN of *Alternaria* sp. and its co-culture with *P. syringae.* Several dibenzopyrone-type polyketides (alternariol, altenuene, and alternariol monomethyl ether) were detected in both mono- and co-culture of the marine-adapted *Alternaria* sp. The tetramic acid derivative tenuazonic acid was enhanced in the co-culture. These mycotoxins have been reported for their strong cytotoxicity (Qin et al., 2009) and could be responsible for the dark coloration of *P. syringae* after 2 weeks of co-cultivation with *Alternaria* sp. (**Figure 2D**).

Co-culture of *Acremonium* sp. with *P. syringae* promoted the induction of three new clusters (constituting more than 2 nodes) in the co-cultures (see the MN in **Supplementary Figure 10**). Majority of the pseudomolecular ions observed for the induced compounds were not annotated and can be putative new compounds. The major molecular cluster in the co-culture of *Acremonium* sp. with *P. syringae* was however annotated as ophiobolin type sesterterpenes with reported antimicrobial and phytotoxic activities (Sohsomboon et al., 2018; Zhu et al., 2018). The ecological role of ophiobolins still remains elusive. Our dereplication effort shows for the first time the presence of ophiobolins in a marine-adapted *Acremonium* sp. This species showed 96% ITS sequence similarity to its closest relative *Acremonium breve* (order Hypocreales) and warrants further studies.

The present co-cultivation study involved microbes obtained from completely different habitats. The marine-adapted fungi were obtained from an unstudied brackish environment and put into a competing environment with devastating phytopathogenic strains. By nature, phytopathogens are competitive microbes representing promising models in co-cultivation studies. They have been used as efficient challengers against plant endophytes (Combès et al., 2012; Tata et al., 2015), other pathogens (Müller et al., 2012; Weikl et al., 2016) and large variety of fungi from different environments (Serrano et al., 2017) to either enhance the production of constitutively present metabolites or trigger entirely new biosynthetic pathways to yield new and bioactive metabolites (Müller et al., 2012; Marmann et al., 2014). This study, however, represents the first co-cultivation attempt of marine-adapted fungi with phytopathogens for discovery of novel anti-phytopathogenic agents. Based on the results obtained in this study, the marine-adapted strains *Cosmospora* sp. and *Acremonium* sp. and their co-cultures with *M. oryzae* and *P. syringae*, respectively, are prioritized for large-scale fermentation followed by purification and structure elucidation studies to identify their bioactive constituents.

## Summary and Perspective

The unique environment, Windebyer Noor, selected for this study proved to be an excellent source of diverse fungi. Isolation efforts yielded a total number of 123 fungal isolates, resulting in potentially new isolates, which will be further analyzed to establish their taxonomic identity. Co-cultivation of marine-adapted fungi with phytopathogenic challengers was a successful means for increasing the chemical space of the marine-adapted fungi; however, it did not cause an exponential increase of bioactivity in whole co-culture extracts. Future studies will focus on assessing the bioactivity of the confrontation zone extracts. The combination of the MN, ISDB, and manual dereplication efforts significantly facilitated metabolome analyses and dereplication. The MN did not only reveal the structural relationships between the SMs but also enabled rapid annotation of related compounds in a cluster and revealed potentially new derivatives in annotated chemical families. Moreover, several clusters contained ions that could not be annotated suggesting that they may also be putative novel compounds.

This study confirms that phytopathogens are useful partners for co-cultivation studies and highlights the complex chemical interactions between phytopathogens with marine-adapted fungi. Our research has also revealed the potential of marine-adapted fungi as a source of biocontrol agents in agriculture. The combination of chemical and bioactivity profiling assisted in the prioritization of two co-culture pairs for large-scale cultivation and down-stream purification studies.

## Data Availability

The datasets generated/analyzed for this study are included in the manuscript and the **Supplementary Files**.

## Author Contributions

EO-D, AL, and DT designed the experiments. EO-D performed all experiments. EO-D, DP, and MB analyzed the data. EO-D, DP, MB, AL, and DT wrote the manuscript.

## Conflict of Interest Statement

The authors declare that the research was conducted in the absence of any commercial or financial relationships that could be construed as a potential conflict of interest.

## Footnotes

1 http://www.umweltdaten.landsh.de/db/dbnuis?thema=see&seenummer=0447

2 https://blast.ncbi.nlm.nih.gov/Blast.cgi?PROGRAM=blastn&PAGE_TYPE=BlastSearch&LINK_LOC=blasthome

3 https://gnps.ucsd.edu

4 http://www.cytoscape.org/
