# Supplementary material for: Molecular Networking-Based Metabolome and Bioactivity Analyses of Marine-Adapted Fungi Co-cultivated With Phytopathogens
Source: Front Microbiol. 2018 Sep 6;9:2072. doi: 10.3389/fmicb.2018.02072 (PMC6135897; doi:10.3389/fmicb.2018.02072)
Supplement: Supplementary file 1 [file Data_Sheet_1.pdf]

Supplementary Material

Molecular Networking-Based Metabolome and Bioactivity Analyses of Marine-Adapted Fungi Co-Cultivated with Phytopathogens

Ernest Oppong-Danquah, Delphine Parrot, Martina Blümel, Antje Labes and Deniz Tasdemir*

*** Correspondence:** Deniz Tasdemir: dtasdemir@geomar.de

Supplementary Data

List of Supplementary Figures

Supplementary Figure 1. Map showing the sampling location (circled red) in the Windebyer Noor close to the town of Eckernförde in the German state of Schleswig-Holstein. Retrieved from https://www.openstreetmap.org/#map=12/54.4689/9.7806

Supplementary Figure 2. Pie charts showing the diversity and distribution of the 123 marine-adapted fungal isolates obtained from the Windebyer Noor. **(A)** Diversity of the fungal isolates using GPY medium, **(B)** diversity of the fungal isolates using HS medium, **(C)** diversity of the fungal isolates using PCA medium and, **(D)** diversity of the fungal isolates obtained using WSP medium, **(E)** diversity of isolates at the order level, **(F)** diversity of the isolates at genus level, **(G)** distribution of the isolates by isolation source.

Supplementary Figure 3. Workflow for the co-cultivation study based on the 123 fungal isolates. 3 isolates (*Acremonium* sp., *Microdochium* sp. and *Cladosporium* sp.) were selected for initial optimization of co-cultivation parameters. The optimized conditions were applied to a selection of 21 genetically diverse strains, which were subsequently co-cultured with 4 phytopathogens in 2 media (SA and PDA) yielding 168 co-cultures and compared to the respective monocultures of the marine-adapted strains and the phytopathogens. 9 interesting co-cultures from 5 marine-adapted fungi were selected for in-depth metabolomics analyses including the molecular networking (MN) based dereplication approach and IC_{50} determination.

Supplementary Figure 4. Pictures acquired after 21 days of cultivation of all 5 selected marine-adapted fungal isolates, phytopathogens and the co-cultures in different media. **(A)** 5 marine-adapted fungal isolates, **(B)** 3 phytopathogens used as challengers in the 9 co-cultures, **(C)** 9 co-cultures.

Supplementary Figure 5. Annotated molecular network of extracts of *Emericellopsis* sp. (red), *P. syringae* (green) mono-cultures and their co-culture (blue) in SA medium.

Supplementary Figure 6. Annotated molecular network of extracts of *Emericellopsis* sp. (red), *M. oryzae* (green) mono-cultures and their co-culture (blue) in SA medium.

Supplementary Figure 7. Annotated molecular network of extracts of *Hypoxylon* sp. (red), *M. oryzae* (green) mono-cultures and their co-culture (blue) in PDA medium.

Supplementary Figure 8. Annotated molecular network of extracts of *Alternaria* sp. (red), *P. syringae* (green) mono-cultures and their co-culture (blue) in PDA medium.

Supplementary Figure 9. Annotated molecular network of extracts of *Acremonium* sp. (red), *B. cinerea* (green) mono-cultures and their co-culture (blue) in PDA medium.

Supplementary Figure 10. Annotated molecular network of extracts of *Acremonium* sp. (red), *P. syringae* (green) mono-cultures and their co-culture (blue) in PDA medium.

Supplementary Figure 11. Annotated molecular network of extracts of *Acremonium* sp. (red), *M. oryzae* (green) mono-culture and their co-culture (blue) in PDA medium.

Supplementary Figure 12. Annotated molecular network of extracts of *Cosmospora* sp. (red), *P. syringae* (green) mono-cultures and their co-culture (blue) in PDA medium.

Supplementary Figure 13. Annotated molecular network of extracts of *Cosmospora* sp. (red), *M. oryzae* (green) mono-cultures and their co-culture (blue) in PDA medium.

Supplementary Figure 14. Base peak chromatograms of the whole agar extract of *Cosmospora* sp. mono-culture **(A)**, *M. oryzae* mono-culture **(C)** and their co-culture in PDA medium **(B)** showing putatively annotated peak ions. Chromatogram of blank PDA medium is also displayed (bottom). Numbers correspond to metabolites listed in dereplication table (**Supplementary Table 2**). Identical peak ions identified in both marine-adapted fungal isolate and co-culture were annotated in chromatogram of marine-derived fungus only.

Supplementary Figure 15. Base peak chromatograms of whole agar extract of *Cosmospora* sp. mono-culture **(A)**, *P. syringae* mono-culture **(C)** and their co-culture in PDA medium **(B)** showing putatively identified peak ions. Chromatogram of blank PDA medium is also displayed **(D)**. Number corresponds to metabolites listed in dereplication table (**Supplementary Table 2**). Identical peak ions identified in both marine-derived fungal isolate and co-culture were annotated in chromatogram of marine-adapted fungus only.

Supplementary Figure 16. Base peak chromatograms of whole agar extract of *Emericellopsis* sp. mono-culture **(A)**, *P. syringae* mono-cultures **(C)** and their co-culture in SA medium **(B)** showing putatively identified peak ions. Chromatogram of blank SA medium is also displayed **(D)**. Numbers correspond to metabolites listed in dereplication table (**Supplementary Table 2**). Identical peak ions identified in both marine-adapted fungal isolate and co-culture were annotated in chromatogram of marine-adapted fungus only. Peak ions induced in co-culture are additionally annotated in co-culture chromatogram.

Supplementary Figure 17. Base peak chromatograms of whole agar extracts of *Emericellopsis* sp. mono-culture **(A)**, *M. oryzae* mono-culture **(C)** and their co-culture in SA medium **(B)** showing putatively identified peak ions. Chromatogram of blank SA medium is also displayed **(D)**. Numbers correspond to metabolites listed in dereplication table (**Supplementary Table 2**). Identical peak ions identified in both marine-adapted fungal isolate and co-culture were annotated in chromatogram of

marine-adapted fungus only. Peak ions induced in co-culture are additionally annotated in co-culture chromatogram.

Supplementary Figure 18. Base peak chromatograms of whole agar extracts of *Hypoxylon* sp. mono-culture **(A)**, *M. oryzae* mono-culture **(C)** and their co-culture in PDA medium **(B)** showing putatively identified peak ions. Chromatogram of blank PDA medium is also displayed **(D)**. Numbers correspond to metabolites listed in dereplication table (**Supplementary Table 2**). Identical peak ions identified in both marine-adapted fungal isolate and co-culture were annotated in chromatogram of marine-adapted fungus only. Peak ions induced in co-culture are additionally annotated in co-culture chromatogram.

Supplementary Figure 19. Base peak chromatograms of whole agar extracts of *Alternaria* sp. mono-culture **(A)**, *P. syringae* mono-culture **(C)** and their co-culture in PDA medium **(B)** showing putatively identified peak ions. Chromatogram of blank PDA medium is also displayed **(D)**. Numbers correspond to metabolites listed in dereplication table (**Supplementary Table 2**). Identical peak ions identified in both marine-adapted fungal isolate and co-culture were annotated in chromatogram of marine-adapted fungus only.

Supplementary Figure 20. Base peak chromatograms of whole agar extracts of *Acremonium* sp. mono-culture **(A)**, *P. syringae* mono-culture **(C)** and their co-culture in PDA medium **(B)** showing putatively identified peak ions. Chromatogram of blank PDA medium is also displayed **(D)**. Number corresponds to metabolites listed in dereplication table (**Supplementary Table 2**). Identical peak ions identified in both marine-adapted fungal isolate and co-culture were annotated in chromatogram of marine-adapted fungus only. Peak ions induced in co-culture are additionally annotated in co-culture chromatogram.

Supplementary Figure 21. Base peak chromatograms of whole agar extracts of *Acremonium* sp. mono-culture **(A)**, *M. oryzae* mono-culture **(C)** and their co-culture in PDA medium **(B)** showing putatively identified peak ions. Chromatogram of blank PDA medium is also displayed **(D)**. Number corresponds to metabolites listed in dereplication table (**Supplementary Table 2**). Identical peak ions identified in both marine-adapted fungal isolate and co-culture were annotated in chromatogram of marine-adapted fungus only. Peak ions induced in co-culture are additionally annotated in co-culture chromatogram.

Supplementary Figure 22. Base peak chromatograms of whole agar extracts of *Acremonium* sp. mono-culture **(A)**, *B. cinerea* mono-culture **(C)** and their co-culture in PDA medium **(B)** showing putatively identified peak ions. Chromatogram of blank PDA medium is also displayed **(D)**. Numbers correspond to metabolites listed in dereplication table (**Supplementary Table 2**). Identical peak ions identified in both marine-adapted fungal isolate and co-culture were annotated in chromatogram of marine-adapted fungus only. Peak ions induced in co-culture are additionally annotated in co-culture chromatogram.

Supplementary Figure 23. Annotated MS/MS spectrum of emerimicin IV (*m/z* $[M+H]^+$ 1573.8994 based on MS/MS spectrum acquired in positive mode from 50 to 1600 Da). Each fragment is equivalent to an amino acid. Phe - phenylalanine, Aib - alpha-aminoisobutyric acid, Val - valine, Gly - glycine, Leu - leucine, Hyp - hydroxyproline, Gln - glutamine, Ala - alanine, Pheol - phenylalaninol

Supplementary Figure 24. Annotated MS/MS spectrum of a putatively new peptide of the Emerimicin family (*m/z* $[M+H]^+$ 1204.7736 based on MS/MS spectrum acquired in positive mode from 50 to 1600 Da). XX- unknown amino acid with *m/z* $[M+H]^+$ 110.0354 corresponding to a molecular formula of $C_4H_4N_3O$, AHV- 3-amino-2-hydroxyvaline, Aib- alpha- aminoisobutyric acid, Val- valine, Leu- leucine, Gly- glycine, Ala- alanine, AMO2- 2 amino- N,4-dimethyl-8-oxodecanoic acid

List of Supplementary Tables

Supplementary Table 1. Identification of 123 strains isolated from Windebyer Noor (Nov, 2015) according to sequence comparison with the NCBI nucleotide database using BLASTn. The 3 first BLAST hits incl. respective accession numbers are given. WO: twigs, F: Foam, SC: driftwood scrapings, L: leaves, WA: water, SE: sediment, SD: seeds. Media: GPY: Glucose Peptone Yeast, PCA: Potato Carrot agar, WSP: modified Wickerham medium and HS: Hastings medium.

Supplementary Table 2. Putatively identified compounds by dereplication of extracts using GNPS combined with an *in silico* MS/MS database (ISDB-UNPD) and manual dereplication based on DNP, REAXYS, MarinLit and SciFinder databases. Annotation considered the *m/z* $[M+H]^+$, biological source, retention time, predicted molecular formula and fragmentation pattern. Culture type; N: metabolite derived from a mono-culture, C: metabolite derived from co-culture.

1.1 Supplementary Figures

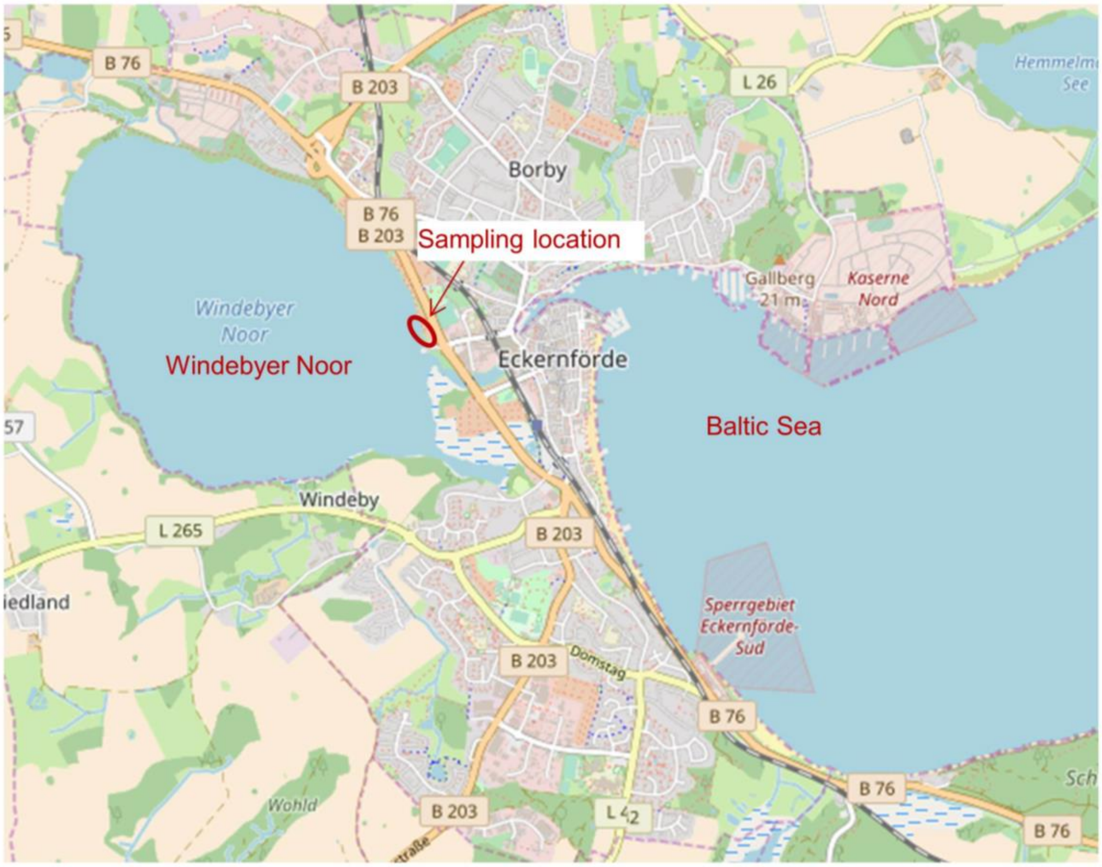

Supplementary Figure 1. Map showing the sampling location (circled red) in the Windebyer Noor close to the town of Eckernförde in the German state of Schleswig-Holstein. Retrieved from https://www.openstreetmap.org/#map=12/54.4689/9.7806

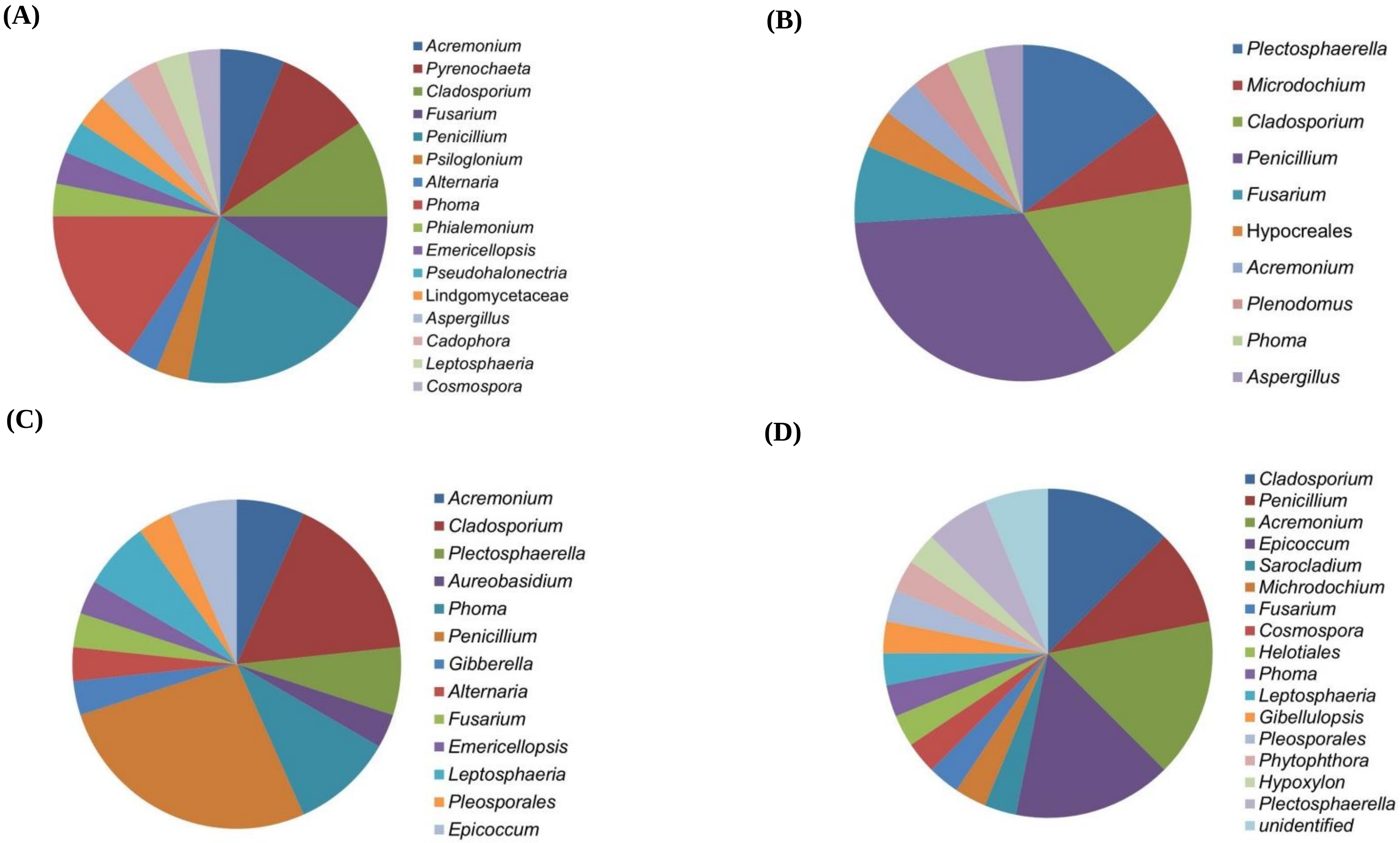
(A)
Acremonium
Pyrenochaeta
Cladosporium
Fusarium
Penicillium
Psiloglonium
Alternaria
Phoma
Phialemonium
Emericellopsis
Pseudohalonectria
Lindgomycetaceae
Aspergillus
Cadophora
Leptosphaeria
Cosmospora
(B)
Plectosphaerella
Microdochium
Cladosporium
Penicillium
Fusarium
Hypocreales
Acremonium
Plenodomus
Phoma
Aspergillus
(C)
Acremonium
Cladosporium
Plectosphaerella
Aureobasidium
Phoma
Penicillium
Gibberella
Alternaria
Fusarium
Emericellopsis
Leptosphaeria
Pleosporales
Epicoccum
(D)
Cladosporium
Penicillium
Acremonium
Epicoccum
Sarocladium
Michrodochium
Fusarium
Cosmospora
Helotiales
Phoma
Leptosphaeria
Gibellulopsis
Pleosporales
Phytophthora
Hypoxylon
Plectosphaerella
unidentified

(E)

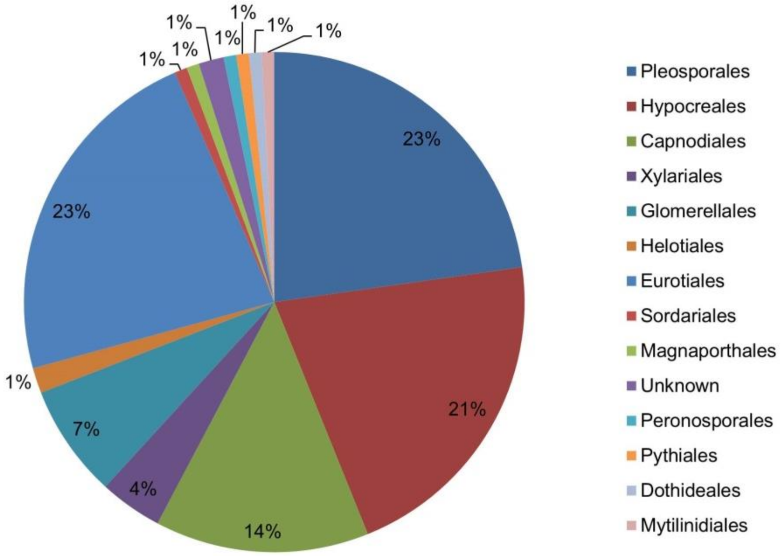

(F)

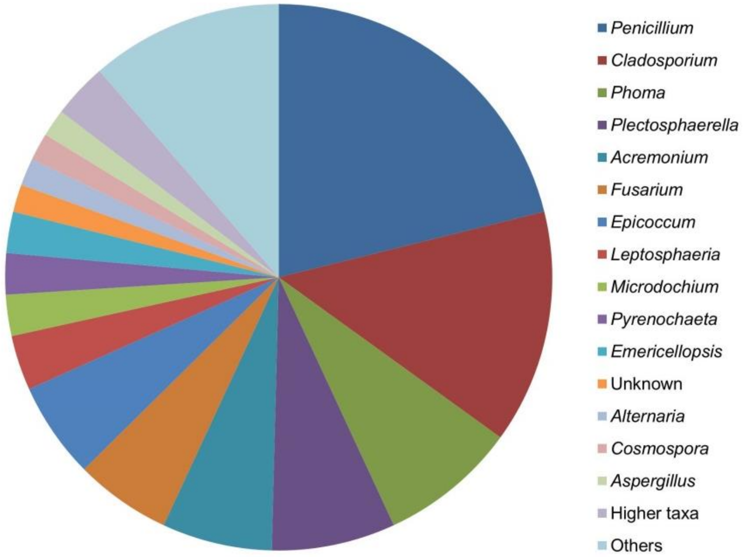

(G)

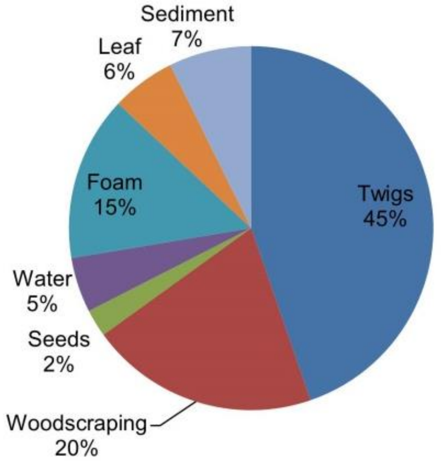

Supplementary Figure 2. Pie charts showing the diversity and distribution of the 123 marine-adapted fungal isolates obtained from the Windebyer Noor. **(A)** Diversity of the fungal isolates using GPY medium, **(B)** diversity of the fungal isolates using HS medium, **(C)** diversity of the fungal isolates using PCA medium and, **(D)** diversity of the fungal isolates obtained using WSP medium, **(E)** diversity of isolates at the order level, **(F)** diversity of the isolates at genus level, **(G)** distribution of the isolates by isolation source.

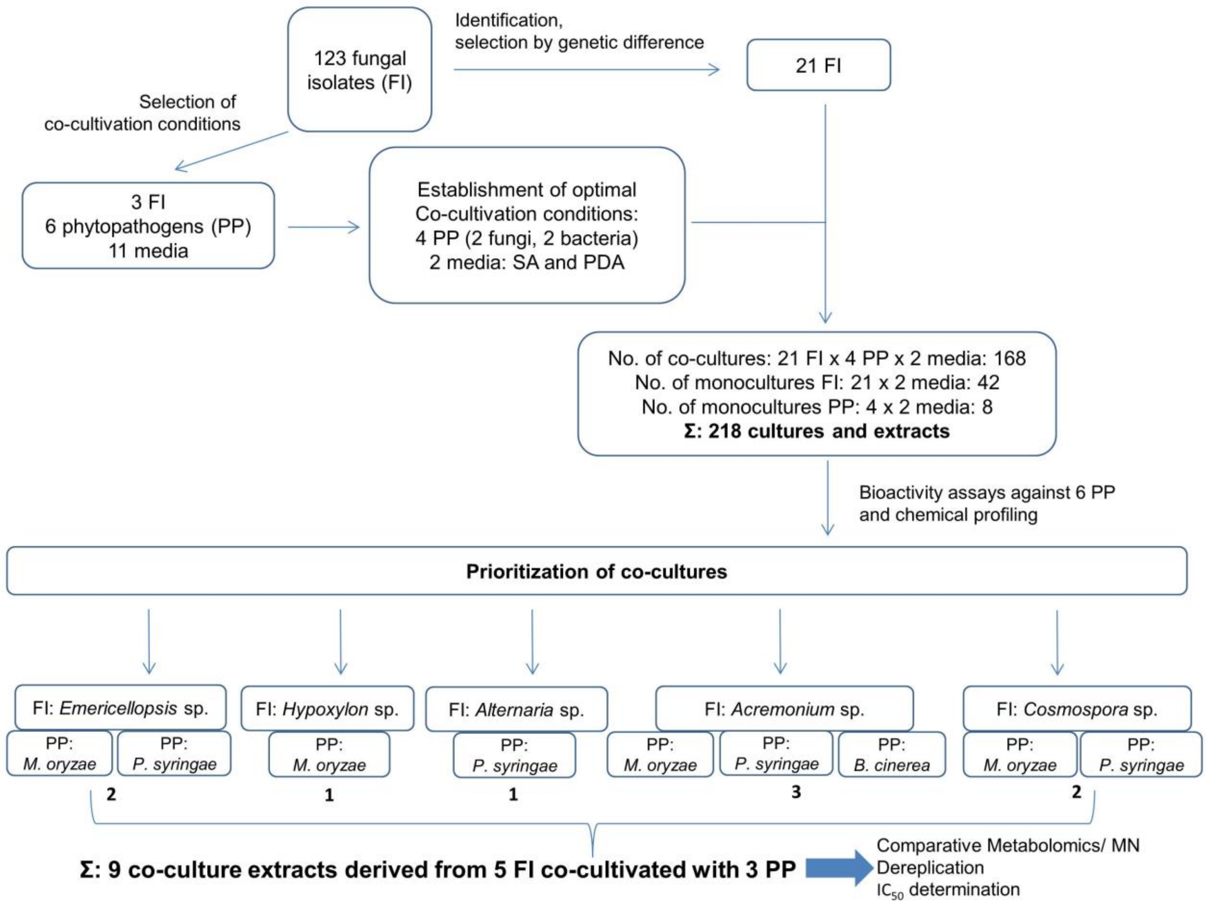

Supplementary Figure 3. Workflow for the co-cultivation study based on the 123 fungal isolates. 3 isolates (*Acremonium* sp., *Microdochium* sp. and *Cladosporium* sp.) were selected for initial optimization of co-cultivation parameters. The optimized conditions were applied to a selection of 21 genetically diverse strains, which were subsequently co-cultured with 4 phytopathogens in 2 media (SA and PDA) yielding 168 co-cultures and compared to the respective monocultures of the marine-adapted strains and the phytopathogens. 9 interesting co-cultures from 5 marine-adapted fungi were selected for in-depth metabolomics analyses including the molecular networking (MN) based dereplication approach and IC_{50} determination.

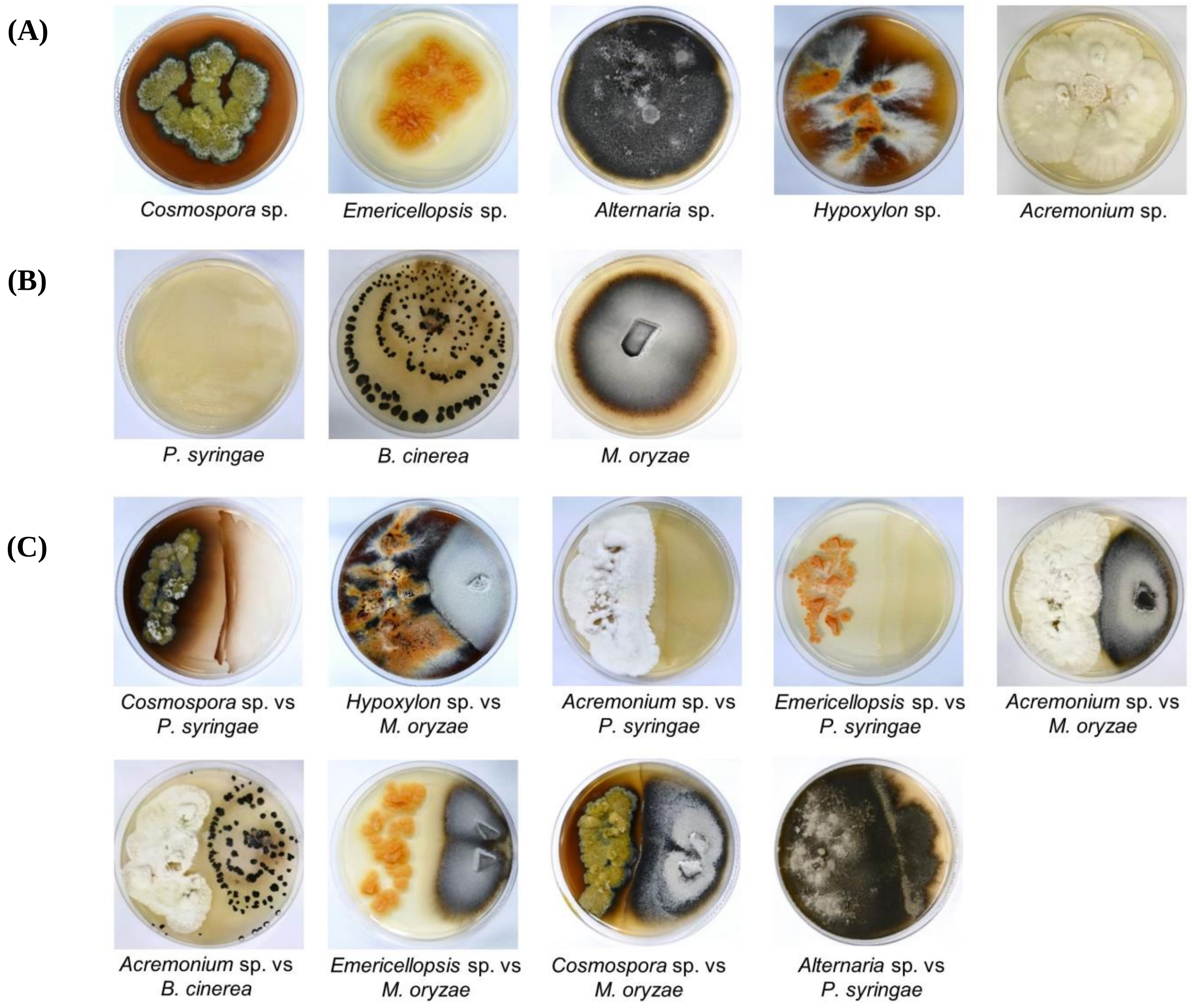

Supplementary Figure 4. Pictures acquired after 21 days of cultivation of all 5 selected marine-adapted fungal isolates, phytopathogens and the co-cultures in different media. **(A)** 5 marine-adapted fungal isolates, **(B)** 3 phytopathogens used as challengers in the 9 co-cultures, **(C)** 9 co-cultures.

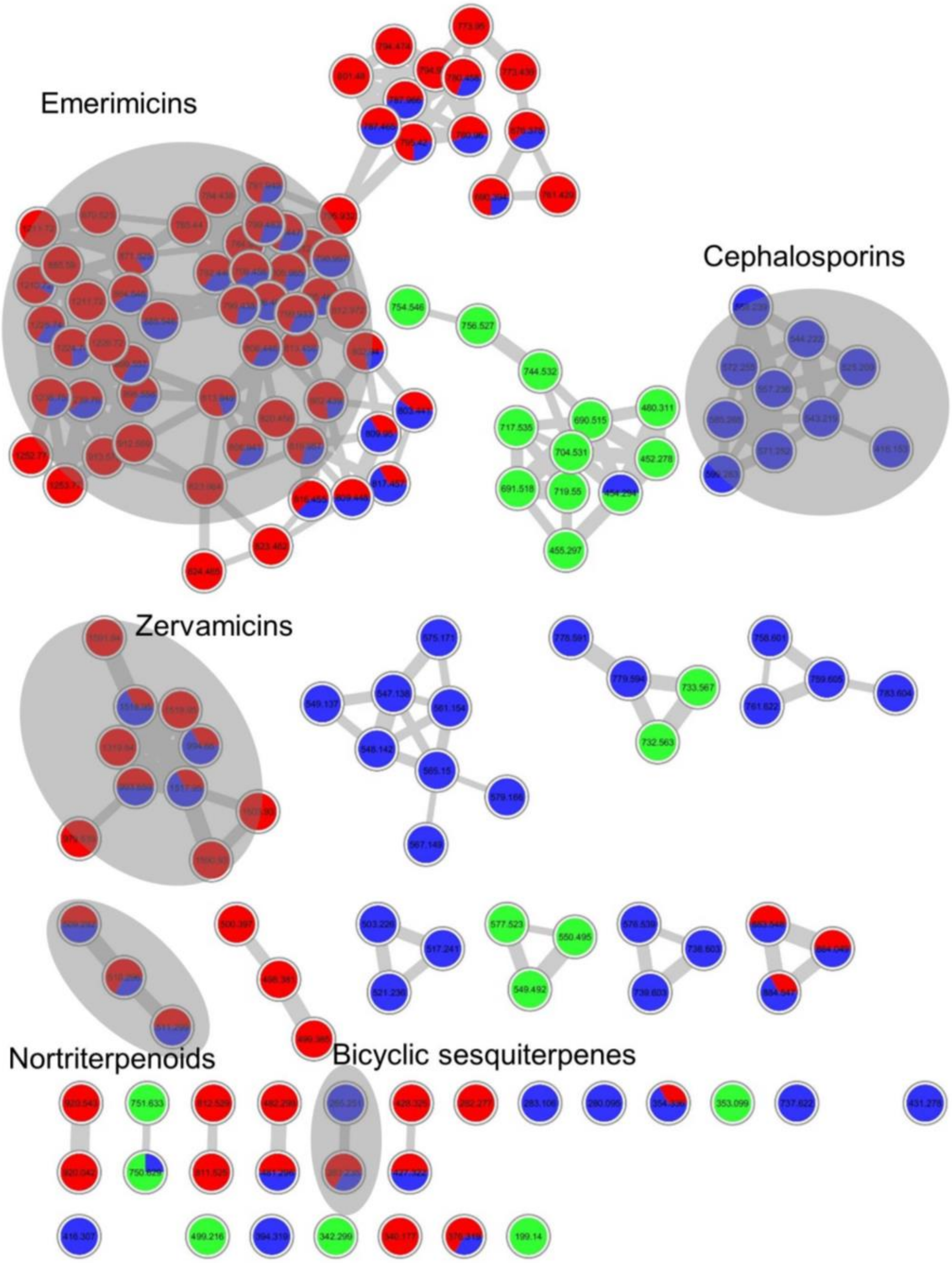

Supplementary Figure 5. Annotated molecular network of extracts of *Emericellopsis* sp. (red), *P. syringae* (green) mono-cultures and their co-culture (blue) in SA medium.

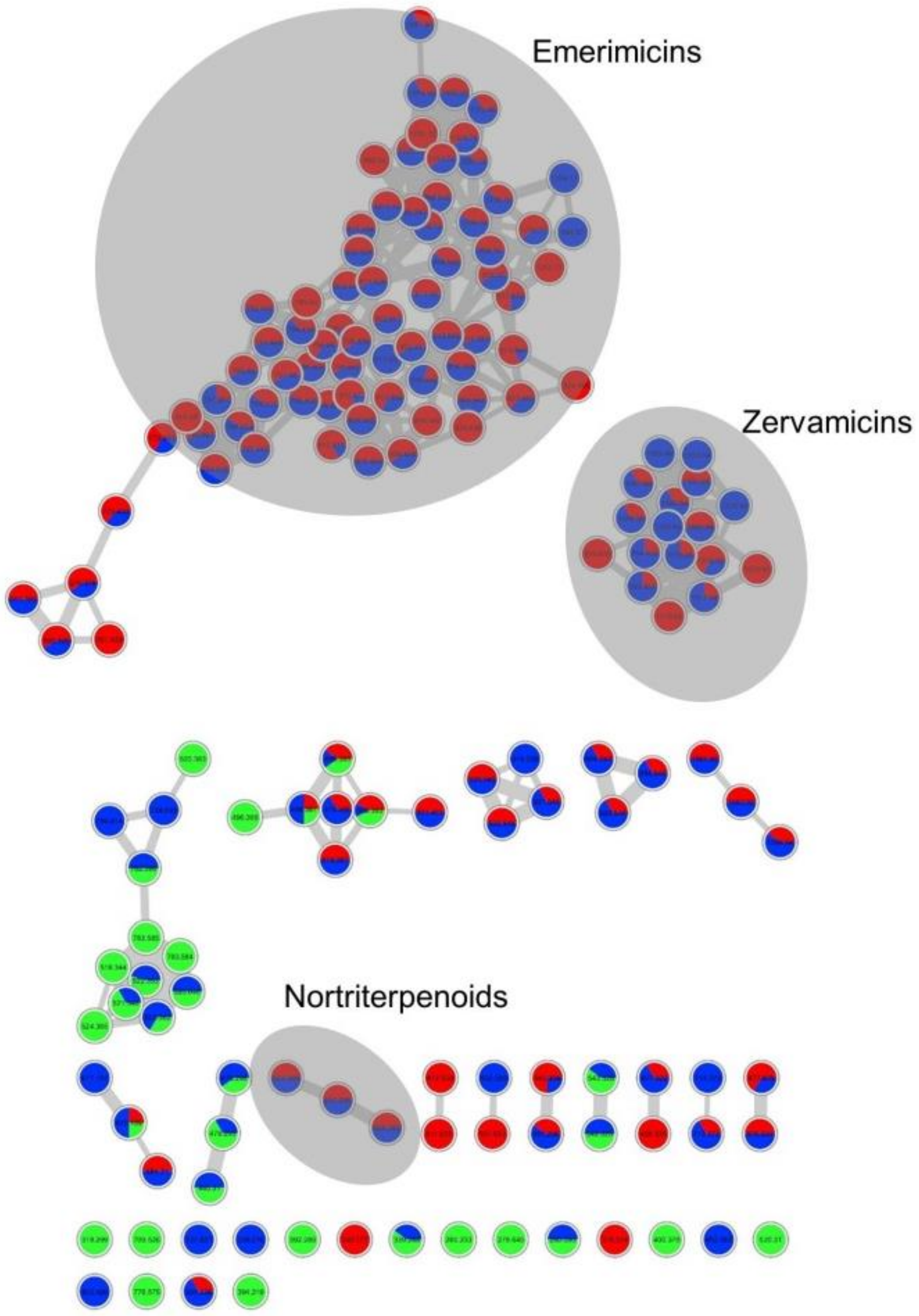

Supplementary Figure 6. Annotated molecular network of extracts of *Emericellopsis* sp. (red), *M. oryzae* (green) mono-cultures and their co-culture (blue) in SA medium.

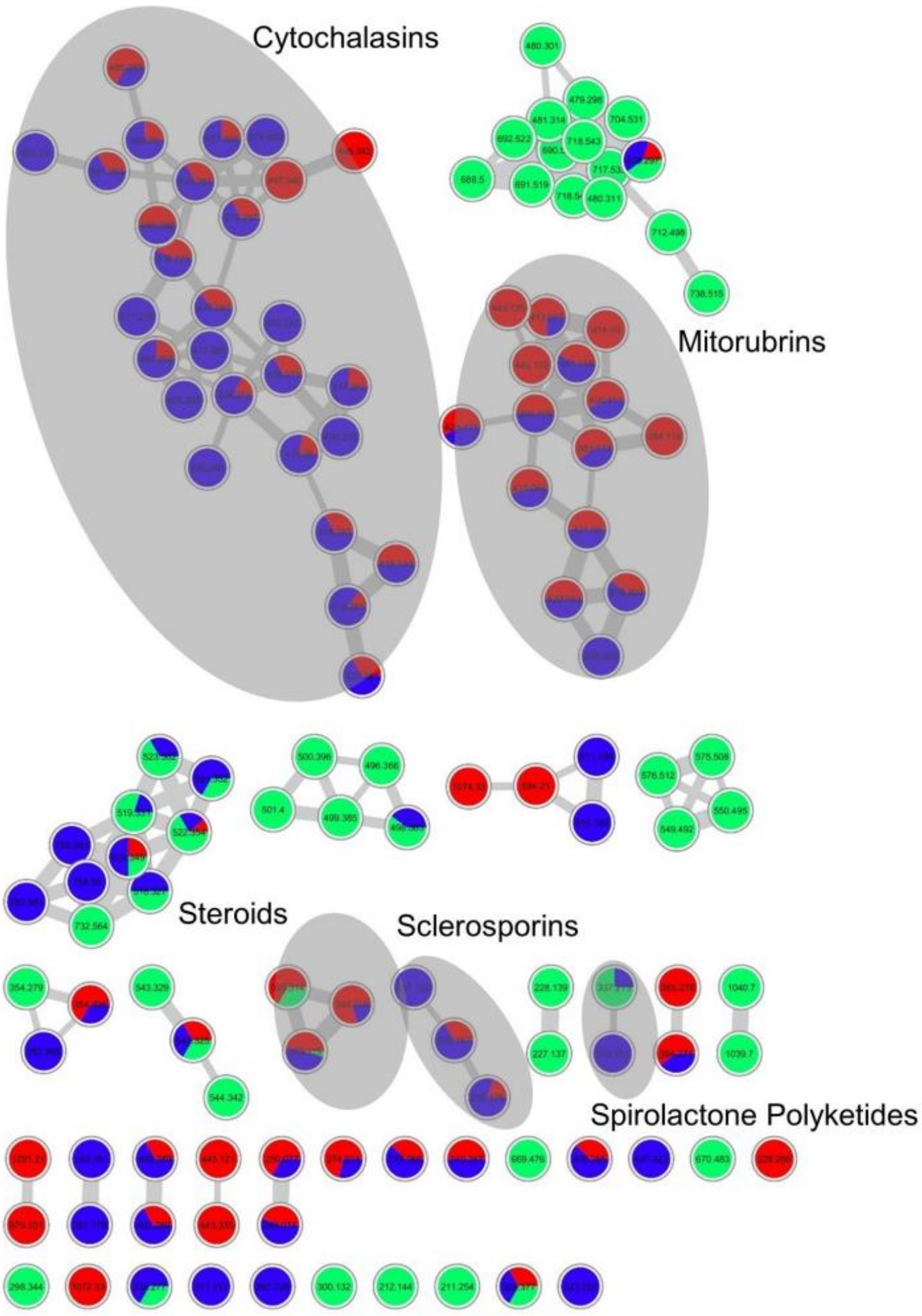

Supplementary Figure 7. Annotated molecular network of extracts of *Hypoxylon* sp. (red), *M. oryzae* (green) mono-cultures and their co-culture (blue) in PDA medium.

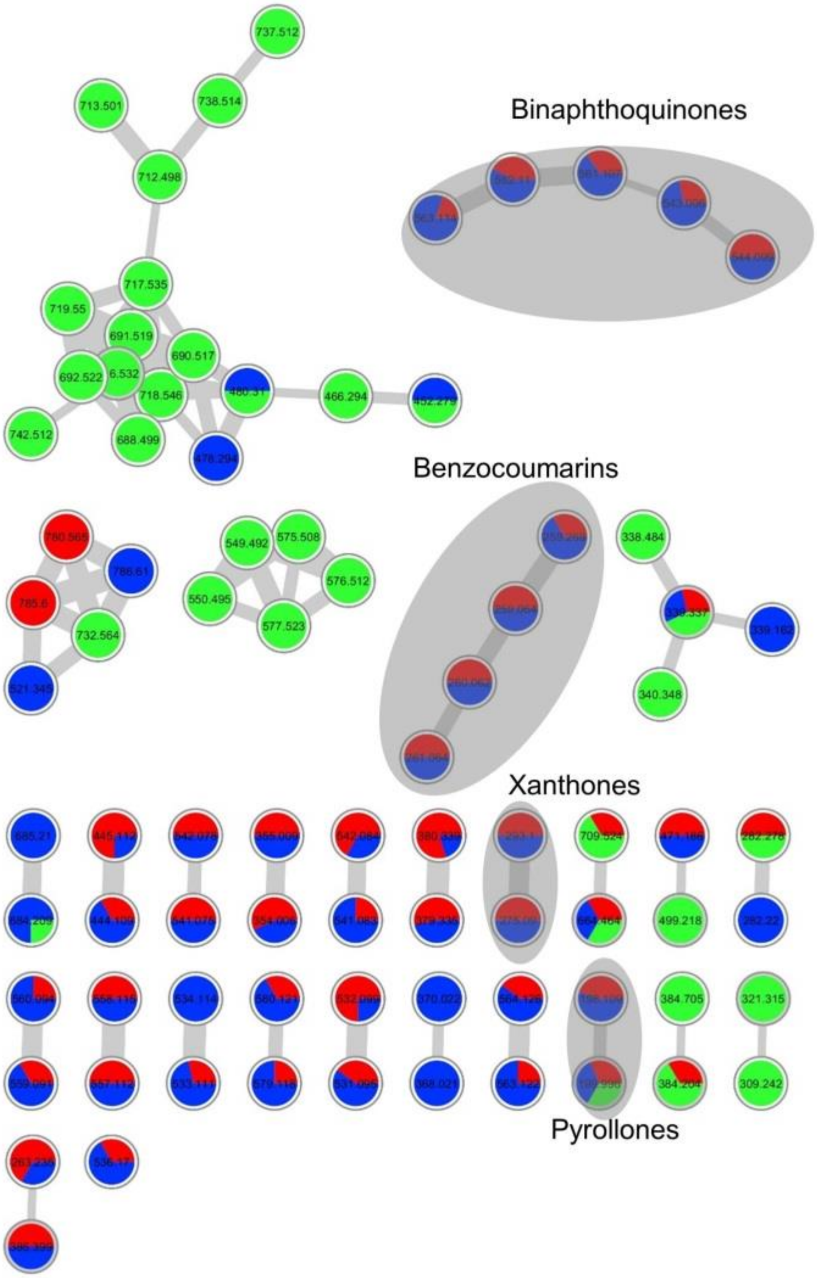

Supplementary Figure 8. Annotated molecular network of extracts of *Alternaria* sp. (red), *P. syringae* (green) mono-cultures and their co-culture (blue) in PDA medium.

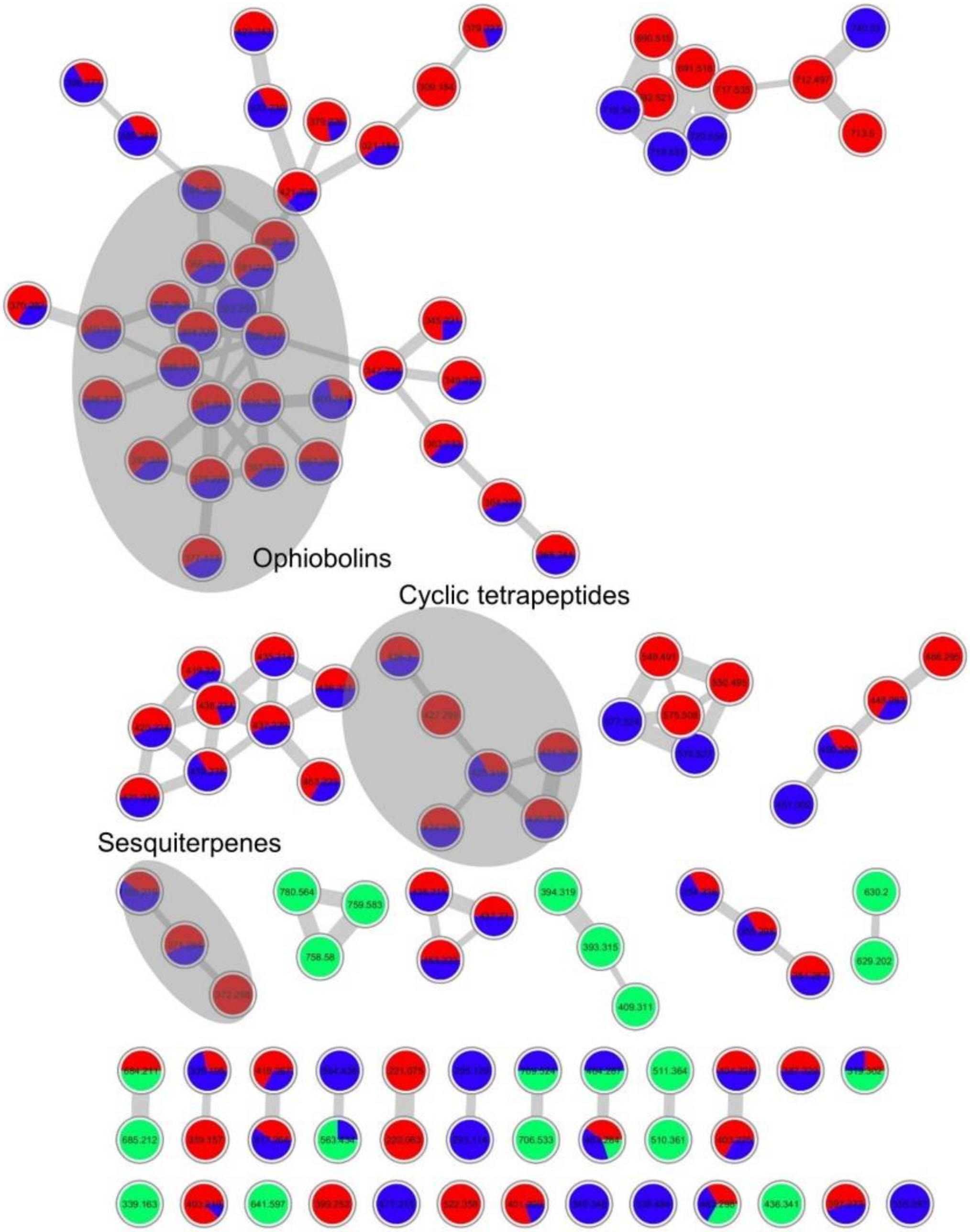

Supplementary Figure 9. Annotated molecular network of extracts of *Acremonium* sp. (red), *B. cinerea* (green) mono-cultures and their co-culture (blue) in PDA medium.

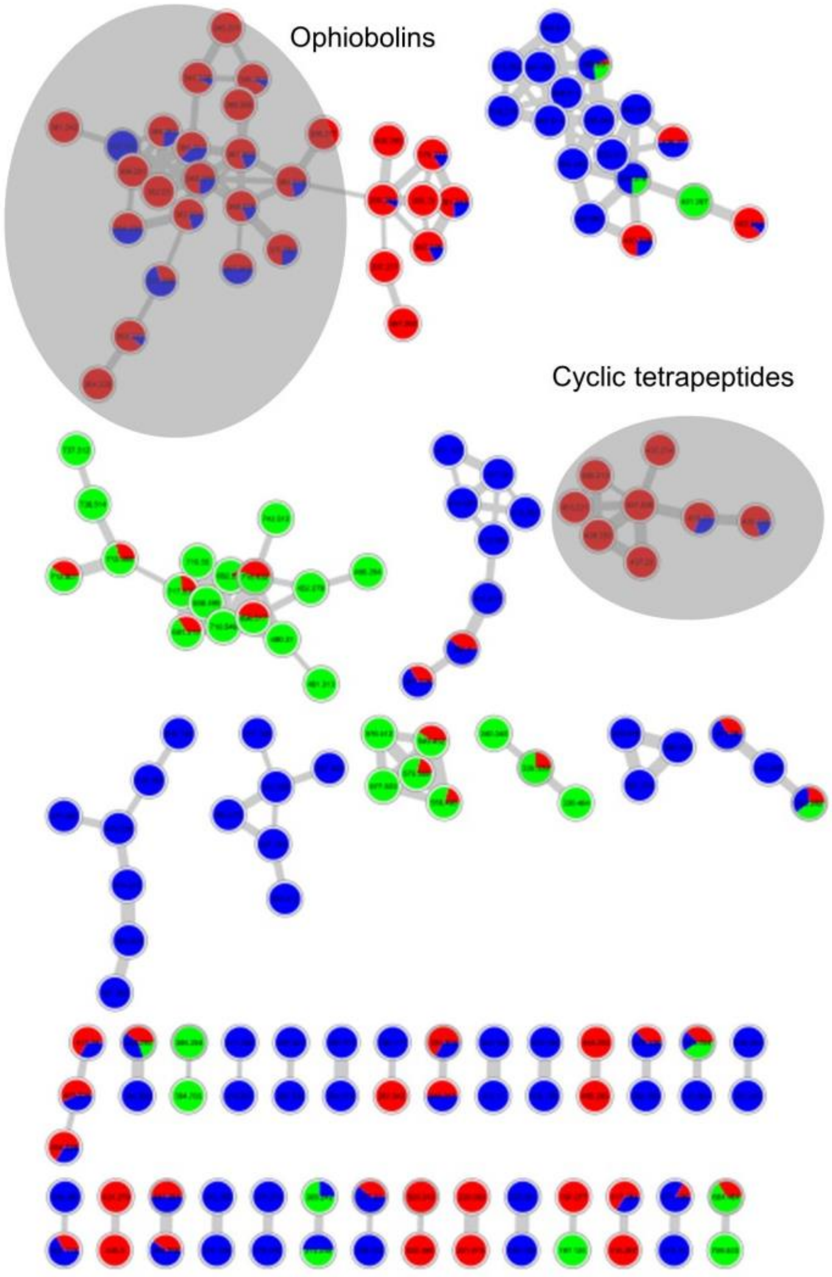

Supplementary Figure 10. Annotated molecular network of extracts of *Acremonium* sp. (red), *P. syringae* (green) mono-cultures and their co-culture (blue) in PDA medium.

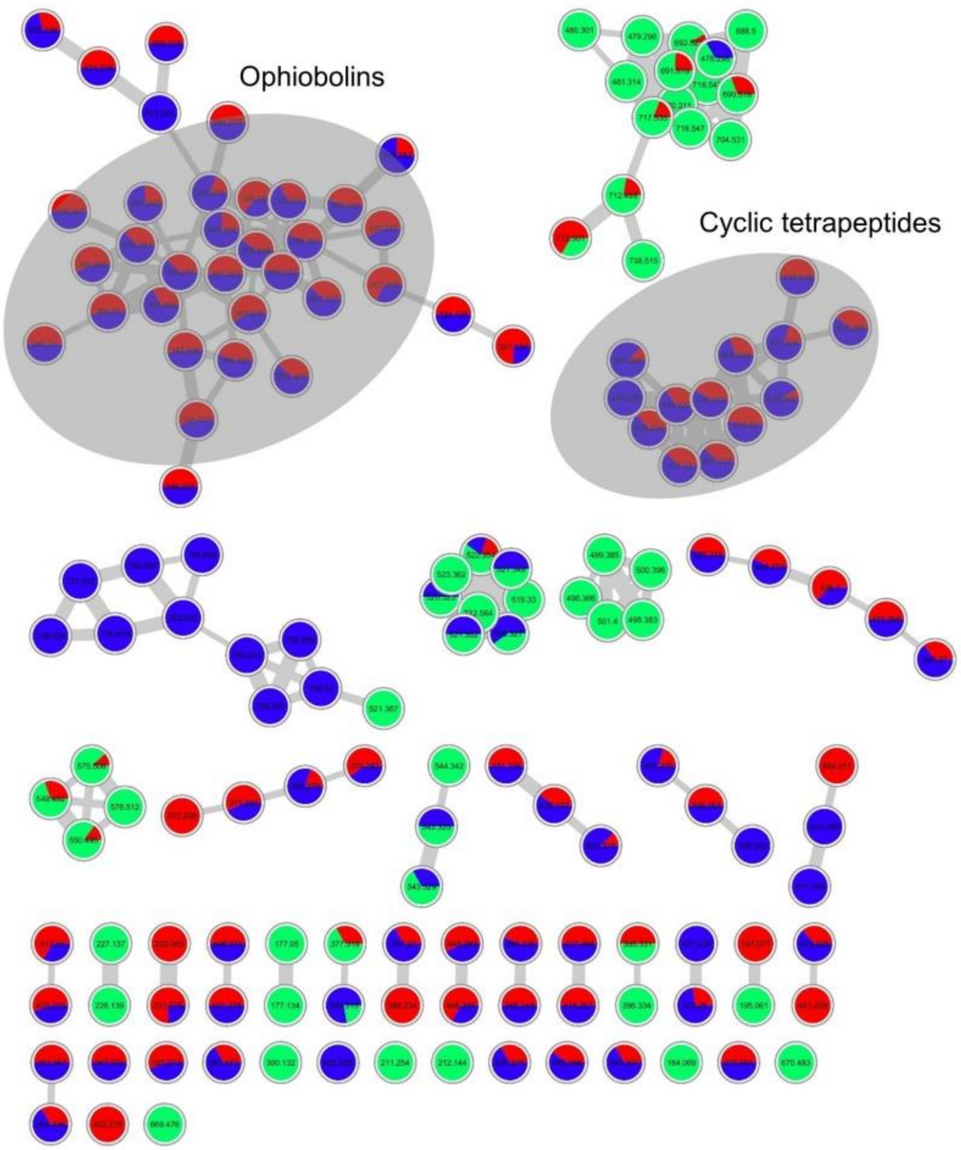

Supplementary Figure 11. Annotated molecular network of extracts of *Acremonium* sp. (red), *M. oryzae* (green) mono-culture and their co-culture (blue) in PDA medium.

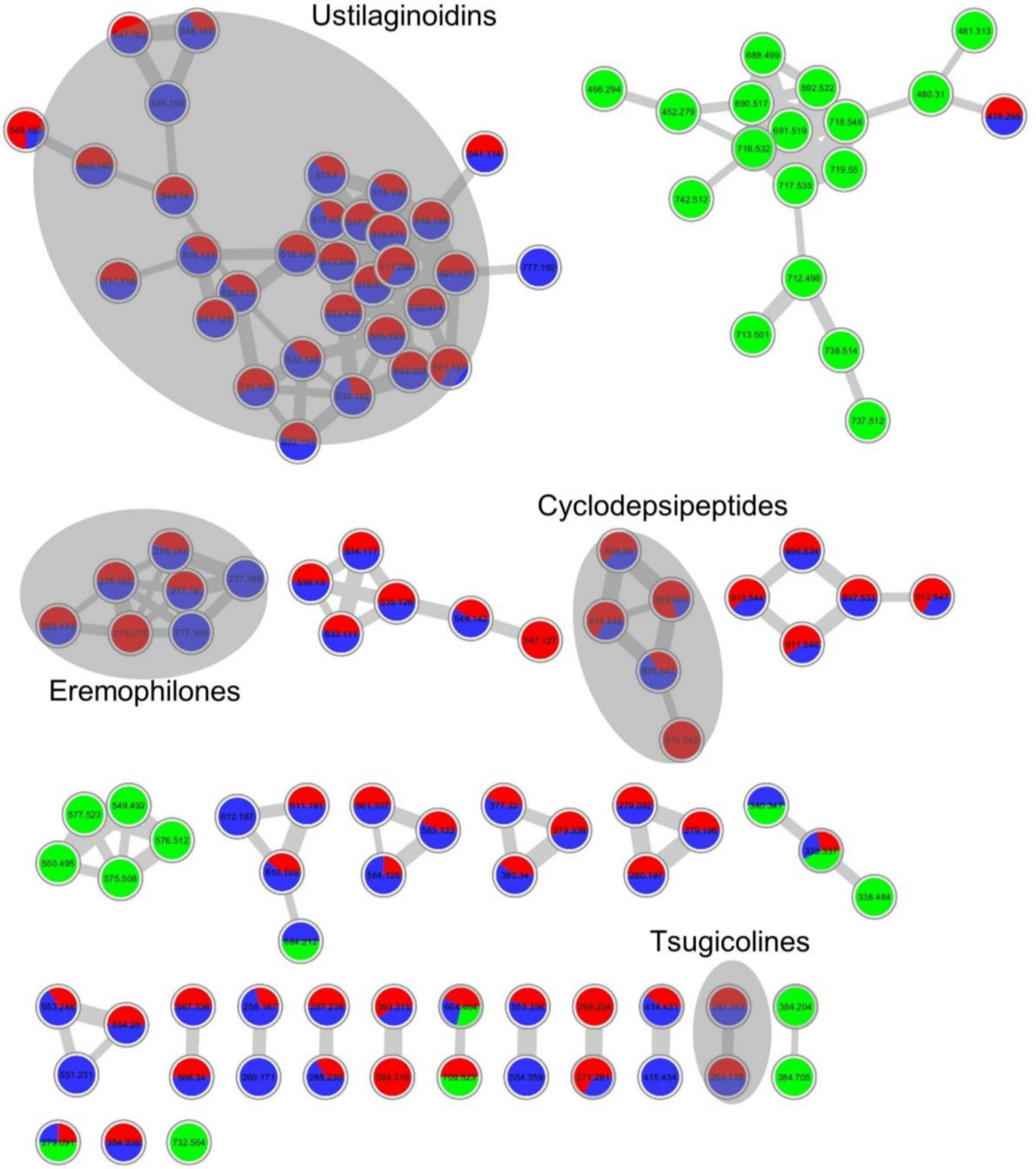

Supplementary Figure 12. Annotated molecular network of extracts of *Cosmospora* sp. (red), *P. syringae* (green) mono-cultures and their co-culture (blue) in PDA medium.

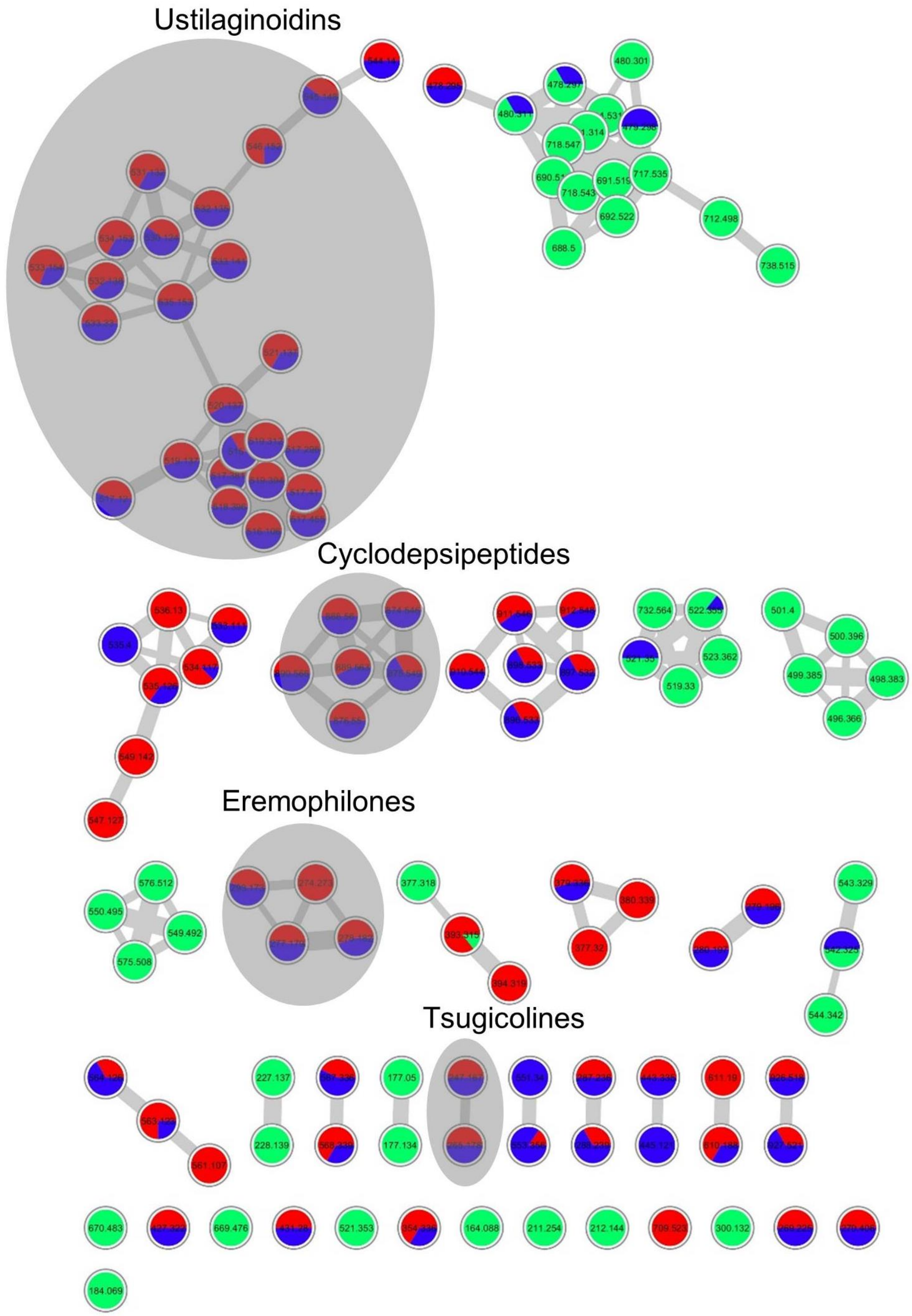

Supplementary Figure 13. Annotated molecular network of extracts of *Cosmospora* sp. (red), *M. oryzae* (green) mono-cultures and their co-culture (blue) in PDA medium.

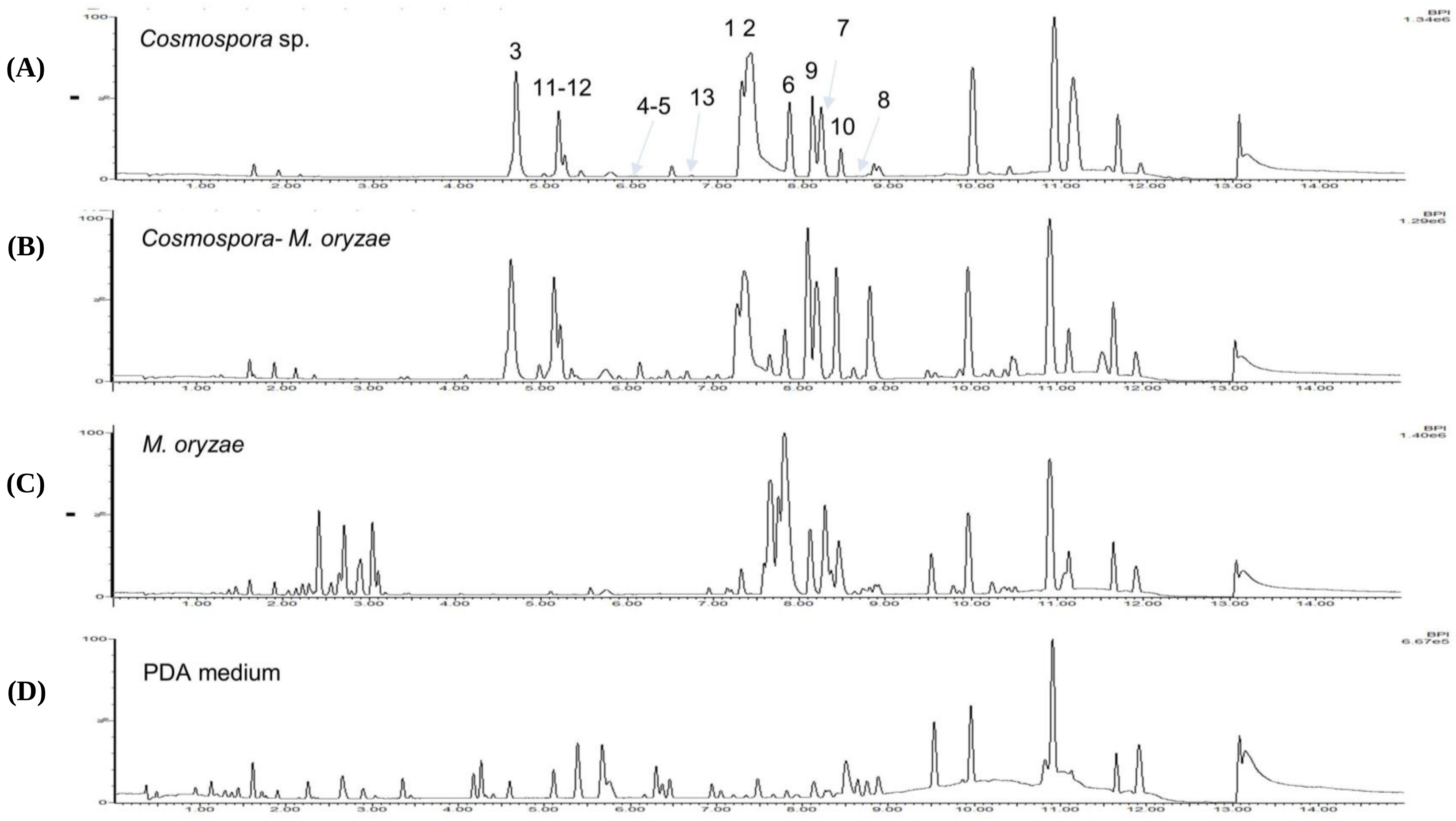

Supplementary Figure 14. Base peak chromatograms of the whole agar extract of *Cosmospora* sp. mono-culture **(A)**, *M. oryzae* mono-culture **(C),** and their co-culture in PDA medium **(B)** showing putatively annotated peak ions. Chromatogram of blank PDA medium is also displayed (**D**). Numbers correspond to metabolites listed in dereplication table (**Supplementary Table 2**). Identical peak ions identified in both marine-adapted fungal isolate and co-culture were annotated in chromatogram of marine-derived fungus only.

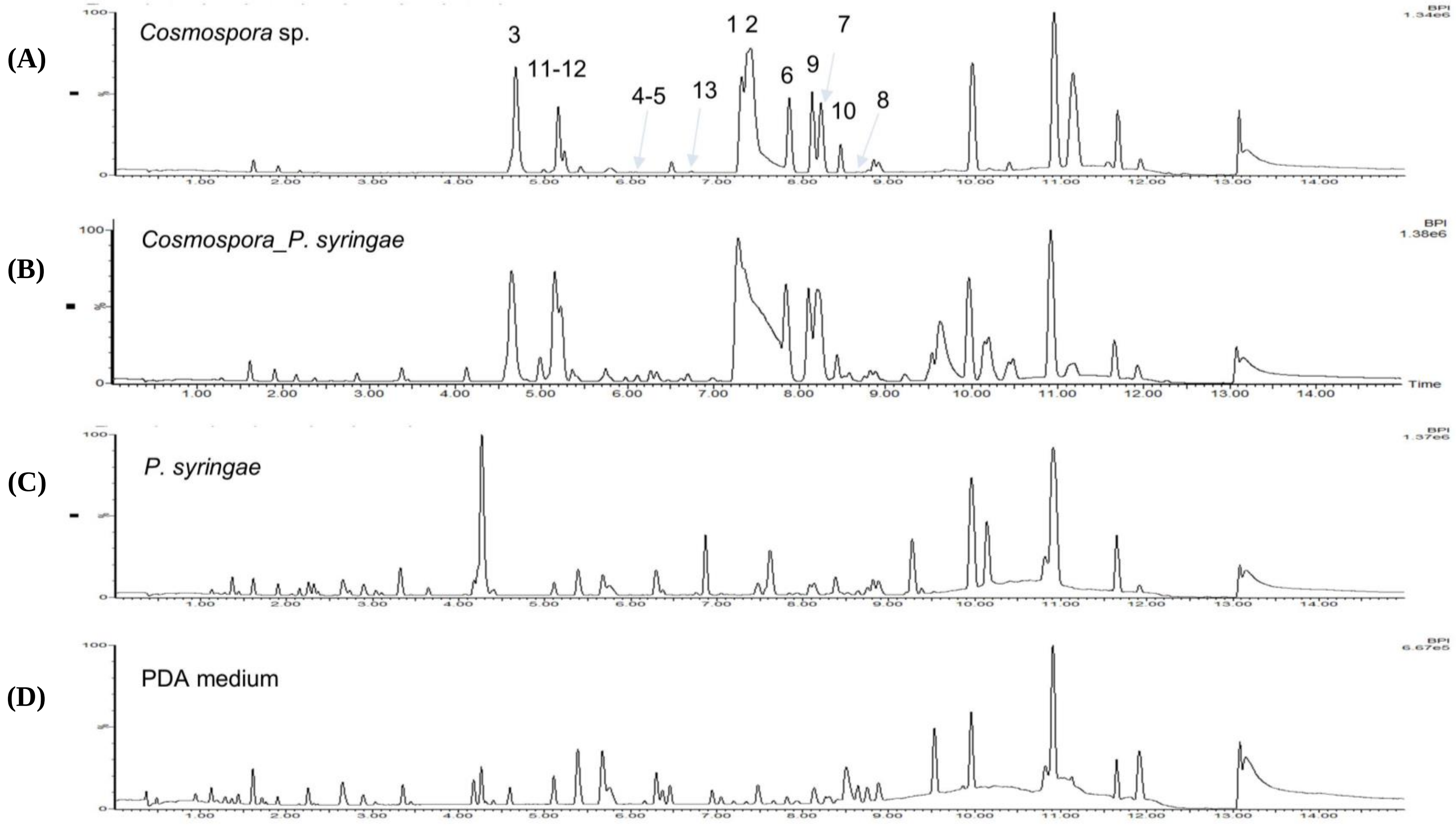

Supplementary Figure 15. Base peak chromatograms of whole agar extract of *Cosmospora* sp. mono-culture **(A)**, *P. syringae* mono-culture **(C),** and their co-culture in PDA medium **(B)** showing putatively identified peak ions. Chromatogram of blank PDA medium is also displayed **(D)**. Numbers correspond to metabolites listed in dereplication table (**Supplementary Table 2**). Identical peak ions identified in both marine-derived fungal isolate and co-culture were annotated in chromatogram of marine-adapted fungus only.

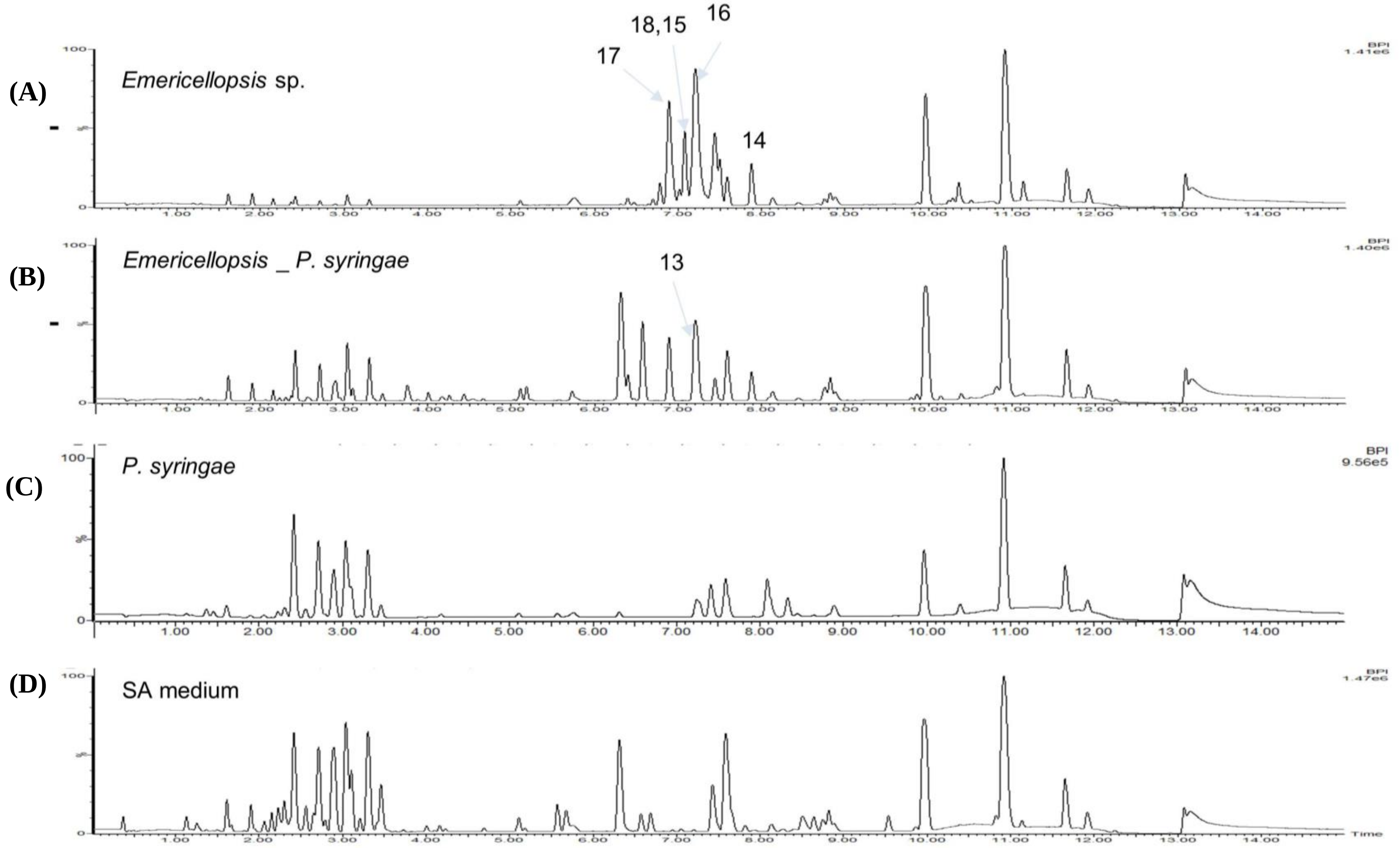

Supplementary Figure 16. Base peak chromatograms of whole agar extract of *Emericellopsis* sp. mono-culture **(A)**, *P. syringae* mono-cultures **(C),** and their co-culture in SA medium **(B)** showing putatively identified peak ions. Chromatogram of blank SA medium is also displayed **(D)**. Numbers correspond to metabolites listed in dereplication table (**Supplementary Table 2**). Identical peak ions identified in both marine-adapted fungal isolate and co-culture were annotated in chromatogram of marine-adapted fungus only. Peak ions induced in co-culture are additionally annotated in co-culture chromatogram.

(A)

(B)

(C)

(D)

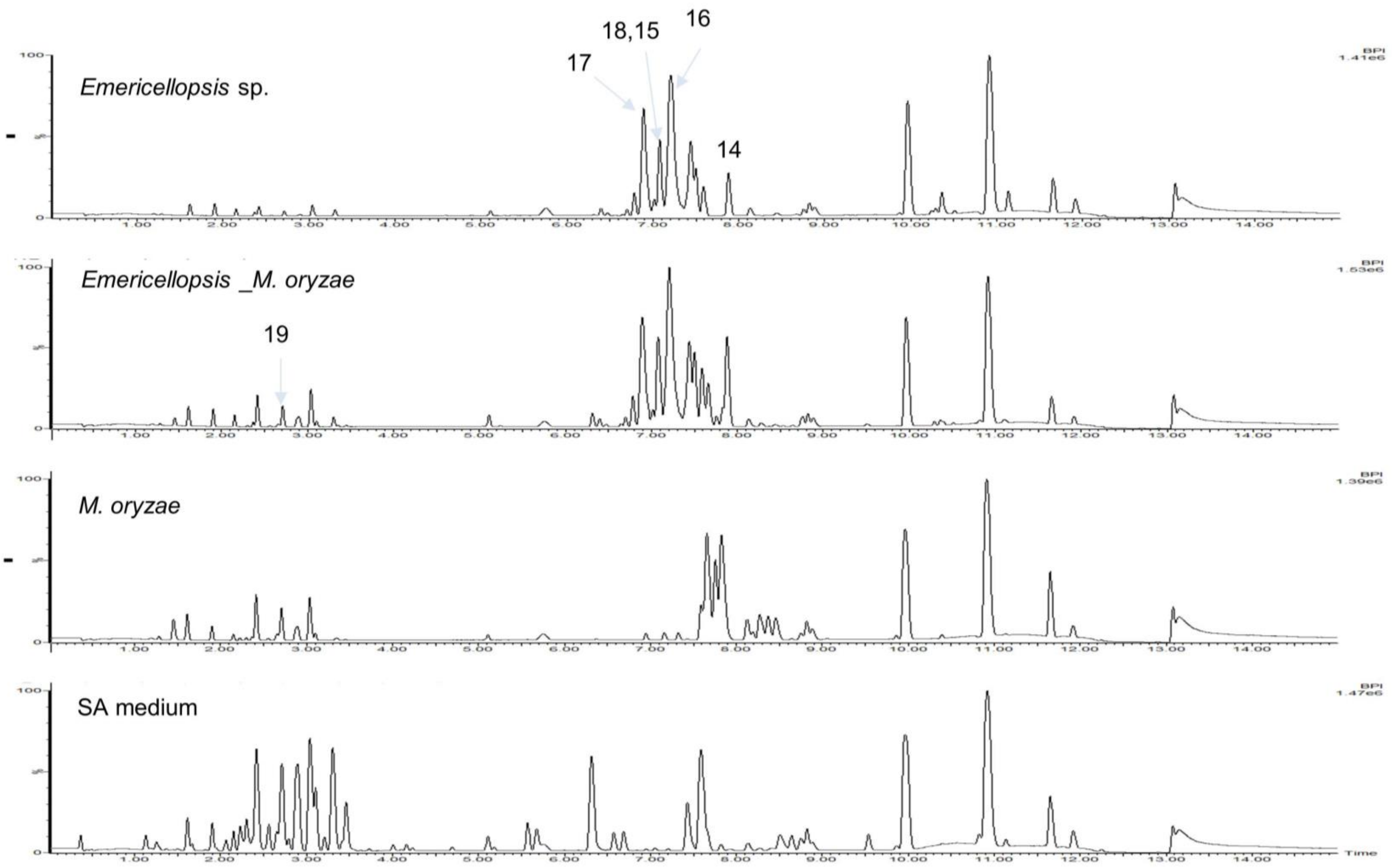

Supplementary Figure 17. Base peak chromatograms of whole agar extracts of *Emericellopsis* sp. mono-culture **(A)**, *M. oryzae* mono-culture **(C),** and their co-culture in SA medium **(B)** showing putatively identified peak ions. Chromatogram of blank SA medium is also displayed **(D)**. Numbers correspond to metabolites listed in dereplication table (**Supplementary Table 2**). Identical peak ions identified in both marine-adapted fungal isolate and co-culture were annotated in chromatogram of marine-adapted fungus only. Peak ions induced in co-culture are additionally annotated in co-culture chromatogram.

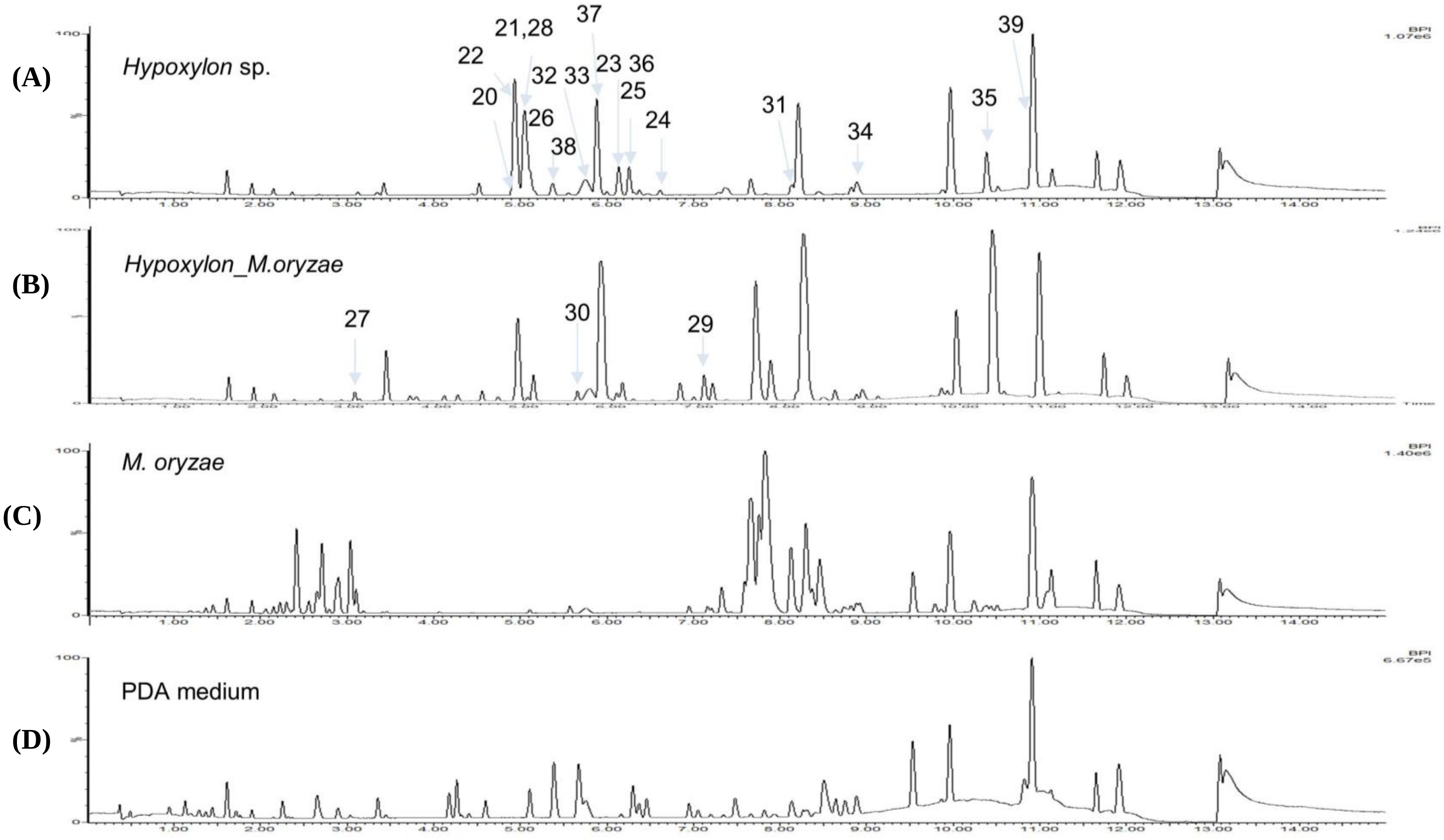

Supplementary Figure 18. Base peak chromatograms of whole agar extracts of *Hypoxylon* sp. mono-culture **(A)**, *M. oryzae* mono-culture **(C)** and their co-culture in PDA medium **(B)** showing putatively identified peak ions. Chromatogram of blank PDA medium is also displayed **(D)**. Numbers correspond to metabolites listed in dereplication table (**Supplementary Table 2**). Identical peak ions identified in both marine-adapted fungal isolate and co-culture were annotated in chromatogram of marine-adapted fungus only. Peak ions induced in co-culture are additionally annotated in co-culture chromatogram.

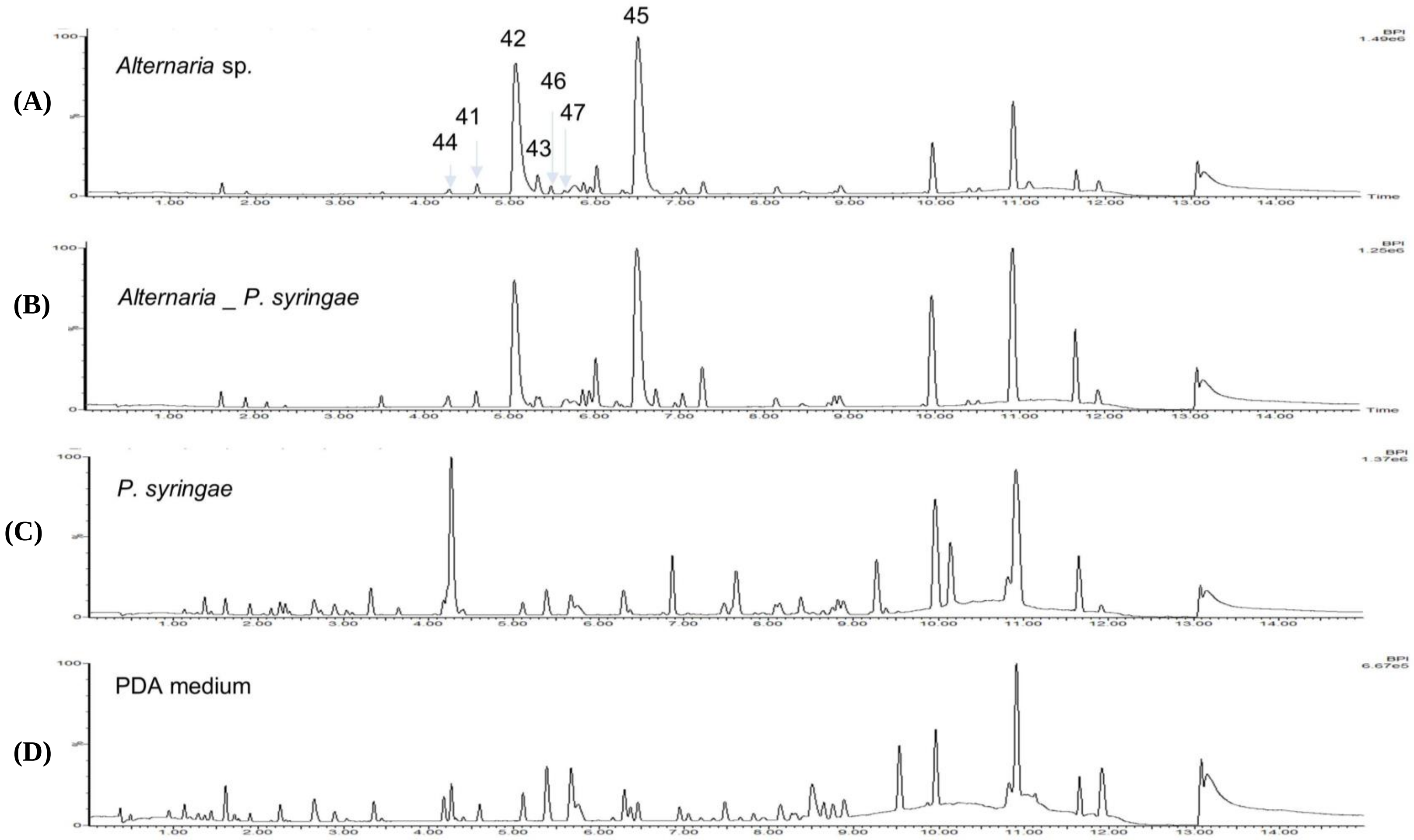

Supplementary Figure 19. Base peak chromatograms of whole agar extracts of *Alternaria* sp. mono-culture **(A)**, *P. syringae* mono-culture **(C),** and their co-culture in PDA medium **(B)** showing putatively identified peak ions. Chromatogram of blank PDA medium is also displayed **(D)**. Numbers correspond to metabolites listed in dereplication table (**Supplementary Table 2**). Identical peak ions identified in both marine-adapted fungal isolate and co-culture were annotated in chromatogram of marine-adapted fungus only.

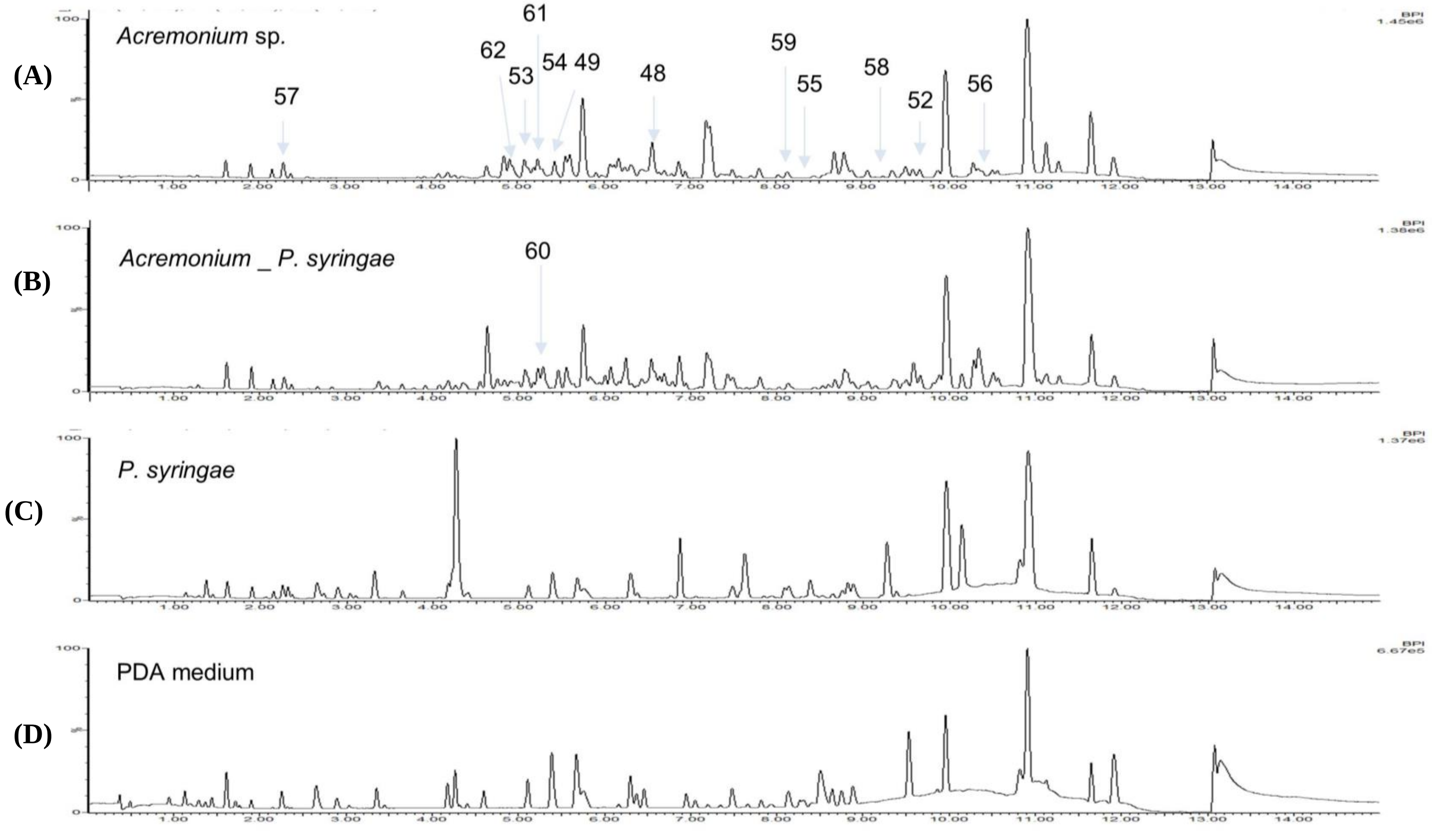

Supplementary Figure 20. Base peak chromatograms of whole agar extracts of *Acremonium* sp. mono-culture **(A)**, *P. syringae* mono-culture **(C)** and their co-culture in PDA medium **(B)** showing putatively identified peak ions. Chromatogram of blank PDA medium is also displayed **(D)**. Numbers correspond to metabolites listed in dereplication table (**Supplementary Table 2**). Identical peak ions identified in both marine-adapted fungal isolate and co-culture were annotated in chromatogram of marine-adapted fungus only. Peak ions induced in co-culture are additionally annotated in co-culture chromatogram.

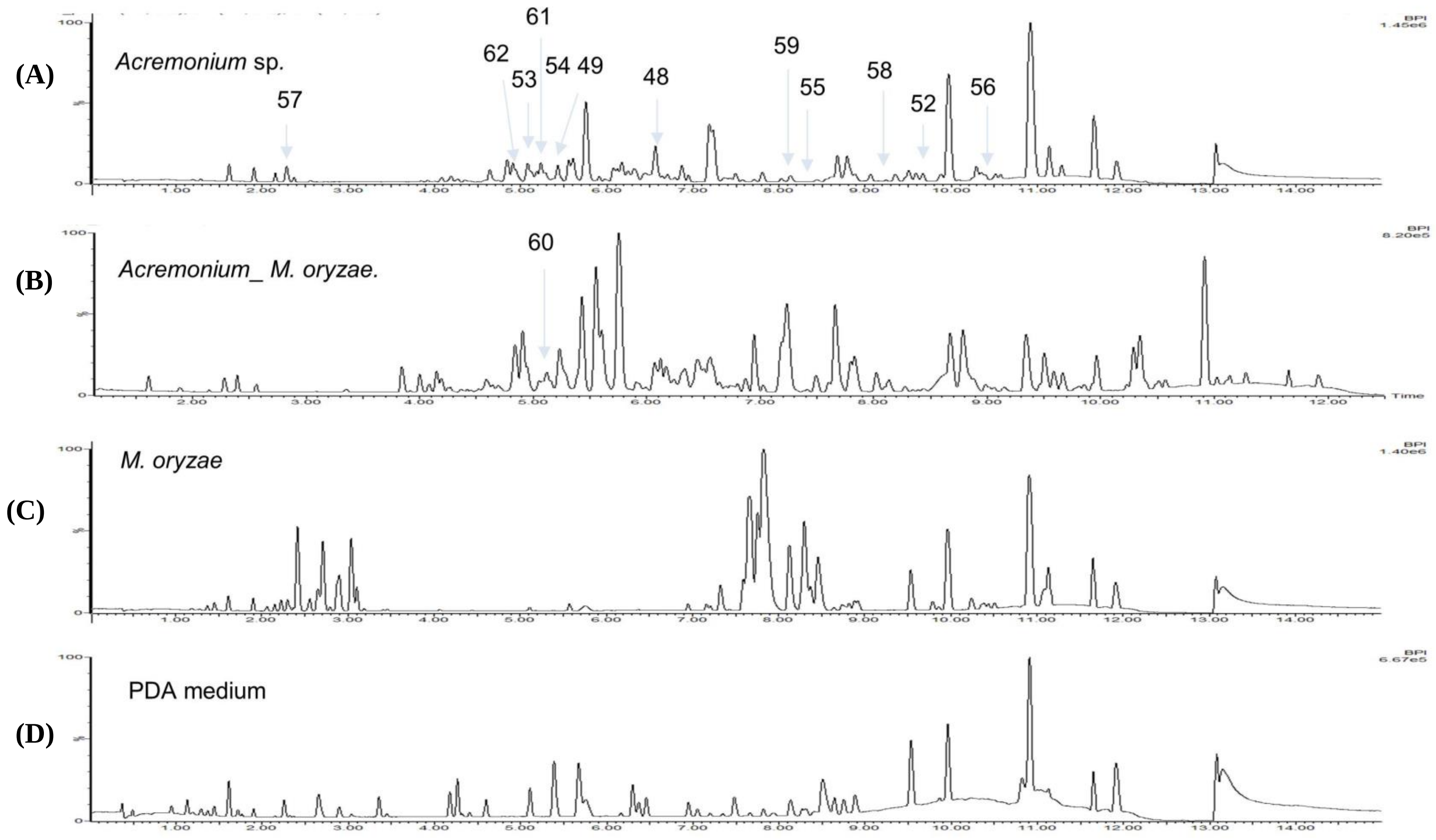

Supplementary Figure 21. Base peak chromatograms of whole agar extracts of *Acremonium* sp. mono-culture **(A)**, *M. oryzae* mono-culture **(C)** and their co-culture in PDA medium **(B)** showing putatively identified peak ions. Chromatogram of blank PDA medium is also displayed **(D)**. Numbers correspond to metabolites listed in dereplication table (**Supplementary Table 2**). Identical peak ions identified in both marine-adapted fungal isolate and co-culture were annotated in chromatogram of marine-adapted fungus only. Peak ions induced in co-culture are additionally annotated in co-culture chromatogram.

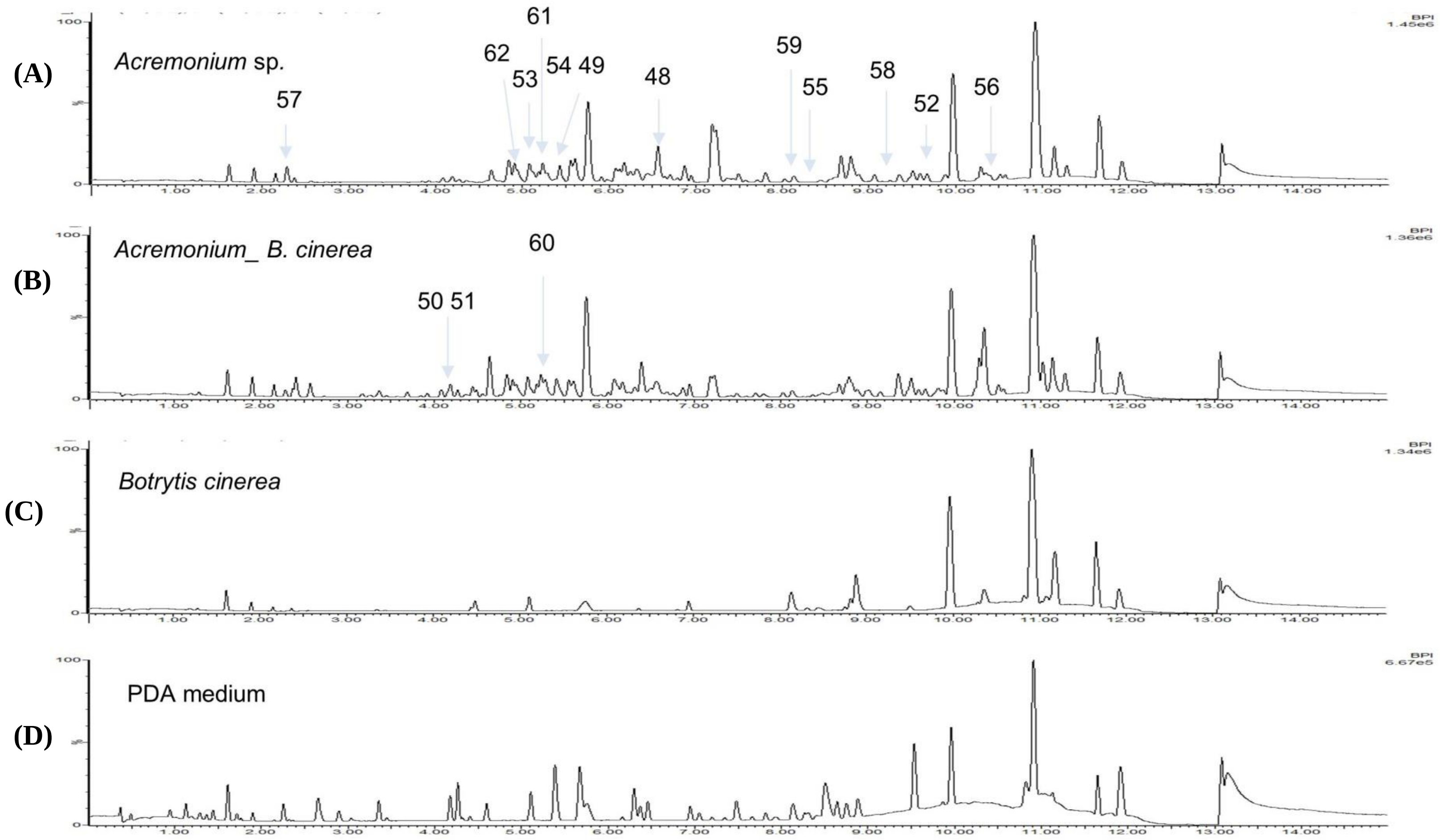

Supplementary Figure 22. Base peak chromatograms of whole agar extracts of *Acremonium* sp. mono-culture **(A)**, *B. cinerea* mono-culture **(C),** and their co-culture in PDA medium **(B)** showing putatively identified peak ions. Chromatogram of blank PDA medium is also displayed **(D)**. Numbers correspond to metabolites listed in dereplication table (**Supplementary Table 2**). Identical peak ions identified in both marine-adapted fungal isolate and co-culture were annotated in chromatogram of marine-adapted fungus only. Peak ions induced in co-culture are additionally annotated in co-culture chromatogram.

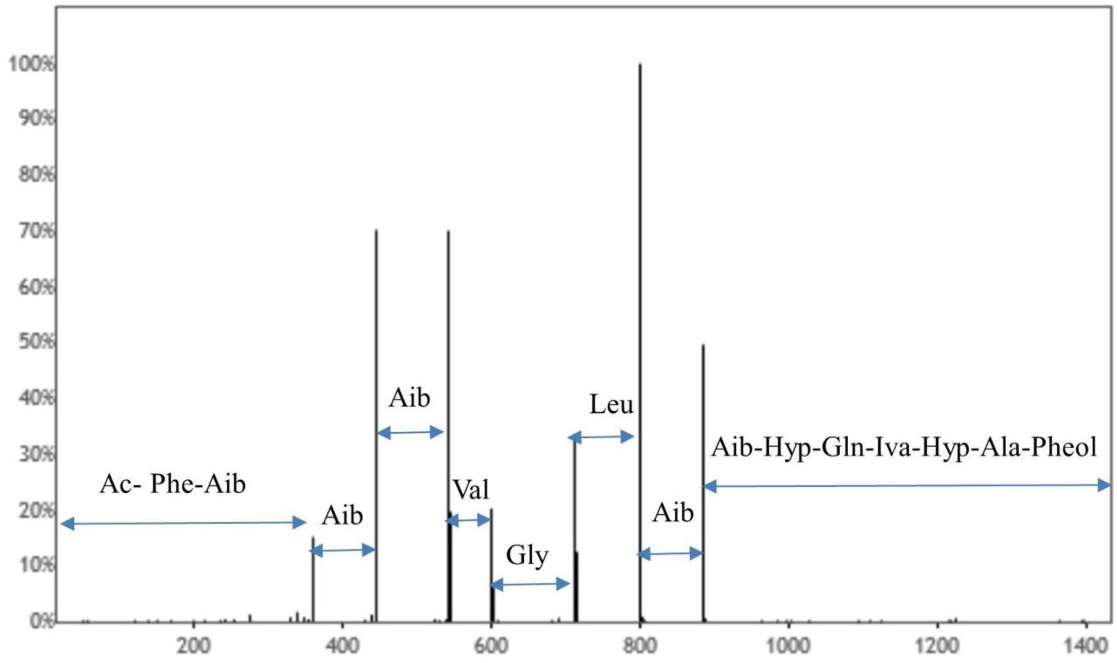

Supplementary Figure 23. Annotated MS/MS spectrum of emerimicin IV (*m/z* $[M+H]^+$ 1573.8994 based on MS/MS spectrum acquired in positive mode from 50 to 1600 Da). Each fragment is equivalent to an amino acid. Phe - phenylalanine, Aib - alpha-aminoisobutyric acid, Val - valine, Gly - glycine, Leu - leucine, Hyp - hydroxyproline, Gln - glutamine, Ala - alanine, Pheol - phenylalaninol

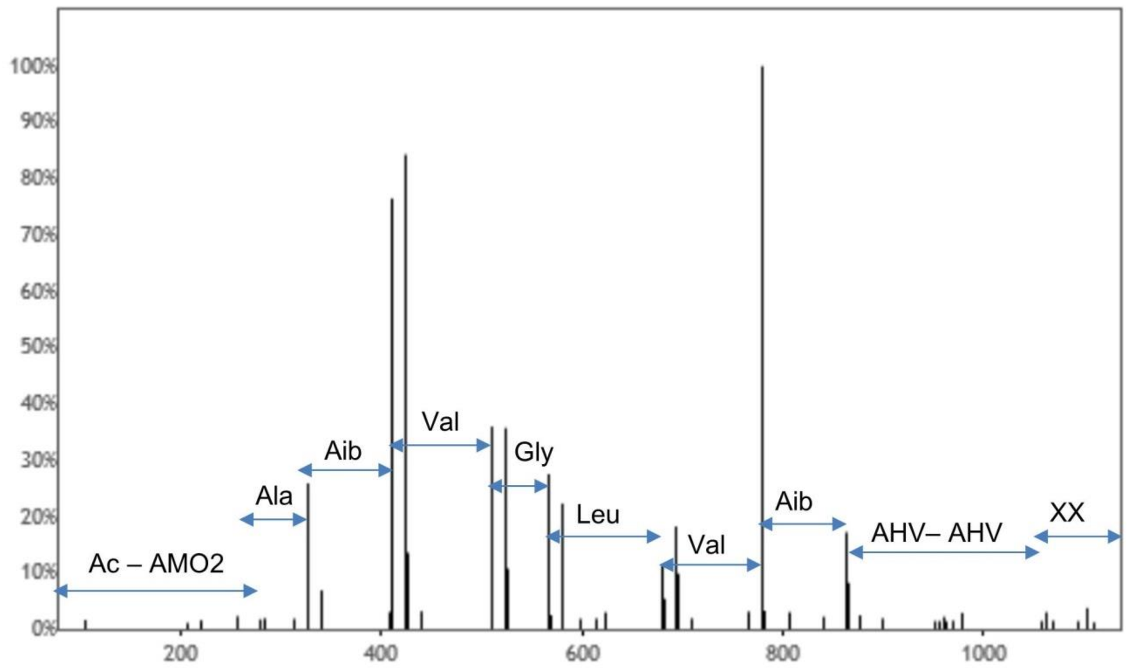

Supplementary Figure 24. Annotated MS/MS spectrum of a putatively new peptide of the Emerimicin family (*m/z* $[M+H]^+$ 1204.7736 based on MS/MS spectrum acquired in positive mode from 50 to 1600 Da). XX- unknown amino acid with *m/z* $[M+H]^+$ 110.0354 corresponding to a molecular formula of $C_4H_4N_3O$, AHV- 3-amino-2-hydroxyvaline, Aib- alpha- aminoisobutyric acid, Val- valine, Leu-leucine, Gly- glycine, Ala- alanine, AMO2- 2 amino- N,4-dimethyl-8-oxodecanoic acid

1.2 Supplementary Tables

Supplementary Table 1. Identification of 123 strains isolated from Windebyer Noor (Nov, 2015) according to sequence comparison with the NCBI nucleotide database using BLASTn (incl. respective assigned Genbank accession numbers). The 3 first BLAST hits incl. respective accession numbers are given. WO: twigs, F: Foam, SC: driftwood scrapings, L: leaves, WA: water, SE: sediment, SD: seeds. Media: GPY: Glucose Peptone Yeast, PCA: Potato Carrot agar, WSP: modified Wickerham medium and HS: Hastings medium.

No.	Strain I.D.	Sample material	Medium	Ampli-con	Sequence length	Blast result	% simi-larity	Accession number	Fungal Order	Accession number
**1**	31WA1A	WO	GPY	ITS	416	*Pythium* sp. isolate Pc36-1C	100	KY785405.1	Pythiales	MH791195
						Pythium sp. isolate Pc70-1W		KY785393.1		
						Pythium rhizo-oryzae strain 2015-F-272		KU751877.1		
**2**	12WE1B	WO	PCA	ITS	236	*Acremonium breve* culture MUT<ITA>:4975	96	KY081463.1	Hypocreales	MH791196
						Acremonium sp. L17_BA	95	KX098313.1		
						Acremonium sp. AT10	95	KT377204.1		
**3**	25WA1B	WO	HS	ITS	439	Uncultured fungus clone ITS_S1_clon18	99	HQ873336.1	Glomerellales	MH791197
						Plectosphaerella sp. 198 OA-2013		JX535155.1		
						Plectosphaerella sp. 193 OA-2013		JX535151.1		
**4**	54WS1D	WO	PCA	ITS	400	*Cladosporium* sp. strain Vp-1	99	KY305291.1	Capnodiales	MH791198
						Cladosporium herbarum		LC317547.1		
						Davidiella tassiana clone 104o		EU343348.1		
**5**	30H1A	WA	HS	ITS	456	*Microdochium phragmitis* CBS 285.71	100	NR_132916.1	Xylariales	MH791199
						Microdochium phragmitis strain CBS 423.78		KP859012.1		
						Uncultured Microdochium clone IIN5F29		FJ197870.1		
**6**	33WB1C	WO	WSP	ITS	267	*Cladosporium* sp. strain NR47	100	MG845222.1	Capnodiales	MH791200
						Cladosporium sp. strain NR38		MG845218.1		
						Cladosporium sp. strain NR37		MG845217.1		
**7**	35WB1C	WO	HS	ITS	496	*Plectosphaerella oligotrophica* strain AUMC 11010	99	KX446769.1	Glomerellales	MH791249
						Uncultured *Plectosphaerella*, clone 09D50C59 (MOTU71)		HG936781.1		
						Uncultured *Plectosphaerella*, clone 09D10C71 (MOTU71)		HG936780.1		
**8**	62WD1D	WO	PCA	18S	1526	*Plectosphaerella* sp. MH727	99	FJ430715.1	Glomerellales	MH791178
						Plectosphaerella sp. CCF3811		FJ430714.1		
						Fungal sp. isolate nussu_31		KT714157.1		
**9**	56WS1D	SC	PCA	ITS	351	*Aureobasidium* sp. strain NR88	99	MG845226.1	Dothideales	MH791201
						Aureobasidium pullulans culture MUT<ITA>:2186		MG807338.1		
						Aureobasidium pullulans strain YLAC-4		KY552634.1		
**10**	55WS1D	SC	PCA	ITS	312	*Phoma eupyrena* isolate F260	100	LT821518.1	Pleosporales	MH791202
						Phoma eupyrena isolate F224		LT821490.1		
						Phoma eupyrena isolate F223		LT821489.1		
**11**	8WE1B	WO	PCA	ITS	488	*Cladosporium cladosporioides* M47	100	MG748632.1	Capnodiales	MH791203
						Cladosporium cladosporioides		MG572365.1		
						Cladosporium pseudocladosporioides AY812		MG250413.1		

**12**	80F1C	F	WSP	ITS	454	*Penicillium bialowiezense* strain KAS5860<br>*Penicillium brevicompactum* strain 82<br>*Penicillium brevicompactum* strain IHBF 2254	100	KY469051.1<br>KY401133.1<br>MF326601.1	Eurotiales	MH791250
**13**	78F1C	F	WSP	-	-	-			-	
**14**	66L1B	L	HS	ITS	403	*Cladosporium* sp. strain NR38<br>*Cladosporium* sp. strain NR37<br>*Cladosporium* sp. strain NR32	100	MG845218.1<br>MG845217.1<br>MG845214.1	Capnodiales	MH791204
**15**	52WS1D	SC	HS	ITS	401	*Cladosporium* sp. strain NR3*8*<br>*Cladosporium* sp. strain NR37<br>*Cladosporium* sp. strain NR32	99	MG845218.1<br>MG845217.1<br>MG845214.1	Capnodiales	MH791205
**16**	64L1C	L	HS	ITS	327	*Cladosporium* sp. strain NR47<br>*Cladosporium* sp. strain NR38<br>*Cladosporium* sp. strain NR37	100	MG845222.1<br>MG845218.1<br>MG845217.1	Capnodiales	MH791206
**17**	22WA1B	WO	GPY	18S	763	*Chordomyces antarcticum* strain V213<br>*Chordomyces antarcticum* strain A135<br>*Chordomyces antarcticum* strain A134	99	KJ443101.1<br>KJ443060.1<br>KJ443059.1	Glomerellales	MH791189
**18**	61WD1D	WO	HS	ITS	459	*Plectosphaerella cucumerina* isolate E-429<br>*Plectosphaerella cucumerina* isolate E-371<br>*Plectosphaerella cucumerina* isolate E-370	98	KY582156.1<br>KY582117.1<br>KY582116.1	Glomerellales	MH791251
**19**	84F1B	F	HS	18S	1547	*Penicillium expansum* strain HDJZ-ZWM-17<br>*Penicillium* sp. strain OUCMDZ-4754<br>*Penicillium* sp. 52	100	GU227344.1<br>MG736296.1<br>KU350746.1	Eurotiales	MH791177
**20**	95S1A	SE	GPY	ITS	317	*Acremonium* sp. 195 OA-2013<br>*Acremonium* sp. 86 OA-2013<br>*Acremonium* sp. 28 OA-2013	99	JX535153.1<br>JX535094.1<br>JX535052.1	Hypocreales	MH791207
**21**	29H1A	WA	GPY	18S	1539	*Pyrenochaeta nobilis* strain CBS 407.76<br>*Pyrenochaeta nobilis* strain CBS 407.76<br>*Cucurbitaria berberidis* strain	98	MF795792.1<br>DQ898287.1<br>U42481.1	Pleosporales	MH791187
**22**	19WE1C	WO	PCA	ITS	249	*Cladosporium* sp. strain NR47<br>*Cladosporium* sp. strain NR38<br>*Cladosporium* sp. strain NR37	100	MG845222.1<br>MG845218.1<br>MG845217.1	Capnodiales	MH791208
**23**	23WA1B	WO	PCA	ITS	494	*Cladosporium* sp. isolate 39_40_43<br>*Cladosporium asperulatum* strain KG_10<br>*Cladosporium* sp. isolate 1066	96	KX034368.1<br>MG647842.1<br>KT826661.1	Capnodiales	MH791208
**24**	26WA1B	WO	HS	ITS	283	*Penicillium bialowiezense* strain KAS5860<br>*Penicillium brevicompactum* strain 82<br>*Penicillium brevicompactum* strain IHBF 2254	99	KY469051.1<br>KY401133.1<br>MF326601.1	Eurotiales	MH791210
**25**	9WE1B	WO	PCA	18S	801	*Penicillium* sp. strain OUCMDZ-4754<br>*Penicillium spinulosum* strain 092<br>*Penicillium spinulosum* strain 087	99	MG736296.1<br>MF072608.1<br>MF072607.1	Eurotiales	MH791173
**26**	13WE1B	WO	PCA	18S	851	*Penicillium* sp. strain OUCMDZ-4754<br>*Penicillium* sp. strain NSY15<br>*Penicillium* sp. 52	100	MG736296.1<br>KX906964.1<br>KU350746.1	Eurotiales	MH791194
**27**	39.27WA1B	WO	WSP	ITS	446	*Acremonium* sp. 195 OA-2013<br>*Acremonium* sp. 86 OA-2013<br>*Acremonium* sp. 28 OA-2013	99	JX535153.1<br>JX535094.1<br>JX535052.1	Hypocreales	MH791252
**28**	48.21WA1B	WO	GPY	18S	1528	*Cladosporium sp. Y19-1*<br>*Cladosporium* sp. MF528<br>*Cladosporium* sp. MF456	99	KP872512.1<br>KM096331.1<br>KM096275.1	Capnodiales	MH791181
**29**	33.81F1B	F	HS	ITS	352	*Fusarium culmorum* isolate 5761<br>*Fusarium culmorum* isolate 5763	99	MG736106.1<br>MG736104.1	Eurotiales	MH791277

**30**	63WD1A	WO	GPY	ITS	303	*Fusarium culmorum* isolate 9712<br>*Fusarium graminearum* strain FSHG<br>*Fusarium graminearum* isolate WF1	100	MG274304.1<br>KU939070.1<br>KY985465.1	Hypocreales	MH791211
**31**	7.6WE1B	WO	HS	ITS	316	*Fusarium* sp. isolate 5643<br>*Fusarium culmorum* isolate 5761<br>*Fusarium culmorum* isolate 5763	99	MG736188.1<br>MG736106.1<br>MG736104.1	Hypocreales	MH791212
**32**	42.99S1B	SE	WSP	ITS	454	*Fusarium culmorum* isolate 9712<br>*Penicillium bialowiezense* strain KAS5860<br>*Penicillium brevicompactum* strain 82	100	MG274304.1<br>KY469051.1<br>KY401133.1	Eurotiales	MH791253
**33**	2.58WC1A	WO	WSP	ITS	223	*Penicillium brevicompactum* strain IHBF 2254<br>Uncultured endophytic fungus clone 13-11-11<br>*Epicoccum nigrum* isolate MFLUCC 17-1162	99	MF326601.1<br>EF505272.1<br>MG807029.1	Hypocreales	MH791278
**34**	56.47WS1A	SC	WSP	ITS	313	*Epicoccum nigrum* isolate MFLUCC 17-1153<br>*Epicoccum nigrum* isolate MFLUCC 17-1162<br>*Epicoccum nigrum* isolate MFLUCC 17-1153	100	MG807028.1<br>MG807029.1<br>MG807028.1	Hypocreales	MH791213
**35**	53.50WS1B	SC	HS	ITS	311	*Epicoccum nigrum* isolate MFLUCC 17-1214<br>*Fusarium graminearum* strain FSHG<br>*Fusarium graminearum* isolate WF1	100	MG807027.1<br>KU939070.1<br>KY985465.1	Hypocreales	MH791214
**36**	43.100S1B	SE	WSP	ITS	447	*Fusarium* sp. isolate 5643<br>*Sarocladium* sp. strain MAC1<br>*Sarocladium strictum* isolate VGSS16-1	99	MG736188.1<br>MG649462.1<br>MF671820.1	Hypocreales	MH791254
**37**	10.45WS1A	SC	PCA	ITS	321	*Sarocladium strictum* isolate UWR_109<br>*Fusarium* sp. isolate 5662<br>*Fusarium* sp. isolate 5664	100	KY465763.1<br>MG736185.1<br>MG736184.1	Hypocreales	MH791279
**38**	4.32WB1C	WO	WSP	ITS	404	*Fusarium culmorum* isolate 5761<br>*Microdochium bolleyi* strain RFA7P<br>*Microdochium bolleyi* strain Q6-3	99	MG736106.1<br>KY305060.1<br>KY365584.1	Xylariales	MH791255
**39**	61.60WC1B	WO	WSP	ITS	261	*Microdochium bolleyi* strain NQ5-4<br>*Acremonium* sp. 195 OA-2013<br>*Acremonium* sp. 86 OA-2013	99	KY365578.1<br>JX535153.1<br>JX535094.1	Hypocreales	MH791215
**40**	57.47WS1A	SC	PCA	ITS	270	*Acremonium* sp. 28 OA-2013<br>*Alternaria alternata* isolate ET57<br>*Alternaria alternata* isolate ET58	100	JX535052.1<br>KY774665.1<br>KY774664.1	Pleosporales	MH791216
**41**	46.51WS1C	SC	WSP	ITS	305	*Alternaria alternata* strain Alt008<br>*Epicoccum nigrum* isolate MFLUCC 17-1162<br>*Epicoccum nigrum* isolate MFLUCC 17-1153	100	MG827243.1<br>MG807029.1<br>MG807028.1	Pleosporales	MH791217
**42**	12.1WE1B	WO	GPY	ITS	305	*Epicoccum nigrum* isolate MFLUCC 17-1214<br>*Fusarium graminearum* isolate WF1<br>*Fusarium* sp. isolate 5643	100	MG807027.1<br>KY985465.1<br>MG736188.1	Hypocreales	MH791218
**43**	29.36WB1C	WO	HS	ITS	331	*Fusarium* sp. isolate 5662<br>Hypocreales sp. S68<br>Hypocreales sp. SE51	98<br>97	MG736185.1<br>HQ596921.1<br>HQ596914.1	Hypocreales	MH791219
**44**	6.96S1C	SE	GPY	ITS	376	*Acremonium breve* culture MUT<ITA>:4975<br>*Pyrenochaeta cava* strain SO1_T23_L3B<br>*Pyrenochaeta cava* isolate HL	96<br>100	KY081463.1<br>KY367517.1<br>KR909135.1	Pleosporales	MH791292
**45**	27.87F1B	F	GPY	18S	1538	*Pyrenochaeta cava* Gall_31<br>*Penicillium brevicompactum* strain ALI 318<br>*Penicillium brevicompactum* strain CB10	98	KT823796.1<br>AF548082.1<br>KM222211.1	Eurotiales	MH791188
**46**	38.42FS	SD	PCA	18S	1533	*Penicillium brevicompactum* strain ALI 319<br>*Penicillium freii* (IBT 3464)<br>Fungal sp. ZJ50	97	AF548083.1<br>AJ005446.1<br>KT582238.1	Eurotiales	MH791182

**47**	36.97F1C	F	PCA	18S	1122	*Penicillium* sp. MF523<br>*Penicillium* sp. FA6-2<br>*Penicillium* sp. FA9<br>*Penicillium brevicompactum* isolate PenC	98	KM096327.1<br>KF776918.1<br>KF776917.1<br>FJ717699.1	Eurotiales	MH791183
**48**	25.88F1C	F	WSP	ITS	308	*Emericellopsis* sp. s012<br>*Acremonium* sp.195OA-2013<br>*Acremonium* sp.86OA-2013	95	HQ649988.1<br>JX535153.1<br>JX535094.1	Capnodiales	MH791280
**49**	11.45WS1A	SC	WSP	ITS	305	*Fusarium culmorum* isolate 5761<br>*Fusarium culmorum* isolate 5763<br>*Fusarium culmorum* isolate 9712	100	MG736106.1<br>MG736104.1<br>MG274304.1	Hypocreales	MH791220
**50**	WA1D3	WO	HS	ITS	324	*Acremonium* sp.195OA-2013<br>*Acremonium* sp. 86OA-2013<br>*Acremonium* sp.28OA-2013	99	JX535153.1<br>JX535094.1<br>JX535052.1	Hypocreales	MH791221
**51**	47.57WS1D	SC	HS	ITS	315	*Cladosporium* sp. strain NR47<br>*Cladosporium* sp. strain NR38<br>*Cladosporium* sp. strain NR37	100	MG845222.1<br>MG845218.1<br>MG845217.1	Capnodiales	MH791222
**52**	41.20WA1B	WO	WSP	ITS	313	*Cosmospora stegonsporii* strain UASWS1262<br>*Cosmospora stegonsporii* strain A.R.4385<br>*Nectria flavoviridis* strain IMI338173	99	KP114076.1<br>KC291718.1<br>KC291747.1	Hypocreales	MH791291
**53**	44.38FS	SD	GPY	ITS	266	*Cladosporium* sp. strain NR47<br>*Cladosporium* sp. strain NR38<br>*Cladosporium*sp.strain NR37	100	MG845222.1<br>MG845218.1<br>MG845217.1	Capnodiales	MH791223
**54**	49.49WS1B	SC	GPY	ITS	313	*Phoma eupyrena* isolateF260<br>*Phoma eupyrena* isolateF224<br>*Phoma eupyrena* isolate F223	100	LT821518.1<br>LT821490.1<br>LT821489.1	Mytilinidiales	MH791224
**55**	9.59WC1B	WO	PCA	ITS	308	*Fusarium avenaceum* isolate F70<br>*Fusarium avenaceum* isolate F39<br>*Fusarium avenaceum* isolate F33	100	LT821457.1<br>LT821425.1<br>LT821419.1	Hypocreales	MH791225
**56**	45.53WS1C	SC	GPY	ITS	457	*Alternaria alternata* isolate ET57<br>*Alternaria alternata* isolate ET58<br>*Alternaria alternata* strain Alt008	100	KY774665.1<br>KY774664.1<br>MG827243.1	Pleosporales	MH791226
**57**	13.1WE1B	WO	GPY	ITS	308	*Fusarium graminearum* isolate WF1<br>*Fusarium* sp. isolate 5643<br>*Fusarium* sp. isolate 5662	100	KY985465.1<br>MG736188.1<br>MG736185.1	Hypocreales	MH791227
**58**	28.65L1C	L	GPY	ITS	344	*Cladosporium* sp. strain NR47<br>*Cladosporium* sp. strain NR38<br>*Cladosporium*sp.strain NR37	99	MG845222.1<br>MG845218.1<br>MG845217.1	Capnodiales	MH791228
**59**	40.24WA1B	WO	HS	ITS	383	*Microdochium phragmitis* CBS285.71<br>*Microdochium phragmitis* strain CBS423.78<br>*Monographella* sp.GW_OTU50	99	NR_132916.1<br>KP859012.1<br>KP714578.1	Xylariales	MH791229
**60**	WD1D	WO	PCA	ITS	295	*Plectosphaerella cucumerina isolate NIHHS403*<br>*Fungal sp.isolate ZY-58*<br>*Plectosphaerella oligotrophica* OA2S1-48	100	KY555004.1<br>KY040255.1<br>MG214595.1	Glomerellales	MH791256
**61**	WB1D	WO	GPY	ITS	381	*Lentitheciaceae* sp. MUT4420<br>*Pleosporales* sp. CBS193.87<br>*Phoma*sp. CJL-2014 strainRc-R-42	94	KF636770.1<br>KY940785.1<br>KJ542231.1	Pleosporales	MH791230
**62**	WA1C	WO	WSP	ITS	320	*Acremonium* sp.195OA-2013<br>*Acremonium* sp. 86OA-2013<br>*Acremonium* sp.28OA-2013	99	JX535153.1<br>JX535094.1<br>JX535052.1	Hypocreales	MH791231
**63**	58WC1A	WO	WSP	ITS	413	*Epicoccum nigrum* isolate MFLUCC17-1162<br>*Epicoccum nigrum* isolate MFLUCC17-1153<br>*Epicoccum nigrum* isolate MFLUCC17-1214	100	MG807029.1<br>MG807028.1<br>MG807027.1	Pleosporales	MH791257

**64**	S1DA	SE	WSP	ITS	507	Fungal sp. strain OTU53<br>Helotiales sp. 203OA-2013<br>Helotiales sp.104OA-2013	99	KT923227.1<br>JX507714.1<br>JX507688.1	Helotiales	MH791258
**65**	1EWE2	WO	WSP	ITS	453	*Cladosporium* sp. isolate UCD160913G3_16UCDE10<br>*Cladosporium* sp. isolatePCR39<br>*Cladosporium* sp. isolatePCR35	100	MG686514.1<br>KY436105.1<br>KY436103.1	Capnodiales	MH791267
**66**	86F1C	F	WSP	ITS	482	*Cladosporium cladosporioides* isolate M47<br>*Cladosporium cladosporioides* isolate stone isolate15<br>*Cladosporium pseudocladosporioides* strain AY812	100	MG748632.1<br>MG572365.1<br>MG250413.1	Capnodiales	MH791259
**67**	S1CB	SE	GPY	18s	1478	*Pyrenochaeta nobilis* strain CBS407.76<br>*Pyrenochaeta nobilis* strain CBS407.76<br>*Pyrenochaeta* sp. 14009	98	MF795792.1<br>DQ898287.1<br>EU710832.1	Pleosporales	MH791174
**68**	WE1CA	WO	WSP	ITS	176	*Phoma* sp. MUT5442<br>*Phoma* sp.MUT5380<br>*Phoma* sp. MUT5378	100	KU314970.1<br>KU314957.1<br>KU314955.1	Pleosporales	MH791232
**69**	S1DB	SE	HS	ITS	265	*Plenodomus influorescens* strain CBS143.84<br>*Plenodomus lindquistii* strain P47<br>*Plenodomus lindquistii* strain P46	100	JF740228.1<br>KU361380.1<br>KU361379.1	Pleosporales	MH791233
**70**	5.32WB1C	WO	WSP	ITS	444	*Penicillium bialowiezense* strain KAS5860<br>*Penicillium brevicompactum* strain 82<br>*Penicillium brevicompactum* strain IHBF2254	100	KY469051.1<br>KY401133.1<br>MF326601.1	Eurotiales	MH791260
**71**	59.47WS1A	SC	PCA	18S	965	*Acremonium* sp. CSPC Betsoh<br>*Plectosphaerella* sp. MH727<br>*Plectosphaerella* sp. CCF3811	99	KJ867227.1<br>FJ430715.1<br>FJ430714.1	Hypocreales	MH791179
**72**	7F1B	F	HS	ITS	326	*Penicillium* sp. CCN25<br>*Penicillium brevicompactum* strain KAS5871<br>*Penicillium brevicompactum* strain KAS5811	99	DQ993646.1<br>KY469055.1<br>KY469040.1	Eurotiales	MH791234
**73**	3.75F1C	F	GPY	18S	1529	*Penicillium expansum*strain HDJZ-ZWM-17<br>*Penicillium* sp. strain OUCMDZ-4754<br>*Penicillium* sp.52	100	GU227344.1<br>MG736296.1<br>KU350746.1	Eurotiales	MH791185
**74**	30.36WB1D	WO	HS	ITS	325	*Penicillium bialowiezense* strain KAS5860<br>*Penicillium brevicompactum* strain 82<br>*Penicillium brevicompactum* strain IHBF2254	100	KY469051.1<br>KY401133.1<br>MF326601.1	Eurotiales	MH791235
**75**	31.68F1B	F	HS	ITS	324	*Penicillium bialowiezense* strain KAS5860<br>*Penicillium brevicompactum* strain 82<br>*Penicillium brevicompactum* strain IHBF2254	100	KY469051.1<br>KY401133.1<br>MF326601.1	Eurotiales	MH791236
**76**	50.82F1B	F	HS	ITS	327	*Penicillium brevicompactum* strain KAS5871<br>*Penicillium brevicompactum* strain KAS5811<br>*Penicillium brevicompactum* strain KAS5773	100	KY469055.1<br>KY469040.1<br>KY469035.1	Eurotiales	MH791237
**77**	H1BR	WA	HS	ITS	327	*Penicillium brevicompactum* strain KAS5854<br>*Penicillium brevicompactum* strain KAS5812<br>*Penicillium brevicompactum* strain KAS5776	100	KY469047.1<br>KY469041.1<br>KY469037.1	Eurotiales	MH791238
**78**	F1D	F	GPY	ITS	392	*Penicillium brevicompactum* strain MERVA5<br>*Penicillium* sp.isolate UCD160901G3_16UCDE1<br>*Penicillium brevicompactum* strainKG_6	99	MF503895.1<br>MG686505.1<br>MG647838.1	Eurotiales	MH791281
**79**	37.92F1B	F	PCA	ITS	446	*Penicillium brevicompactum* strain 82<br>*Penicillium brevicompactum* strain IHBF2254<br>*Penicillium* sp. isolate BR692	100	KY401133.1<br>MF326601.1<br>KY438237.1	Eurotiales	MH791261
**80**	WA1D	WO	PCA	ITS	328	*Penicillium brevicompactum* strain KAS5854<br>*Penicillium brevicompactum* strain KAS5812<br>*Penicillium brevicompactum* strain KAS5776	100	KY469047.1<br>KY469041.1<br>KY469037.1	Eurotiales	MH791239

**81**	62.72F1A	F	PCA	ITS	364	*Penicillium brevicompactum* strain 82	100	KY401133.1	Eurotiales	MH791282
						Penicillium brevicompactum strain IHBF2254		MF326601.1		
						Penicillium sp. isolate BR692		KY438237.1		
**82**	3WE1B	WO	GPY	18S	553	Hypocreomycetidae sp. ST-2017a	98	KY882037.1	Hypocreales	MH791186
						Gliocladium cibotii isolate gcy1		KY315573.1		
						Acremonium furcatum strain AY858		MG250388.1		
**83**	37RWE2	WO	GPY	ITS	484	*Phialemonium* sp. Gall_27	100	KT823792.1	Sordariales	MH791268
						Phialemonium dimorphosporum strain 2015-F-034		KU751865.1		
						Phialemonium sp. T004-F2-2		LC109299.1		
**84**	5EWS2	SC	PCA	ITS	399	Fungal endophyte isolate 15	96	KU179250.1	Pleosporales	MH791242
						Phoma sp. CJL-2014 strainRc-R-42	95	KJ542231.1		
						Byssothecium obiones isolate CM1CS2C1	94	KX263807.1		
**85**	33XWA2	WO	GPY	18S	729	*Emericellopsis minima* strain CBS871.68	99	KC987212.1	Hypocreales	MH791184
						Acremonium sp. TVG-S004-0211		KU145487.1		
						Acremonium sp. Y36-2		KP872531.1		
**86**	35XWA2	WO	PCA	ITS	376	*Emericellopsis* sp. OUCMBI101058	97	HQ914819.1	Hypocreales	MH791290
						Fungal sp.strainTH31		KY607763.1		
						Uncultured *Emericellopsis*, clone10J50C67(MOTU75)		HG936806.1		
**87**	14PWF2	WO	GPY	ITS	303	*Pseudohalonectria lignicola* strain M95	96	JX134679.1	Magnaporthal<br>es	MH791285
						Phomopsis sp. ONH-2015 strain MATB4	93	KM510387.1		
						Macrophomina phaseolina strain JD-CP2	93	JQ954870.1		
**88**	2EWE2	WO	GPY	18S	1519	Lindgomycetaceae sp. strain KF970	99	KY362378.1	Pleosporales	MH791191
						Lindgomycetaceae sp. KF970		KM096176.1		
						Uncultured fungus: A_3_16		AB534319.1		
**89**	10PL2	L	GPY	ITS	250	*Penicillium porphyreum* CBS382.64	100	NR_153224.1	Eurotiales	MH791269
						Penicillium lagena CBS185.65		NR_153223.1		
						Penicillium sp. strain SYPF7919		MF588872.1		
**90**	17PH2	WA	GPY	18S	1531	*Aspergillus fumigatus* strainYuZhu2	100	KU512836.1	Eurotiales	MH791192
						Aspergillus fumigatus strain TMS-26		KJ746594.1		
						Aspergillus fumigatus strain WL002		KJ528402.1		
**91**	24PWA2	WO	WSP	ITS	459	*Leptosphaeria* sp. isolate DEPMDZ-9	99	KU901551.1	Pleosporales	MH791286
						Leptosphaeria sp. aurim1184		DQ093683.1		
						Pleosporales sp. bc_gw_9967a		KF428332.1		
**92**	18.74F1C	F	WSP	-		-			-	
**93**	32XWF2	WO	GPY	ITS	544	Cadophora *luteo-olivacea* isolate Cadoph_LO2	100	MG944391.1	Helotiales	MH796079
						Cadophora luteo-olivacea isolate Cadoph_LO1		MG944390.1		
						Cadophora luteo-olivacea culture MUT<ITA>:2817		MG813217.1		
**94**	26XWS2	SC	GPY	ITS	460	*Fungal endophyte isolate 15*	97	KU179250.1	Pleosporales	MH791270
						Phoma sp.CJL-2014 strain Rc-R-42	96	KJ542231.1		
						Byssothecium obiones isolate CM1CS2C1	95	KX263807.1		
**95**	HBB	WA	HS	ITS	389	*Penicillium* sp. DUCC7308	100	KC291136.1	Eurotiales	MH791283
						Penicillium carneum strain CBS468.65		JN097809.1		
						Penicillium carneum CBS112297		NR_111551.1		
**96**	3EWA2	WO	WSP	ITS	422	*Gibellulopsis nigrescens* isolate LG1401_GL11A	100	KX359602.1	Glomerellales	MH791271
						Gibellulopsis nigrescens culture-collection MUT<ITA>:1664		KU935672.1		
						Gibellulopsis sp.MYf203		KX079890.1		
**97**	16PWC2	WO	GPY	18S	1536	*Penicillium chrysogenum* strain	99	KX580630.1	Eurotiales	MH791193
						Fungal sp. ZJ48		KT582272.1		
						Fungal sp. ZJ34		KT582268.1		
**98**	H1A_C	WA	GPY	18S	1017	*Penicillium brevicompactum* strain ALI318	99	AF548082.1	Eurotiales	MH791175

						Penicillium brevicompactum strain CB10		KM222211.1		
						Penicillium brevicompactum strain CB9		KM222210.1		
**99**	13PWB2	WO	HS	ITS	457	*Plectosphaerella cucumerina* isolate E-429	99	KY582156.1	Xylariales	MH791272
						Plectosphaerella cucumerina isolate E-371		KY582117.1		
						Plectosphaerella cucumerina isolate E-370		KY582116.1		
**100**	18PWS2	SC	GPY	ITS	301	Fungal endophyte isolate 15	97	KU179250.1	Pleosporales	MH791243
						Byssothecium obiones isolate CM1CS2C1	96	KX263807.1		
						Pleosporales sp. s092	96	HQ649784.1		
**101**	WA1D2	WO	PCA	ITS	327	*Penicillium* sp. isolate UCD160901G3_16UCDE1	100	MG686505.1	Eurotiales	MH791240
						Penicillium brevicompactum strain KG_6		MG647838.1		
						Penicillium brevicompactum strain Bsa4SNA3		KY558616.1		
**102**	9ES2	SE	PCA	ITS	341	Fungal endophyte isolate 15	96	KU179250.1	Pleosporales	MH791244
						Byssothecium obiones isolate CM1CS2C1	94	KX263807.1		
						Pleosporales sp. s092	94	HQ649784.1		
**103**	4EWS2	SC	WSP	ITS	241	*Acremonium* sp. 195OA-2013	99	JX535153.1	Hypocreales	MH791245
						Acremonium sp. 86OA-2013		JX535094.1		
						Acremonium sp. 28OA-2013		JX535052.1		
**104**	27XWS2	SC	WSP	ITS	459	Fungal endophyte isolate 15	95	KU179250.1	Pleosporales	MH791273
						Phoma sp. CJL-2014 strain Rc-R-42		KJ542231.1		
						Byssothecium obiones isolate CM1CS2C1		KX263807.1		
**105**	L1D_A	L	PCA	ITS	322	*Cladosporium cladosporioides*	100	MG836709.1	Capnodiales	MH791241
						Cladosporium cladosporioides isolate SR779-ENO1		KY964059.1		
						Cladosporium sp. isolate C7-10		MG818941.1		
**106**	39RWC2	WO	WSP	ITS	494	*Phytophthora lacustris* strain CWM37902	100	JX272358.1	Perono-	MH791274
						Phytophthora lacustris isolate 250492		KT383052.1	sporales	
						Phytophthora lacustris isolate 250491		KT383051.1		
**107**	8ES2	SE	HS	ITS	503	Fungal endophyte isolate 15	97	KU179250.1	Pleosporales	MH791275
						Phoma sp. CJL-2014 strain Rc-R-42	96	KJ542231.1		
						Pleosporales sp. XJ7	96	KF143782.1		
**108**	41RWS2	SC	GPY	ITS	477	Fungal endophyte isolate 15	97	KU179250.1	Pleosporales	MH791287
						Phoma sp.CJL-2014 strainRc-R-42	96	KJ542231.1		
						Byssothecium obiones isolate CM1CS2C1	95	KX263807.1		
**109**	20PWS2	SC	WSP	ITS	484	*Hypoxylon fragiforme* isolate CBS206.31	99	KU684022.1	Xylariales	MH791288
						Uncultured *Hypoxylon* MOTU1		LC015715.1		
						Hypoxylon fragiforme voucher BIOUG24047-A11		KT695333.1		
**110**	22PWA2	WO	GPY	ITS	462	*Leptosphaeria* sp. isolate DEPMDZ-9	98	KU901551.1	Pleosporales	MH791276
						Leptosphaeria sp. aurim1184		DQ093683.1		
						Pleosporales sp. bc_gw_9967a		KF428332.1		
**111**	L1D	L	PCA	ITS	470	*Phaeosphaeriaceae* sp. OTU_081	92	HE998742.1	Pleosporales	MH791262
						Uncultured *Phaeosphaeria* clone FTrs21		KT759274.1		
						Uncultured fungus clone S276		FJ820763.1		
**112**	23PWA2	WO	PCA	ITS	442	*Leptosphaeria* sp. isolate DEPMDZ-9	98	KU901551.1	Pleosporales	MH791246
						Leptosphaeria sp. aurim1184		DQ093683.1		
						Pleosporales sp. bc_gw_9967a		KF428332.1		
**113**	25XWS2	SC	PCA	ITS	323	Fungal endophyte isolate 15	96	KU179250.1	Pleosporales	MH791247
						Byssothecium obiones isolate CM1CS2C1	96	KX263807.1		
						Pleosporales sp. s092	95	HQ649784.1		
**114**	28XWS2	SC	WSP	ITS	258	*Acremonium* sp. 195OA-2013	99	JX535153.1	Hypocreales	MH791248
						Acremonium sp. 86OA-2013		JX535094.1		
						Acremonium sp. 28OA-2013		JX535052.1		
**115**	FWD1C	WO	WSP	ITS	373	*Epicoccum nigrum* isolate 5341	99	MG736207.1	Pleosporales	MH791284

						Epicoccum nigrum strain: RH3		LC171699.1		
						Epicoccum nigrum strain		LC171695.1		
**116**	CL1D	L	HS	18S	889	Uncultured marine fungus clone FAS_22	98	GQ120119.1	Capnodiales	MH791176
						Cladosporium cladosporioides strain 095		MF072585.1		
						Cladosporium cladosporioides strain 090		MF072584.1		
**117**	49WS1B	SC	GPY	18S	1538	Pleosporales sp. MF565	98	KM096358.1	Pleosporales	MH791180
						Pleosporales sp. MF552		KM096350.1		
						Pleosporales sp. MF550		KM096348.1		
**118**	NWS1A	SC	PCA	ITS	300	*Epicoccum nigrum* isolate MFLUCC17-1162	100	MG807029.1	Pleosporales	MH791263
						Epicoccum nigrum isolate MFLUCC17-1153		MG807028.1		
						Epicoccum nigrum isolate MFLUCC17-1214		MG807027.1		
**119**	20WA1B	WO	GPY	18S	886	Hypocreales sp. LF213	99	KM096182.1	Hypocreales	MH791190
						Cosmospora sp. 3PC-2011 strain GJS96186		JN939679.1		
						Cosmospora sp. 3PC-2011 strain GJS95199		JN939678.1		
**120**	31XWF2	WO	HS	ITS	403	*Aspergillus ruber* strain MHS9	100	KY629639.1	Eurotiales	MH791289
						Aspergillus chevalieri strain M-1		MF324891.1		
						Aspergillus chevalieri strain M-6		MF324890.1		
**121**	41FSA	SD	PCA	ITS	291	*Epicoccum nigrum* isolate MFLUCC17-1162	100	MG807029.1	Pleosporales	MH791264
						Epicoccum nigrum isolate MFLUCC17-1153		MG807028.1		
						Epicoccum nigrum isolate MFLUCC17-1214		MG807027.1		
**122**	B1F1B	F	WSP	ITS	374	Fungal sp. isolate ZY-58	100	KY040255.1	Glomerellales	MH791265
						Plectosphaerella sp. IwLs3		LC222413.1		
						Plectosphaerella sp. Jul1b		LC222408.1		
**123**	B2F1B	F	WSP	ITS	486	Fungal sp. isolate ZY-58	99	KY040255.1	Glomerellales	MH791266
						Plectosphaerella cucumerina isolate VGPC14-2		MF688853.1		
						Plectosphaerella sp. isolate LG1401_GL10B		KX359601.1		

Supplementary Table 2. Putatively identified compounds by dereplication of extracts using GNPS combined with an *in silico* MS/MS database (ISDB-UNPD) and manual dereplication based on DNP, REAXYS, MarinLit and SciFinder databases. Annotation considered the *m/z* $[M+H]^+$, biological source, retention time, predicted molecular formula and fragmentation pattern. Culture type; N: metabolite derived from a mono-culture, C: metabolite derived from co-culture.

No.	Putative ID/ chemical family	Structure	Molecular formula of the *m/z* $[M+H]^+$ (Δ ppm)	Parent mass *m/z* $[M+H]^+$	MS/MS	Retention time(min)/ Culture type
**1**	Cephalochromin<br><br>(naphtho-γ-pyrone)	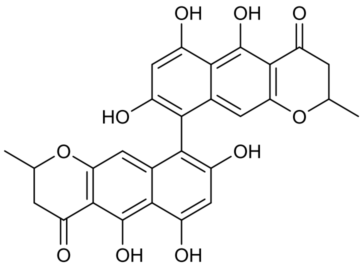	$C_{28}H_{23}O_{10}$<br><br>(1.3)	519.1284	501.1195, 260.0698, 245.0457, 231.0683, 219.0297, 218.0229	7.46<br><br>N/C
**2**	Ustilaginoidin G/ Dihydroisoustilaginoidin A<br><br>(naphtho-γ-pyrone)	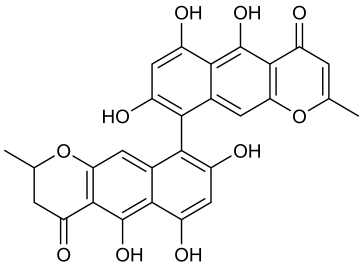	$C_{28}H_{21}O_{10}$<br><br>(2.9)	517.1150	499.1036, 260.0694, 258.0541, 245.0427, 231.0701, 219.0291	7.31<br><br>N/C

**3**	Ustilaginoidin A<br>(naphtho-γ-pyrone)	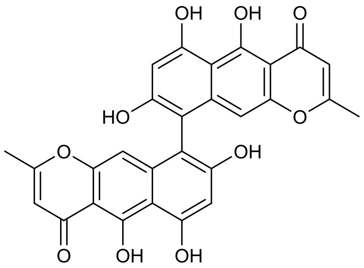	$C_{28}H_{19}O_{10}$<br>(-2.1)	515.1259	-	4.69<br>N/C
**4**	Ustilaginoidin I<br>(naphtho-γ-pyrone)	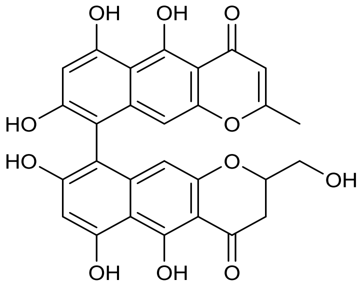	$C_{28}H_{21}O_{11}$<br>(1.1)	533.1090	515.0963, 493.1126, 477.0819, 451.0999, 260.0693	6.12<br>N/C
**5**	Ustilaginoidin V<br>(naphtho-γ-pyrone)	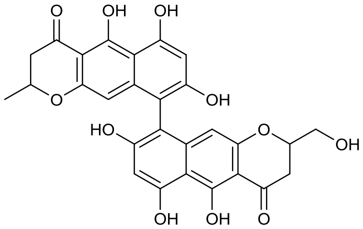	$C_{28}H_{23}O_{11}$<br>(-0.4)	535.1237	517.1116, 493.1128, 477.0807, 451.1015, 260.0688, 517.1116, 493.1128, 477.0807, 451.1015, 260.0688	6.12<br>N/C
**6**	Ustilaginoidin E<br>(naphtho-γ-pyrone)	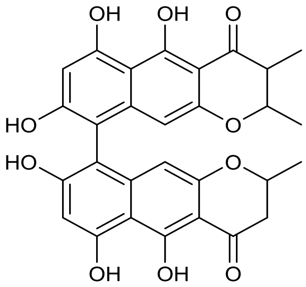	$C_{29}H_{25}O_{10}$<br>(3.1)	533.1479	515.1333, 490.1250, 477.0801, 274.0849, 260.0694	7.84<br>N/C

**7**	Chaetochromin A or Ustilaginoidin D<br><br>(naphtho-γ-pyrone)	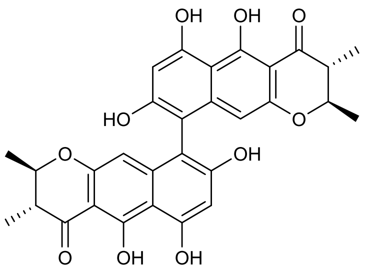	$C_{30}H_{27}O_{10}$<br><br>(-2.7)	547.1599	529.1428, 504.1302, 491.1051, 274.0846, 201.0479	8.25<br><br>N/C
**8**	Chaetochromin A; Stereoisomer, 2',3'-didehydro<br><br>(naphtho-γ-pyrone)	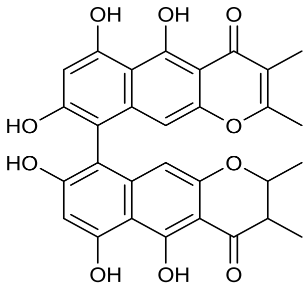	$C_{30}H_{25}O_{10}$<br><br>(-0.1)	545.1447	464.2164, 424.1467, 272.0674	8.62<br><br>N/C
**9**	Acuminatum C<br><br>(cyclodepsipeptide)	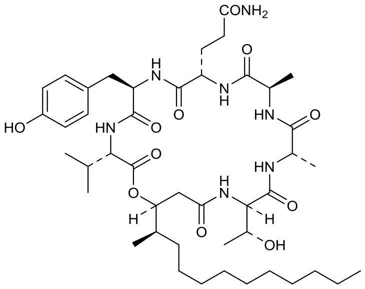	$C_{44}H_{72}N_7O_{11}$<br><br>(4.3)	874.546	856.5190, 551.2844, 466.3281, 434.2038, 395.2917, 377.2818, 271.1421, 200.1040, 101.0707	8.14<br><br>N/C

**10**	Acuminatum B<br><br>(cyclodepsipeptide)	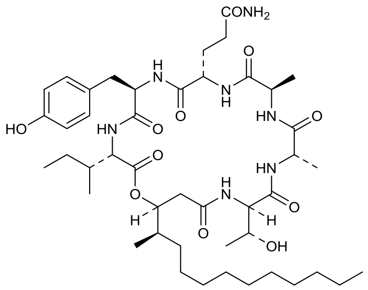	$C_{45}H_{74}N_7O_{11}$<br><br>(0.7)	888.5449	870.5326, 565.2996, 466.3303, 434.2056, 395.2923, 271.1425	8.44<br><br>N/C
**11**	3-Hydroxy-7(11),9-eremophiladien-8-one; 3β-form, 3-Ac<br><br>(bicyclic sesquiterpenoid)	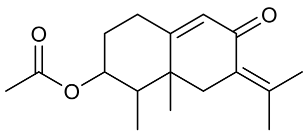	$C_{17}H_{25}O_3$<br><br>(1.4)	277.1808	259.1704, 151.0759	5.24<br><br>N/C
**12**	3,6-Dihydroxy-7(11),9-eremophiladien-8-one; (3α,6α)-form, 3-Ac<br><br>(bicyclic sesquiterpenoid)	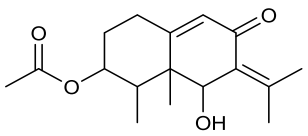	$C_{17}H_{25}O_4$<br><br>(1)	293.1756	275.1643, 257.1547, 151.0760	5.37<br><br>N/C
**13**	Zervamicin ZIIB<br><br>(peptaibol)	Ac-Trp-Ile-Gln-Iva-Ile-Thr-Aib-Leu-Aib	Corresponds to 9 amino acid sequence of Zervamicin ZIIB	1066.5811	981.5859, 850.4821, 783.4412, 682.3937, 569.3098, 470.2407, 342.1846	7.13<br><br>N

**14**	Helvolic acid<br><br>(nortriterpenoid)	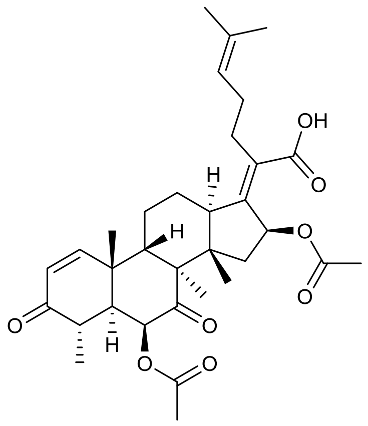	$C_{31}H_{41}O_6$<br><br>(-0.2)	Calculated for 509.2906	491.2789, 463.2843, 449.2691, 403.2646, 385.2529, 213.1283, 177.0921, 139.0759	7.87<br><br>N/C
**15**	Zervamicin ZIC<br><br>(peptaibol)	Ac-Trp-Ile-Glu-Iva-Ile-Thr-Aib-Leu-Aib-Hyp-Gln-Aib-Hyp-Aib	Correspond to 14 amino acid sequence of Zervamicin ZIC	1592.9006	1067.6276, 982.5656, 851.4849, 683.3985, 570.3151, 471.2359, 342.1906	7.10<br><br>N/C
**16**	Emerimicin_IV<br><br>(peptaibol)	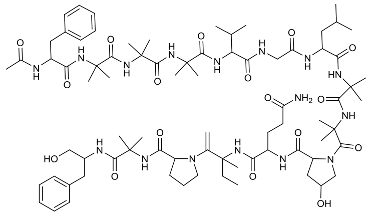	$C_{77}H_{121}N_{16}O_{19}$<br><br>(2)	1573.8994	1224.6918, 884.5237, 799.4716, 714.4200, 544.3147, 445.2454, 360.1936	7.21<br><br>N/C
**17**	Heptaibin<br><br>(peptaibol)	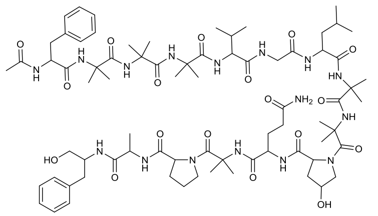	$C_{76}H_{119}N_{16}O_{19}$<br><br>(1.4)	1559.8879	1210.6702, 884.5254, 799.4731, 714.4203, 544.3164, 445.2562, 360.1929	6.92<br><br>N/C

**18**	Zervamicin ZIIB<br>(peptaibol)	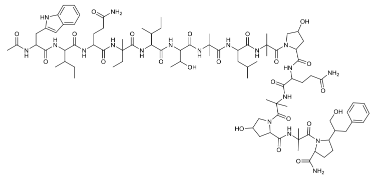	$C_{76}H_{120}N_{17}O_{20}$<br>(0.2)	1590.8916	1066.6288, 850.4811, 682.3939, 569.3097, 525.2674, 470.2415, 440.2150, 327.1674, 249.1608, 152.1079	7.12<br>N/C
**19**	Cephalosporin C<br>(β-lactam)	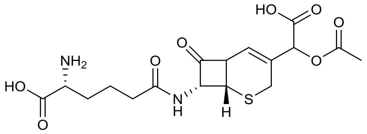	$C_{16}H_{22}N_3O_8$ S<br>(0.5)	416.1130		2.71<br>C
**20**	4-methoxybenzoic acid<br>(benzenoid)	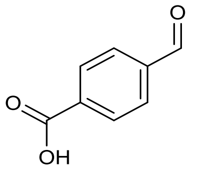	$C_8H_7O_3$<br>(-1.3)	151.0393	123.0439	4.85<br>N/C
**21**	Mitorubric acid<br>(azaphilone)	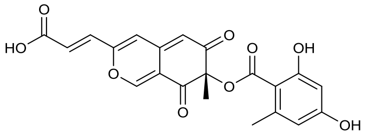	$C_{21}H_{17}O_9$<br>(-1)	413.1085	263.0558, 245.0469,151.0392	5.06<br>N/C
**22**	Mitorubrinol<br>(azaphilone)	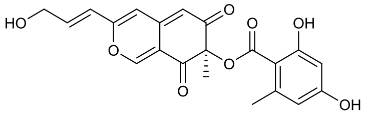	$C_{21}H_{19}O_8$<br>(0.3)	399.1081	249.0766, 231.0664, 151.0394	4.85<br>N/C
**23**	Mitorubrin; 3'-Acetoxy<br>(azaphilone)	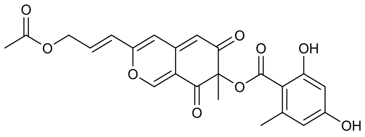	$C_{23}H_{21}O_9$<br>(0.2)	441.1187	291.0876, 273.0766, 231.0668, 151.0397	6.16<br>N/C

**24**	Mitorubrin<br><br>(azaphilone)	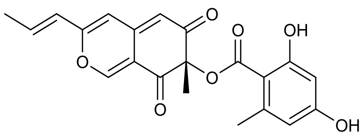	$C_{21}H_{19}O_7$<br><br>(0)	383.1132	233.0821, 215.0715, 151.0400	6.62<br><br>N/C
**25**	Mitorubrin ;3' – acetoxy,3,4-dihydro<br><br>(azaphilone)	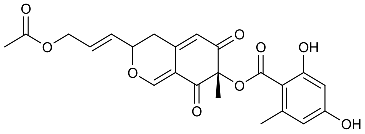	$C_{23}H_{23}O_9$<br><br>(0)	443.1342	293.1012, 151.0389	6.3<br><br>N
**26**	Sclerosporin<br><br>(sesquiterpene)	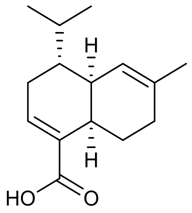	C_{15} H_{23} O_2<br><br>(3.4)	235.1706	-	5.2<br><br>N/C
**27**	Hydroxysclerosporin<br><br>(sesquiterpene)	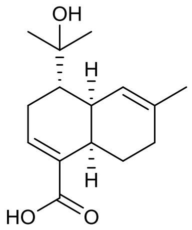	$C_{15}H_{23}O_3$<br><br>(0.8)	251.1685	-	3.08<br><br>C
**28**	Hypoxyxylerone<br><br>(xanthenone)	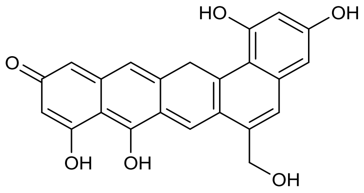	$C_{22}H_{15}O_7$<br><br>(-0.8)	391.0815	375.2151, 245.0477, 149.0225	5.08<br><br>N/C

**29**	7-hydroxy-16,18-dimethyl-10-phenyl[11]cytochalasa-6(12),13-diene-1,21-dione<br><br>(cytochalasin)	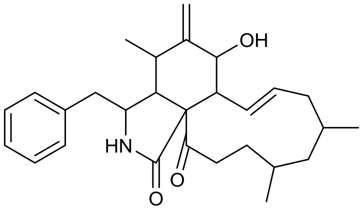	$C_{28}H_{38}NO_3$<br><br>(-0.5)	436.2850	418.2742, 400.2596, 390.2785, 308.1632, 271.2033	7.07<br><br>C
**30**	Cytochalasin J<br><br>(cytochalasin)	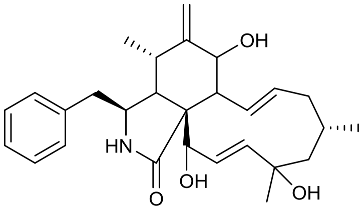	$C_{28}H_{38}NO_4$<br><br>(-0.4)	452.281	434.2694, 416.2605, 392.2578, 374.2509, 120.0797	5.85<br><br>C
**31**	Cytochalasin L 697318 , Deoxy 18B –hydroxy<br><br>(cytochalasin)	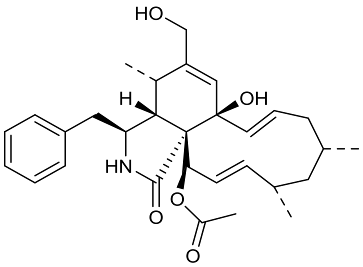	$C_{30}H_{40}NO_4$<br><br>(0.8)	478.2961	306.2810, 263.2382, 245.2268, 261.0641	8.19<br><br>N/C
**32**	Cytochalasin H<br><br>(cytochalasin)	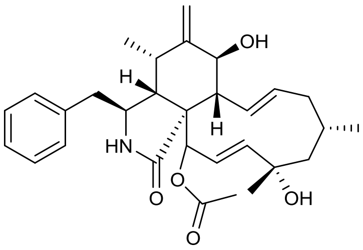	$C_{30}H_{40}NO_5$<br><br>(-0.6)	494.2903	456.2508, 434.2686, 416.2589, 398.2482, 358.2169, 308.1653, 290.1552, 251.1802, 120.0813	5.87<br><br>N/C

**33**	7-hydroxy-16,18-dimethyl-10-phenyl[11]cytochalasa-6(12),13-diene-1,21-dione<br><br>(cytochalasin)	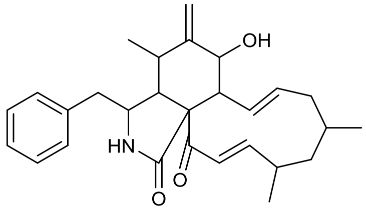	$C_{28}H_{36}NO_3$<br><br>(6.9)	434.2725	416.2589, 398.2485, 392.2592, 374.2479, 358.2162, 269.1906, 251.1810, 120.0812	5.86<br><br>N/C
**34**	1*H*-cycloundec[d] isoindole-1,15(2H)-dione,3,3a,4,6a,9,10,11, 12--3,3a,4,6a,9,10,11, 12-tetramethyl-3-(phenylmethyl)-(3S,3aR,4S,7E,10S,13E, 15aS)<br><br>(cytochalasin)	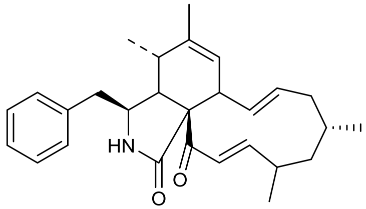	$C_{28}H_{36}NO_2$<br><br>(-0.5)	418.2750	400.2640, 346.2175, 334.2193, 252.1393, 200.1081, 172.0772, 147.1176	9.07<br><br>N/C
**35**	Cytochalasin L 697318; deoxy<br><br>(cytochalasin)	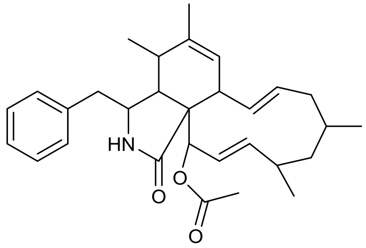	$C_{30}H_{40}NO_3$<br><br>(3)	462.3007	424.2617, 402.2786, 374.2862, 332.1990, 320.2102, 292.1707, 268.1733, 255.2106, 172.0762, 145.1010, 120.0820	10.4<br><br>N/C
**36**	1*H*-Cycloundec[*d*] isoindole, [11] cytochalasa-6(12),13,19-trien-1-one deriv. (cytochalasin)	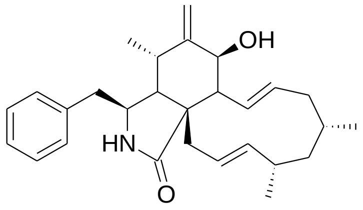	$C_{28}H_{34}NO_2$<br><br>(-0.5)	416.2585	398.2484, 374.2482, 360.1965, 269.1909, 251.1804, 172.0759, 120.0814	6.13<br><br>N/C

**37**	Sclerin<br><br>(isochromene)	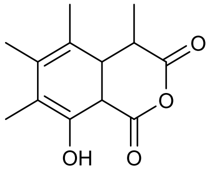	$C_{15}H_{23}O_2$<br><br>(2.6)	235.1704	217.1588, 173.1330, 147.1185, 121.1015, 109.1016, 95.0857	5.92<br><br>N/C
**38**	Iso-ochracein<br><br>(benzofuranone)	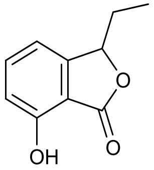	$C_{10}H_{11}O_3$<br><br>(-1.7)	179.0675	161.0601, 141.9589, 139.9877, 133.0652, 97.9689, 151.0749	5.26<br><br>N/C
**39**	Ergosta-4,6,8,22-tetraene-3-one<br><br>(steroid)	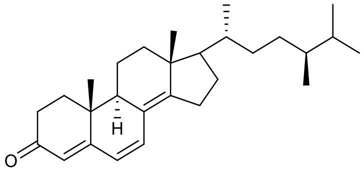	$C_{28}H_{41}O$<br><br>(3.8)	393.3172	-	10.9<br><br>N/C
**40**	Spiroindicumide B<br><br>(spirolactone polyketide)	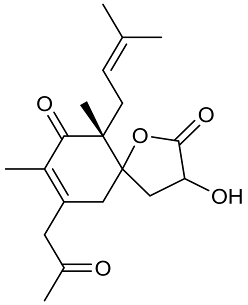	$C_{19}H_{27}O_5$<br><br>(-32.6)*	335.1532	-	9.84<br><br>C
**41**	Tenuazonic acid<br><br>(pyrrolone)	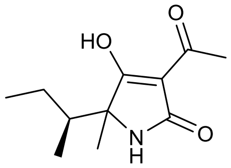	$C_{10}H_{16}NO_3$<br><br>(1)	198.1132	181.0872, 158.0032, 153.0917, 142.0505, 125.0239, 116.9765, 96.9615	4.59<br><br>N/C

**42**	Alternariol<br><br>(benzocoumarin)	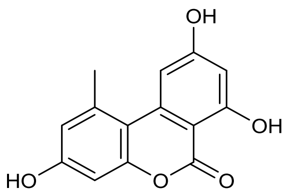	$C_{14}H_{11}O_5$<br><br>(1.2)	259.0609	241.0513, 231.0673, 217.0515, 213.0552, 191.0712, 187.0761	5.03<br><br>N/C
**43**	Alternuisol<br><br>(benzocoumarin)	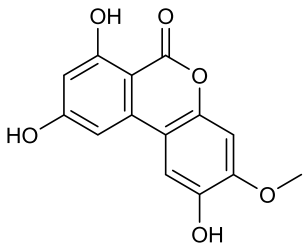	$C_{14}H_{11}O_6$<br><br>(0.7)	275.0558	257.0395, 234.9118	5.35<br><br>N/C
**44**	Altenuene<br><br>(benzocoumarin)	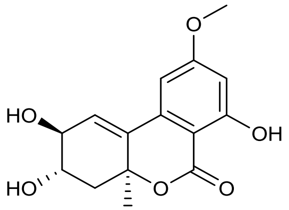	$C_{15}H_{17}O_6$<br><br>(1.4)	293.1029	275.0919, 257.0820, 239.0712, 229.0868	4.26<br><br>N/C
**45**	Alternariol: 9- Me ether<br><br>(benzocoumarin)	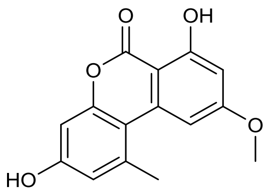	$C_{15}H_{13}O_5$<br><br>(2.2)	273.0769	255.0670, 241.0516, 227.0712, 214.0643, 197.0615	6.51<br><br>N/C

**46**	Hypochromin B<br><br>(naphtho-γ-pyrone)	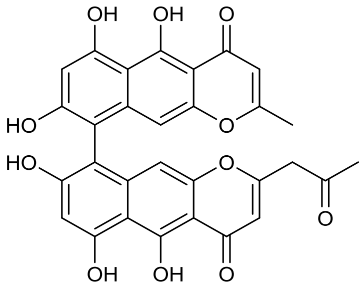	$C_{10}H_{21}O_{11}$<br><br>(0.4)	557.1086	513.1144, 495.1042, 351.0492, 307.0609, 246.0501, 218.0586, 203.0293, 151.0380	5.45<br><br>N/C
**47**	Dehydroaltenusin<br><br>(benzocoumarin)	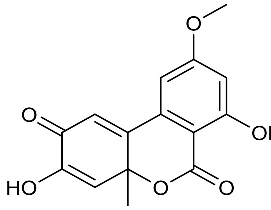	$C_{15}H_{13}O_6$<br><br>(1)	289.0715	273.1877, 250.1772, 211.0662, 206.9172, 177.1377, 167.1055, 163.1177, 159.1173	5.67<br><br>N/C
**48**	De-*O*-methylxanthomegnin<br><br>(binaphthoquinone)	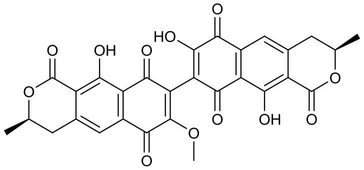	$C_{29}H_{21}O_{12}$<br><br>(0.2)	561.1034	543.092, 525.0798, 515.0967, 497.0858, 341.0285, 313.0333	6.75<br><br>N/C
**49**	Xanthomegnin<br><br>(binaphthoquinone)	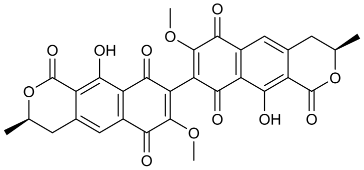	$C_{30}H_{23}O_{12}$<br><br>(5.4)	575.1221		5.47<br><br>(N/C)
**50**	3-benzyl-6-benzylidene-piperazine-2,5-dione<br><br>(diketopiperazine)	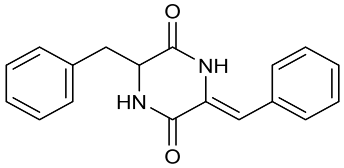	$C_{18}H_{17}N_2O_2$<br><br>(-43.7)*	293.1160	264.1031, 236.1138, 193.1021, 179.0825, 162.0561, 144.0487, 134.0624, 118.0654, 91.0541	4.17<br><br>C

**51**	3,6-dibenzyl-2,5-piperazinedione<br><br>(diketopiperazine)	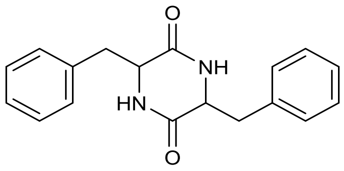	$C_{18}H_{19}N_2O_2$<br><br>(-43.4)*	295.1303		4.17<br><br>C
**52**	Stachybotrydial<br><br>(spirocyclic drimanes)	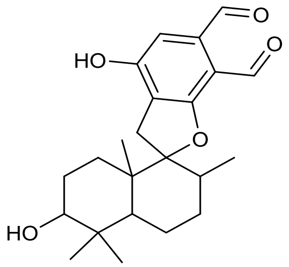	$C_{23}H_{31}O_5$<br><br>(30.7)*	387.229	368.3897, 251.2700, 338.3758, 239.1426, 177.0921, 123.1175	9.76<br><br>N/C
**53**	cyclo{L-alanyl-D-alanyl-[(2S,9S)-2-amino-9-hydroxy-8-oxodecanoyl]-D-prolyl} (JM47)<br><br>(cyclic tetrapeptide)	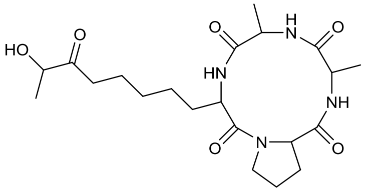	$C_{21}H_{35}N_4O_6$<br><br>(-29.8)*	439.2395	422.2732, 399.2073, 366.1740, 281.1512, 132.0997	5.12<br><br>N/C
**54**	cyclo-(alanyl->alanyl->prolyl->2-amino-9,10-epoxy-8-oxo-decanoyl)|cyclo<(2-amino-9,10-epoxy-8-oxodecanoyl)-alanyl-alanyl-prolyl<br><br>(cyclic tetrapeptide)	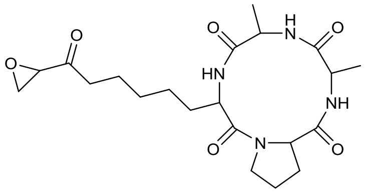	$C_{21}H_{33}N_4O_6$<br><br>(-23.6)*	437.2296	419.2223, 397.1274, 365.1661, 347.1664, 319.1791, 281.1640, 125.0974	5.46<br><br>N/C

**55**	Emericolin D<br><br>(sesterterpene)	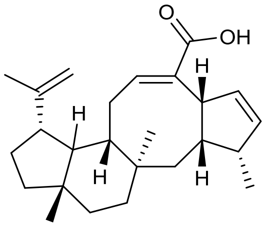	$C_{25}H_{37}O_2$<br><br>(0.3)	369.2785	351.2686, 323.2741, 257.1545, 177.0915, 109.1017	8.33<br><br>N/C
**56**	Emericolin A<br><br>(sesterterpene)	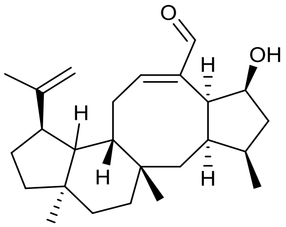	$C_{25}H_{39}O_2$<br><br>(-0.8)	371.294	353.2844, 325.2898, 315.2339, 269.2280, 213.1642, 193.1956, 159.1173	10.48<br><br>N/C
**57**	Pyrenolide C<br><br>(keto-lactone)	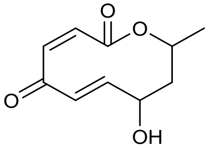	$C_{10}H_{13}O_4$<br><br>(2.5)	197.0819	-	2.26<br><br>N
**58**	8-deoxyophiobolin J<br><br>(ophiobolin)	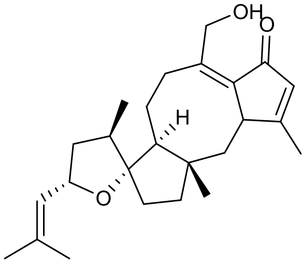	$C_{25}H_{37}O_3$<br><br>(-0.3)	385.2742	367.2634, 349.2538, 307.2069; 201.1648	9.28<br><br>N/C

**59**	Ophiobolin G<br><br>(ophiobolin)	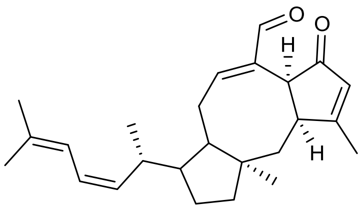	$C_{25}H_{35}O_2$<br><br>(-0.8)	367.2621	-	8.25<br><br>N/C
**60**	Ophiobolin T<br><br>(ophiobolin)	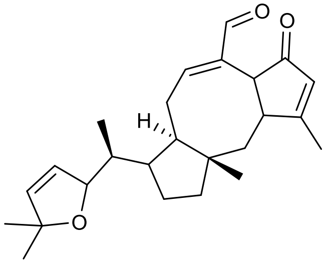	$C_{25}H_{35}O_3$<br><br>(-6.8)	383.2549	-	5.28<br><br>C
**61**	Ophiobolin A; 3-Deoxy, 3,4-didehydro, 6β-hydroxy<br><br>(ophiobolin)	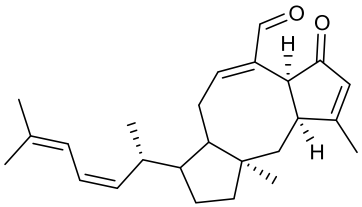	$C_{25}H_{35}O_4$<br><br>(0)	399.2540	381.2426, 363.2315, 345.2189, 279.1754, 159.1177, 145.1012	5.25<br><br>N/C
**62**	Cyclocitrinol; 12β-Hydroxy<br><br>(steroid with bicyclic ring)	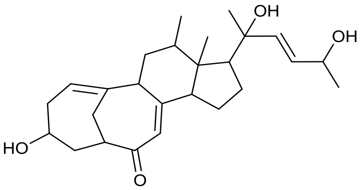	$C_{25}H_{37}O_5$<br><br>(-4.1)	417.2624	-	5.06<br><br>N

*Peak ions of low intensity (molecular formula prediction with high ppm errors)
